# Metabolomics biomarkers of hepatocellular carcinoma in a prospective cohort of patients with cirrhosis

**DOI:** 10.1016/j.jhepr.2024.101119

**Published:** 2024-05-15

**Authors:** Jessica I. Sanchez, Antoine C. Fontillas, Suet-Ying Kwan, Caren I. Sanchez, Tiffany L. Calderone, Jana L. Lee, Ahmed Elsaiey, Darrel W. Cleere, Peng Wei, John M. Vierling, David W. Victor, Laura Beretta

**Affiliations:** 1 Department of Molecular and Cellular Oncology, The University of Texas MD Anderson Cancer Center, Houston, TX, USA; 2 Margaret M. and Albert B. Alkek Department of Medicine, Section of Gastroenterology and Hepatology, Baylor College of Medicine, Houston, TX, USA; 3 Department of Gastroenterology, Houston Methodist Hospital, Houston, TX, USA; 4 Department of Biostatistics, The University of Texas MD Anderson Cancer Center, Houston, TX, USA

**Keywords:** Hepatocellular carcinoma, Cirrhosis, Surveillance, Biomarkers, Metabolomics

## Abstract

**Background & Aims:**

The effectiveness of surveillance for hepatocellular carcinoma (HCC) in patients with cirrhosis is limited, due to inadequate risk stratification and suboptimal performance of current screening modalities.

**Methods:**

We developed a multicenter prospective cohort of patients with cirrhosis undergoing surveillance with MRI and applied global untargeted metabolomics to 612 longitudinal serum samples from 203 patients. Among them, 37 developed HCC during follow-up.

**Results:**

We identified 150 metabolites with significant abundance changes in samples collected prior to HCC (Cases) compared to samples from patients who did not develop HCC (Controls). Tauro-conjugated bile acids and gamma-glutamyl amino acids were increased, while acyl-cholines and deoxycholate derivatives were decreased. Seven amino acids including serine and alanine had strong associations with HCC risk, while strong protective effects were observed for N-acetylglycine and glycerophosphorylcholine. Machine learning using the 150 metabolites, age, gender, and *PNPLA3* and *TMS6SF2* single nucleotide polymorphisms, identified 15 variables giving optimal performance. Among them, N-acetylglycine had the highest AUC in discriminating Cases and Controls. When restricting Cases to samples collected within 1 year prior to HCC (Cases-12M), additional metabolites including microbiota-derived metabolites were identified. The combination of the top six variables identified by machine learning (alpha-fetoprotein, 6-bromotryptophan, N-acetylglycine, salicyluric glucuronide, testosterone sulfate and age) had good performance in discriminating Cases-12M from Controls (AUC 0.88, 95% CI 0.83-0.93). Finally, 23 metabolites distinguished Cases with LI-RADS-3 lesions from Controls with LI-RADS-3 lesions, with reduced abundance of acyl-cholines and glycerophosphorylcholine-related lysophospholipids in Cases.

**Conclusions:**

This study identified N-acetylglycine, amino acids, bile acids and choline-derived metabolites as biomarkers of HCC risk, and microbiota-derived metabolites as contributors to HCC development.

**Impact and implications:**

The effectiveness of surveillance for hepatocellular carcinoma (HCC) in patients with cirrhosis is limited. There is an urgent need for improvement in risk stratification and new screening modalities, particularly blood biomarkers. Longitudinal collection of paired blood samples and MRI images from patients with cirrhosis is particularly valuable in assessing how early blood and imaging markers become positive during the period when lesions are observed to obtain a diagnosis of HCC. We generated a multicenter prospective cohort of patients with cirrhosis under surveillance with contrast MRI, applied untargeted metabolomics on 612 serum samples from 203 patients and identified metabolites associated with risk of HCC development. Such biomarkers may significantly improve early-stage HCC detection for patients with cirrhosis undergoing HCC surveillance, a critical step to increasing curative treatment opportunities and reducing mortality.

## Highlights

• Metabolomics performed on 612 longitudinal serum samples from 203 patients under MRI surveillance; 37 patients developed HCC.
• Serine and alanine were strongly associated with HCC risk, while N-acetylglycine and GPC had strong protective effects.
• A panel of six markers had good performance in discriminating Cases within 1 year prior to HCC diagnosis from Controls.
• Lower acyl-cholines and glycerophosphorylcholines observed in Cases with LI-RADS-3 lesions *vs.* Controls with LI-RADS-3 lesions.
• Microbiota-derived metabolites are major contributors to HCC development.

## Graphical abstract

**Figure:**
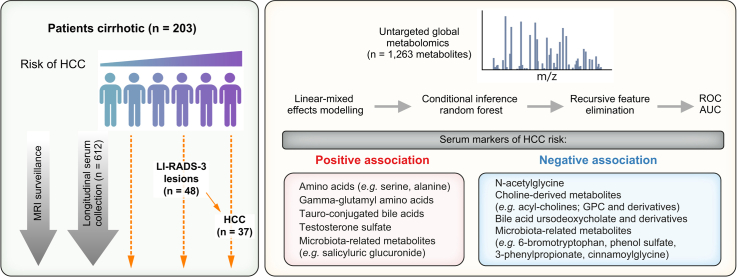

## Introduction

Liver cancer is a major cause of death globally, and the number of people diagnosed with liver cancer is predicted to rise by 55% between 2020 and 2040.^1^ In the United States, mortality rates for hepatocellular carcinoma (HCC) have started to decline, although HCC incidence continues to increase in over half of states, due in part to racial/ethnic disparity.^2^^,^^3^ While the overall survival of patients with HCC has improved over recent decades, with increasing detection of localized HCC,^2^ the overall prognosis of HCC remains dismal. To improve early diagnosis and access to potentially curative therapies, HCC surveillance based on semiannual liver ultrasound with or without serum alpha-fetoprotein (AFP) is recommended in patients with cirrhosis or chronic hepatitis B.^4^ However, the effectiveness of HCC surveillance is limited by inadequate risk stratification and suboptimal performance of the current screening modalities for the detection of early-stage tumors.^5^^,^^6^

Promising new tools for HCC surveillance have been introduced in recent years. The detection rate of focal liver lesions is excellent with contrast-enhanced MRI, making it the best alternative to ultrasound for surveillance.^7^^,^^8^ However, performing a full contrast-enhanced MRI for HCC surveillance in the currently defined risk populations is unrealistic due to availability and cost. Alternative imaging surveillance modalities include non-contrast MRI^9^^,^^10^ and abbreviated MRI.[11], [12], [13] AFP remains the most widely used serum marker, despite its poor performance. Osteopontin has been identified as a promising serological biomarker for early detection of HCC and is highly complementary to AFP,[14], [15], [16], [17] while proteome multimarker panels have also been proposed.[18], [19], [20] Meanwhile, cell-free DNA methylation patterns and extracellular vesicle markers have evolved as promising surveillance tools for early HCC detection in populations at risk.[21], [22], [23], [24], [25], [26], [27] Finally, algorithms and risk scores have also been developed. The performance of the GALAD score to detect early-stage HCC remains controversial.[28], [29], [30] Other scores, such as the HCC risk score aMAP and the Toronto HCC risk index (THRI), were developed to discriminate between low- and high-risk patients.[31], [32], [33] The combination of clinical and genetic predictors, such as patatin-like phospholipase domain-containing protein 3 (*PNPLA3*) and transmembrane 6 superfamily member 2 (*TM6SF2*) single nucleotide polymorphisms (SNPs), may improve HCC risk stratification.^34^^,^^35^ Additional innovative approaches to non-invasively diagnose early HCC include gut and circulating targeted microbiota,^36^^,^^37^ viral exposure signatures^38^ and cell-free DNA fragmentomics features.^39^

Considering the rapidly changing epidemiology of HCC and the effects of etiology, age and gender on risk of HCC in cirrhosis, a "personalized" approach to surveillance is gaining growing support. In this context, novel biomarkers are needed both to stratify patients into low- and high-risk groups and to screen high-risk patients for detection of early-stage HCC. These biomarkers need to be evaluated in prospective cohorts of at-risk individuals under surveillance with imaging allowing for detection of small lesions and high sensitivity for early stage HCC. We therefore developed a multi-center prospective cohort of patients with cirrhosis undergoing surveillance with contrast-enhanced MRI and applied comprehensive metabolomics profiling to a large number of longitudinal samples collected from patients who developed or did not develop HCC during surveillance.

## Patients and methods

### Patient cohort

This study was conducted in accordance with the Declaration of Helsinki and approved by the Institutional review Board of all participating institutions. At recruitment, written informed consent was obtained from each participant. A nested group of 203 patients with cirrhosis under surveillance for HCC with contract-enhanced MRI were selected from a large multicenter prospective cohort initiated in 2017. The recruitment sites for this nested cohort study included Houston Methodist Hospital (Site 1: n = 108) and Baylor College of Medicine (Site 2: n = 95). All participants were followed during the duration of the study with contrast-enhanced MRI and blood collection, at time intervals defined by their surveillance standard of care at the participating institutions. The median duration of follow-up was 4.2 years, ranging from 1.9 to 4.8 years. Pre-HCC and HCC lesions were classified by the Liver Imaging Reporting and Data System (LI-RADS) classification system. Among the 203 patients, 38 developed liver cancer during follow-up (37 HCC and 1 cholangiocarcinoma). At enrollment and at each follow-up visit, one lavender and two red top venous blood tubes were collected from each patient. Serum, plasma and buffy coat aliquots were then stored in −80 °C. Detailed information on eligibility criteria, collected demographic and clinical parameters, HCC characteristics and secure data capture system are available in the Supplementary materials and methods.

### Global metabolomics profiling

A total of 612 serum samples (250 μl) collected from the 203 patients, with an average of three prospectively collected samples per patient and up to 11 samples, were submitted for global metabolomics profiling (Metabolon Inc, Durham, NC). The median time between successively collected samples was 5.9 months. Sample preparation and processing, and raw data analysis are described in detail in the Supplementary materials and methods.

### DNA extraction and SNP genotyping

Genomic DNA was extracted from 200 μl of buffy coat using the QIAamp DNA blood mini kit (Qiagen Co. Ltd., DE, Düsseldorf, Germany). *PNPLA3* rs738409 and *TM6SF2* rs58542926 were genotyped by TaqMan 5'-nuclease assays using predesigned TaqMan probes (Applied Biosystems, Foster City, CA), on a ViiA7 Real time PCR system (Applied Biosystems, Foster City, CA).

### Statistical analyses

All statistical analyses are described in detail in the Supplementary materials and methods.

## Results

### Nested cohort study of patients with cirrhosis under HCC surveillance by contrast MRI

In this study, we used a nested cohort of 203 patients selected from two sites in a multicenter prospective cohort of patients with cirrhosis under surveillance for HCC by contrast MRI. The demographic and clinical parameters of the study participants at enrollment are shown in Table S1. Males and females were equally distributed and most patients were non-Hispanic White. The median age (61), BMI (30.8) and presence of diabetes (45%) at recruitment were similar at both sites. The most common etiologies, MASLD (38%) and HCV (31%), were similarly observed in patients from both sites. Alcohol was an important etiology in patients from Site 1 (35%). A significant difference in Child Pugh class was observed between the two sites (*p* <0.001), with a majority of class A (68%) at Site 2 and a majority of class B (66%) at Site 1. Among the 203 patients, 38 developed liver cancer during follow-up (37 HCC and 1 cholangiocarcinoma). Overall, patients who developed liver cancer were significantly older with a median age of 66 compared to 61, and were more likely to be diabetic (63% *vs*. 41%) (Table S1).

Clinical data, imaging and biospecimens were collected for a total of 612 visits between February 2017 and November 2021. Patient outcome was again reviewed in May 2023. Samples from the patient who developed cholangiocarcinoma were excluded for data analysis. Controls included 490 samples collected from 165 patients who never developed HCC. Cases included 88 pre-HCC samples collected from the 37 patients who developed HCC during follow-up. An additional group of 31 samples (Cases-T) were collected from seven of the patients with HCC, following treatment.

### Metabolite abundance changes associated with risk of HCC

Global metabolomics profiling was performed on all 612 serum samples collected from the 203 patients. A total of 1,263 metabolites were measured across the following super-pathways: Amino Acids (n = 227), Carbohydrates (n = 27), Cofactors and Vitamins (n = 44), Energy (n = 11), Lipids (n = 498), Nucleotides (n = 43), Peptides (n = 51), Xenobiotics (n = 333) and Partially Characterized Molecules (n = 29) (Fig. S1A). For each super-pathway, the percentages of metabolites detected in 100% of the samples and in over 75% of the samples are described in Fig. S1B.

**Fig. 1:**
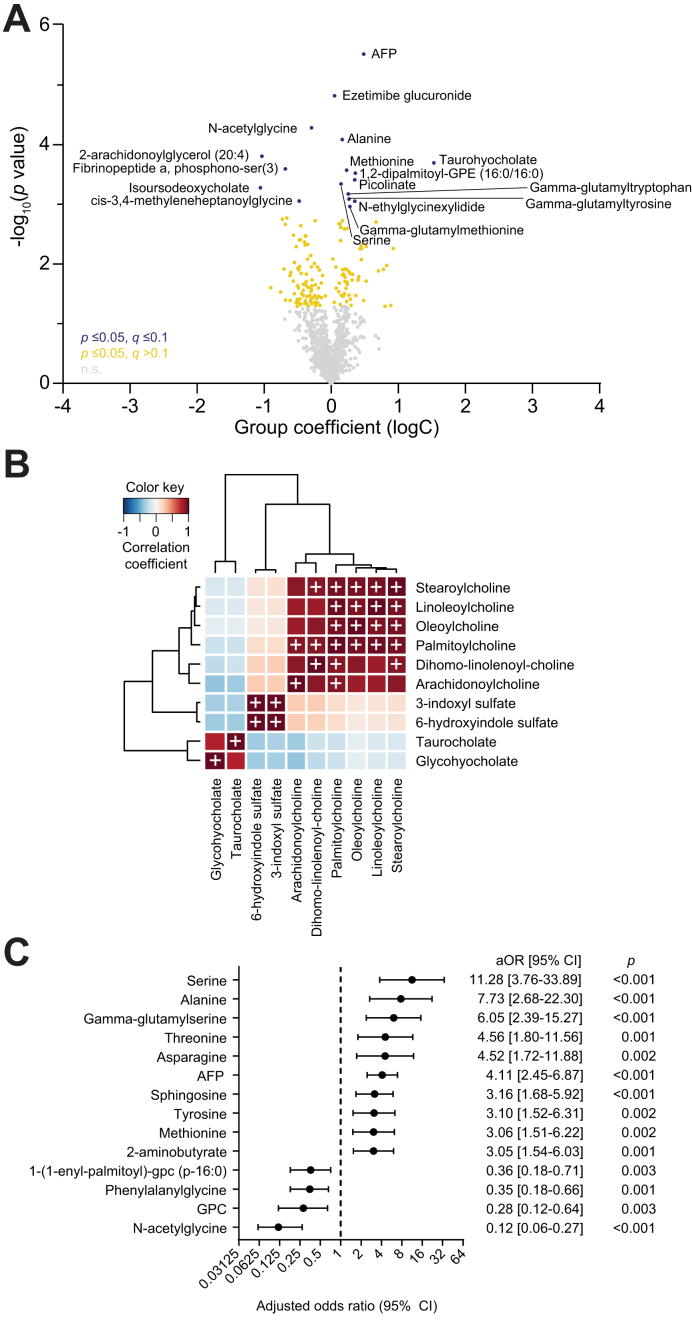
Metabolite abundance changes in Cases compared to Controls. (A) Volcano plot for differential metabolites, between cases and controls. The significance and coefficient of group (Cases *vs.* Controls) as a fixed effect on metabolite abundance was determined by linear mixed-effects modeling. Group coefficients for log-transformed metabolite abundance (x-axis) and minus log_10_*p* values (y-axis) are shown. Differential metabolites that remained significant after FDR adjustment (*p* ≤0.05, q ≤0.1) are shown in blue instead of yellow. (B) Spearman’s correlation matrix of highly correlated clusters among the 150 differential metabolites. White cross: metabolite pairs with r >0.9 and *p <*0.05. (C) Forest plot depicting the aORs [95% CI] and *p* values determined by logistic regression, for selected metabolites associated with risk of HCC, after adjusting for age, gender and diabetes. AFP, alpha-fetoprotein; aOR, adjusted odds ratio; FDR, false discovery rate; GPC, glycerophosphocholine; HCC, hepatocellular carcinoma.

We used linear mixed-effects models, incorporating time to last visit, to identify metabolites with significant abundance changes in serum samples collected prior to diagnosis in patients who developed HCC during follow-up (Cases) to samples collected from patients who did not develop HCC (Controls). AFP was also added to this analysis. AFP abundance was significantly increased in Cases compared to Controls, and remained significant after adjusting for false discovery rate (FDR) (q = 0.004). In addition, the abundance of 64 metabolites was significantly increased while the abundance of 86 metabolites was significantly decreased (Table S2; Fig. 1A). Significance remained after adjusting for FDR for 16 of these 150 metabolites (Fig. 1A). The largest increase was observed for taurohyocholate (q = 041). Four other tauro-conjugated bile acids had increases similar or higher than AFP: taurocholate (*p =* 0.005), taurochenodeoxycholate (*p =* 0.010), taurochenodeoxycholic acid 3-sulfate (*p =* 0.012) and taurocholenate sulfate (*p =* 0.005). The bile acid glycohyocholate was also strongly increased in Cases (*p =* 0.013). Several gamma-glutamyl amino acids were elevated in Cases compared to Controls: gamma-glutamylmethionine, gamma-glutamyltyrosine, gamma-glutamyltryptophan, gamma-glutamylserine, gamma-glutamylphenylalanine, and gamma-glutamyl-alpha-lysine. The largest decreases were observed for derivatives of the bile acid deoxycholate: isoursodeoxycholate (q = 0.054), ursodeoxycholate (*p =* 0.025), chenodeoxycholic acid sulfate (1) (*p =* 0.002), and isoursodeoxycholate sulfate (1) (*p =* 0.015). The pathways Androgenic Steroids, Benzoate Metabolism and Fatty Acid Metabolism (Acyl Choline) were also enriched among metabolites significantly decreased in Cases compared to Controls. Spearman’s correlation of all 150 metabolites associated with HCC (Fig. S2) identified three highly correlated clusters (r >0.9): 1) glycohyocholate and taurocholate; 2) 3-indoxyl sulfate and 6−hydroxyindole sulfate; and 3) stearoylcholine, linoleoylcholine, oleoylcholine, dihomo−linolenoyl−choline, palmitoylcholine and arachidonoylcholine (Fig. 1B). The strength of the association between abundance of the 150 metabolites or AFP with risk for HCC was further determined by logistic regression analysis, adjusting for age, gender and diabetes (Fig. 1C). While a strong association was observed for AFP (adjusted odds ratio [aOR] 4.11, 95% CI 2.45-6.87, *p <*0.001), eight metabolites including six amino acids had stronger or similar associations with HCC risk. The strongest associations were observed for serine (aOR 11.28, 95% CI 3.76-33.89, *p <*0.001), alanine (aOR 7.73, 95% CI 2.68-22.30, *p <*0.001) and gamma-glutamylserine (aOR 6.05, 95% CI 2.39-15.27, *p <*0.001). In contrast, the high abundance of four metabolites was associated with significantly reduced risk of HCC (Fig. 1C). The strongest protective effects were observed for N-acetylglycine (aOR 0.12, 95% CI 0.06-0.27, *p <*0.001) and glycerophosphorylcholine (GPC) (aOR 0.28, 95% CI 0.12-0.64, *p* = 0.003).

The inclusion of samples collected after HCC diagnosis and treatment for some of the patients who developed HCC during surveillance allowed us to determine whether any of the metabolites identified as significantly increased or decreased in Cases *vs.* Controls demonstrated a reverse phenotype after treatment (Supplementary results).

### Machine learning for HCC risk modeling

**Fig. 2:**
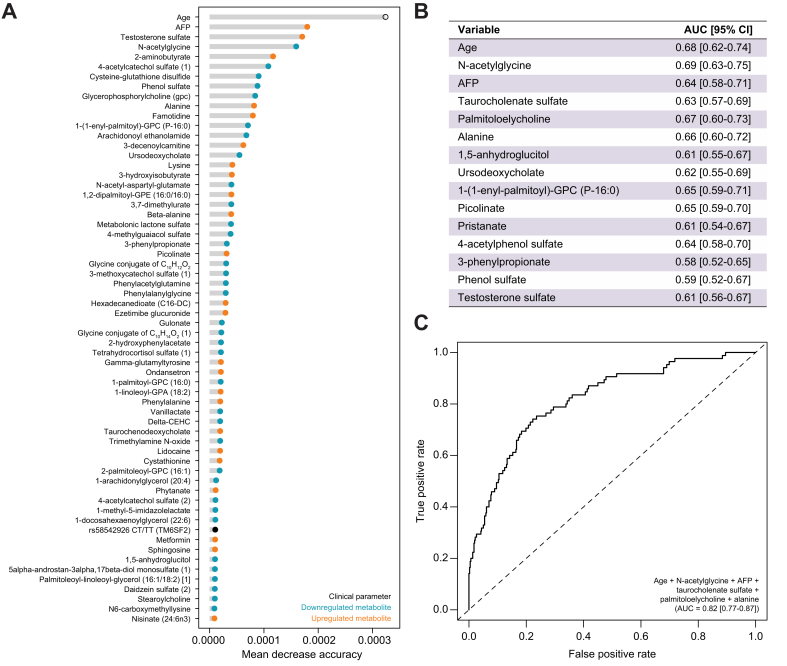
Modeling contribution of metabolites abundance, demographic and genetic parameters, in hepatocellular carcinoma prediction. (A) Conditional inference random forest was implemented. Top variables, sorted by descending importance (Mean Decrease in Accuracy), are shown. (B) Following recursive feature elimination, 15 variables were identified to give optimal model performance. Individual AUCs are shown. (C) Receiver-operating characteristic curve and AUC (95% CI) for the combination of the top six markers comparing Cases to Controls. AFP, alpha-fetoprotein; GPC, glycerophosphocholine.

To determine which among AFP and the 150 metabolites associated with risk of HCC (Table S2) contributed the most to risk prediction, the conditional inference random forest machine learning algorithm was implemented (Figs S3 and Fig. 2A). In this model, we wanted to also include age, gender and the genotypes of SNPs previously associated with HCC risk, so we genotyped *PNPLA3* rs738409 and *TMS6SF2* rs58542926 in all study participants (Fig. S4). The frequency of the *PNPLA3* rs738409 homozygous GG genotype was 21.6% in Cases and 18.2% in Controls. The frequency of the *TM6SF2* rs58542926 heterozygous CT genotype increased – but not significantly – in Cases (16.2%) compared to Controls (7.9%). The rare *TM6SF2* rs58542926 homozygous TT genotype was also detected in 2.7% and 2.4% of Cases and Controls, respectively. The conditional inference random forest model performed well in discriminating Cases and Controls (AUC 0.87, 95% CI 0.84-0.91). Based on permutation-based importance scores, age was the most important variable overall followed by AFP, testosterone sulfate and N-acetylglycine. The other demographic and genetic parameters, *TMS6SF2* rs58542926 CT/TT genotypes, *PNPLA3* rs738409 GG genotype, and gender were ranked 53th, 69st and 134st, respectively. To further determine best features of the model, we further performed feature selection by recursive feature elimination, which identified 15 variables giving optimal model performance (Fig. 2B). Among them, age and five metabolites had better individual AUCs than AFP (AUC 0.64, 95% CI 0.58-0.71), with the best AUC observed for N-acetylglycine (AUC 0.69, 95% CI 0.63-0.75) followed by age (AUC 0.68, 95% CI 0.62-0.74), palmitoloelylcholine (AUC 0.67, 95% CI 0.60-0.73), alanine (AUC 0.66, 95% CI 0.60-0.72), 1-(1-enyl-palmitoyl)-GPC (AUC 0.65, 95% CI 0.59-0.71) and picolinate (AUC 0.65, 95% CI 0.59-0.70). The top six variables were AFP, N-acetylglycine, age, alanine, taurocholenate sulfate and palmitoloelylcholine. Receiver-operating characteristic curve analysis using these top six variables showed improved performance in discriminating Cases from Controls (AUC 0.82, 95% CI 0.77-0.87) compared to AFP alone (Fig. 2C).

### Metabolite abundance changes within a year prior to HCC diagnosis and 1-year HCC risk modeling

We again used linear mixed-effects models, incorporating time to last visit, to identify metabolites with significant abundance changes in samples collected within 12 months prior to diagnosis in patients who developed HCC during follow-up (Cases-12M) to samples collected from patients who did not develop HCC (Controls). For comparison, we also used linear mixed-effects models incorporating time to last visit, to determine their abundance changes compared to Controls, in samples collected within 6 months (Cases-6M) or 24 months (Cases-24M) prior to HCC diagnosis (Table S3; Fig. S5). AFP was again added to this analysis. Ten metabolites, including the microbiota-derived metabolites salicyluric glucuronide and cinnamoylglycine, had significant abundance changes in Cases-12M but not in Cases-24M, compared to Controls, suggesting events occurring close to HCC development (Table S3; Fig. S5). AFP abundance was significantly increased in Cases-12M compared to Controls, remaining significant after adjusting for FDR, and with, as anticipated, a stronger increase than observed when comparing Cases to Controls (q <0.001). The abundance of 62 metabolites was also significantly increased while the abundance of 30 metabolites was significantly decreased (Table S3; Fig. S5). Significance remained after adjusting for FDR for 13 of these 92 metabolites (Fig. S5). The largest increase was observed again for taurohyocholate (*p <*0.001) and other tauro-conjugated bile acids had increases similar or higher than AFP: taurochenodeoxycholate (*p =* 0.012) and taurochenodeoxycholic acid 3-sulfate (*p =* 0.019). Again, the bile acid glycohyocholate was also strongly increased (*p =* 0.015). New metabolites were found strongly increased in Cases-12M compared to Controls: testosterone sulfate (*p =* 0.003) and salicyluric glucuronide (*p =* 0.035). Remarkably, for these two metabolites, the group coefficient further increased when comparing Cases-6M to Controls. The largest decreases in Cases-12M compared to Controls, were observed for isoursodeoxycholate (*p =* 0.009), as well as the microbiota-derived metabolites 3-phenylproprionate (*p =* 0.001) and cinnamoylglycine (*p* = 0.049). For these two microbiota-derived metabolites, the group coefficient further decreased in Cases-6M compared to Controls.

**Fig. 3:**
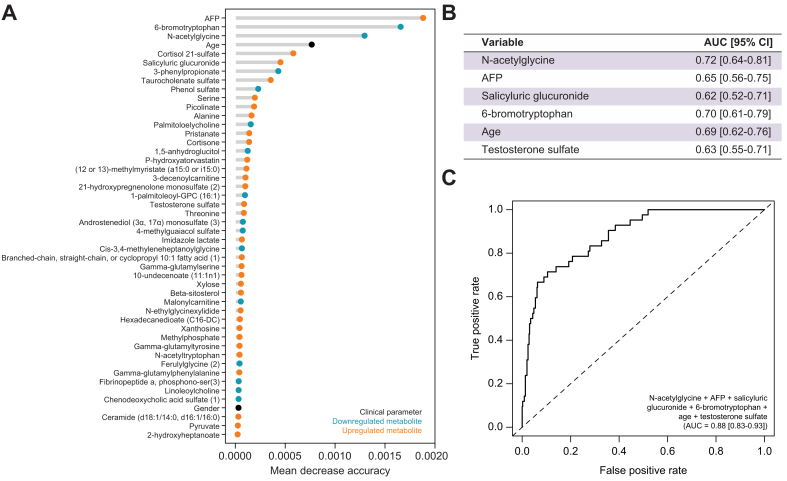
Modeling contribution of metabolites abundance, demographic and genetic parameters, in predicting hepatocellular carcinoma diagnosis within 12 months. (A) Conditional inference random forest was implemented. Top variables, sorted by descending importance (Mean Decrease in Accuracy), are shown. (B) Following recursive feature elimination, six variables were identified to give optimal model performance. Individual AUCs are shown. (C) Receiver-operating characteristic curve and AUC (95% CI) for the combination of the six markers comparing Cases-12M to Controls. AFP, alpha-fetoprotein; GPC, glycerophosphocholine.

To determine which among AFP and the 92 metabolites associated with HCC diagnosis within 12 months of follow-up contributed the most to risk prediction, conditional inference random forest was again implemented, including age, gender, *PNPLA3* rs738409 GG, and *TM6SF2* rs58542926 CT/TT genotypes (Fig S6 and Fig. 3A). The conditional inference random forest model performed very well in discriminating Cases-12M and Controls (AUC 0.92, 95% CI 0.87-0.96). AFP was the most important variable overall, followed by 6-bromotryptophan, N-acetylglycine and age. The other demographic and genetic parameters, gender, *PNPLA3* rs738409 GG, and *TMS6SF2* rs58542926 CT/TT were ranked 45th, 53rd and 65th, respectively. Feature selection by recursive feature elimination further identified six variables giving optimal model performance: N-acetylglycine, AFP, salicyluric glucuronide, 6-bromotryptophan, age and testosterone sulfate (Fig. 3B). Among them, N-acetylglycine (AUC 0.72, 95% CI 0.64-0.81), 6-bromotryptophan (AUC 0.70, 95% CI 0.61-0.79) and age (AUC 0.69, 95% CI 0.62-0.76) had better individual AUCs than AFP (AUC 0.65, 95% CI 0.56-0.75). Receiver-operating characteristic curve analysis using the six variables showed improved performance in discriminating Cases-12M from Controls (AUC 0.88, 95% CI 0.83-0.93) when compared to AFP alone (Fig. 3C).

### Clinical and demographic parameters affecting the identified HCC risk-associated metabolites

**Fig. 4:**
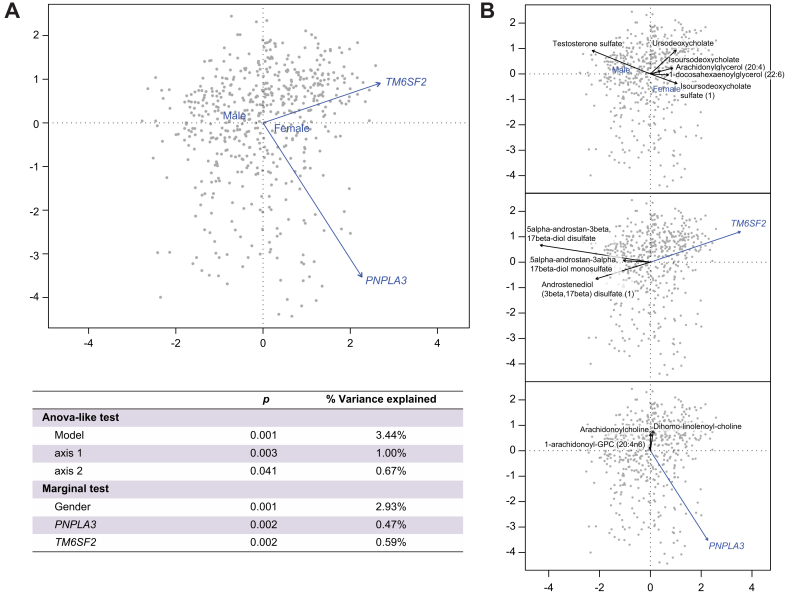
Relationship between demographic, clinical and genetic parameters and metabolites associated with hepatocellular carcinoma risk. (A) Redundancy analysis was conducted to determine the relationship between selected parameters (explanatory variables) and HCC-associated metabolite profile (response variables). The explanatory variables with the stronger effects are shown, together with ANOVA-like significance test of the model, and marginal test of the explanatory variables. (B) Redundancy analysis plots showing the relationship between gender, *TM6SF2* rs58542926 and *PNPLA3* rs738409 and selected metabolites. HCC, hepatocellular carcinoma.

We evaluated whether clinical, demographic and genetic parameters were associated with abundance changes for the HCC-associated metabolites described in Table S2. In a redundancy analysis, the metabolites were used as response variables and age, gender, etiologies and *PNPLA3* rs738409 and *TM6SF2* rs58542926 SNPs as explanatory variables. The model was statistically significant (*p =* 0.001, 9.21% variation explained) with the main contribution to variation observed for gender (2.93%, *p =* 0.001). The contribution of the two SNPs was significant although modest (*TM6SF2*: 0.59%, *p =* 0.002; *PNPLA3*: 0.47%, *p =* 0.002) (Fig. 4A). Linear mixed-effects analysis identified metabolites significantly affected by gender, *TM6SF2,* and *PNPLA3* (Table S4). The top three metabolites affected by *PNPLA3* were involved in secondary bile acid metabolism, while the top three metabolites affected by gender were related to androgenic steroids. Redundancy analysis ordination plots showed the association between select metabolites and gender, *TM6SF2*, and *PNPLA3* (Fig. 4B). Testosterone sulfate was associated with male gender (*p <*0.001), while isoursodeoxycholate sulfate (*p =* 0.005), ursodeoxycholate (*p =* 0.034), isoursodeoxycholate (*p =* 0.045), 1-arachidonylglycerol (20:4) (*p <*0.001), and 1-docosahexaenoylglycerol (*p <*0.001) were associated with female gender. Androstenediol (3beta,17beta) disulfate (*p =* 0.003), 5alpha-androstan-3beta,17beta-diol disulfate (*p =* 0.024), and 5alpha-androstan-3alpha,17beta-diol monosulfate (*p =* 0.033) were strongly negatively associated with the *TM6SF2* risk allele T. The acyl-cholines arachidonoylcholine (*p =* 0.015) and dihomo-linolenoyl-choline (*p =* 0.034), and lysophospholipid 1-arachidonoyl-GPC (20:4n6) (*p =* 0.006) were negatively associated with the *PNPLA3* risk allele G, although weakly.

### Distinguishing cases with LI-RADS-3 lesions from controls with LI-RADS-3 lesions

**Fig. 5:**
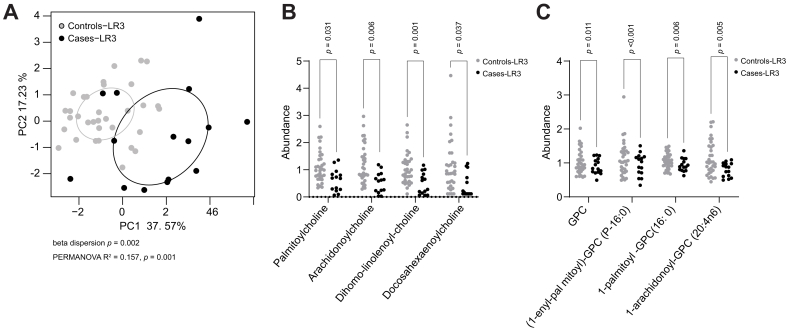
Metabolite profiles distinguishing Cases-LR3 and Controls-LR3. (A) PCA plot of 23 metabolites with significant differences in Cases-LR3 *vs*. Controls-LR3, as determined by linear mixed-effects modeling (*p <*0.05 for group as a fixed effect). (B–C) Plots showing the significant differences between Cases-LR3 and Controls-LR3 in (B) 4 acyl-cholines and, (C) GPC and GPC-derived metabolites. Significance was determined by linear mixed-effects modeling. GPC, glycerophosphocholine; PCA, principal component analysis.

Finally, we tested whether any of the HCC-associated metabolites identified (Table S2) could distinguish between Cases with LI-RADS-3 lesions who developed HCC during follow-up (Cases-LR3) and Controls with LI-RADS-3 lesions who did not develop HCC during follow-up (Controls-LR3). A total of 33 samples from Controls and 15 samples from Cases had a paired MRI showing detection of a single (n = 34), two (n = 11) or three (n = 3) LI-RADS-3 lesions, without detection of any other lesion. In addition to AFP, the abundance of nine metabolites was significantly increased in Cases-LR3, while the abundance of 13 metabolites was significantly decreased (Table S5). 1,2-dilinoleoyl-GPE (18:2/18:2) (*p =* 0.02) and malonate (*p =* 0.007) had the greatest increases, while phenolsulfate (*p =* 0.02) had the greatest decrease, in Cases-LR3 compared to Controls-LR3. Principal component analysis plots based on levels of these 23 metabolites showed a clear separation between Cases-LR3 and Controls-LR3 (PERMANOVA R^2^=0.157, *p =* 0.001) (Fig. 5A). Interestingly, the abundance of four acyl-cholines was strongly reduced in Cases-LR3 compared to Controls-LR3: dihomo-linolenoyl-choline (*p =* 0.001), arachidonoylcholine (*p =* 0.006), docosahexaenoylcholine (*p =* 0.037), and palmitoylcholine (*p =* 0.031) (Fig. 5B). GPC and GPC-related lysophospholipids were also significantly reduced in Cases-LR3 compared to Controls-LR3: GPC (*p =* 0.011), 1-(1-enyl-palmitoyl)-GPC (P-16:0) (*p <*0.001), 1-palmitoyl GPC (16:0) (*p =* 0.006) and 1-arachidonoyl-GPC (20:4n6) (*p* = 0.005) (Fig. 5C).

## Discussion

The identification of key metabolites that are associated with HCC risk in patients with cirrhosis can expand our knowledge on metabolic reprogramming leading to HCC development, and identify biomarkers for detection of early-stage HCC. To date, only a handful of metabolomics studies in HCC have been reported, including the study from Lewinska *et al.* demonstrating that a panel of serum metabolites outperformed AFP in distinguishing patients with MASLD-HCC from those without HCC.^40^ However, no metabolic studies have been performed on longitudinal biospecimens from a prospective cohort of high-risk patients with cirrhosis under surveillance for HCC. Furthermore, the multicenter prospective cohort of patients with cirrhosis we developed was based on surveillance by contrast-enhanced MRI. Such a cohort provides a unique opportunity to study blood biomarkers and imaging features on clinical material from patients rigorously classified as having very early-stage disease in a surveillance setting. The LI-RADS classification system, initially released in 2011, allows for HCC diagnosis with CT or MRI with extracellular contrast agents, as well as for pre-HCC lesion characterization.^41^ Longitudinal collection of paired blood samples and MRI images from patients with cirrhosis is particularly valuable in assessing how early blood and imaging markers become positive during the period when lesions are observed to obtain a diagnosis of HCC.

The use of untargeted metabolomics is an attractive method for biomarker discovery, although there are still limitations with this method. Unlike targeted metabolomics, untargeted metabolomics is unable to provide absolute quantification and is sensitive to sample preparation and analytical method.^42^^,^^43^

An important finding of our study is the identification of several microbiome-related metabolites, most remarkably in the year prior to HCC diagnosis, suggesting a potential direct effect of these metabolites in HCC development. Patients with HCC have a microbiome signature distinct from non-HCC controls, and evidence supports the critical role of the gut microbiome and its metabolites in influencing immune and metabolic events associated with HCC development.^44^ Bile acids have been used in panels to discriminate HCC from cirrhosis.^45^ We observed a positive association between HCC risk and the primary taurine-conjugated bile acids and their sulfated derivatives, as well as conjugated forms of the rare bile acid hyocholate. Conversely, we observed that HCC risk was associated with a decrease in the secondary bile acid ursodeoxycholate and its derivatives. While primary bile acids are synthesized and conjugated in the liver, secondary bile acids are derived from metabolism of primary bile acids by gut microbes. Ursodeoxycholate, a non-toxic hydrophilic bile acid used in the treatment of cholestatic liver disease, exhibits protective effects against HCC.^46^ Circulating levels of isoursodeoxycholate are mostly determined by the gut microbiome.^47^ We also observed an association between HCC risk and multiple amino acids. Notably, HCC risk was strongly associated with elevated levels of alanine and with low levels of several phenylalanine/tyrosine derivatives, including 2-hydroxyphenylacetate and microbiota-derived 3-phenylpropionate, phenylacetylglutamine, and phenol sulfate. Serum levels of 3-phenylpropionate and cinnamoylglycine have been associated with higher gut microbiome diversity and intestinal barrier function.^48^^,^^49^

The strongest negative association with HCC risk was observed for N-acetylglycine. N-acetylglycine outperformed AFP in all patient group comparisons performed. N-acetylglycine, a derivative of glycine, has been associated with protection against obesity and obesity-related diseases.^50^ Low levels of N-acetylglycine have been shown to mediate gut microbiome-dependent smoking cessation-induced weight gain.^51^ Supplementation of high-fat diet-fed mice with N-acetylglycine led to lower levels of the adiposity-related Trem2^+^ macrophages, and altered signaling of multiple pathways in adipose immune cells. N-acetylglycine may therefore have protective effects on HCC by modulating obesity-related immunity.^51^

Another important finding of our study is the identification of several choline-derived metabolites, mostly acyl-cholines and GPC-derived metabolites. The serum abundance of these metabolites was significantly reduced in patients with LI-RADS-3 lesions who developed HCC during follow-up, compared to patients with LI-RADS-3 lesions who did not develop HCC during follow-up. GPC is a precursor of endogenous choline and whether reduction of GPC derivatives results in reduced choline in the liver prior to HCC development should be investigated. In mice, deficiency of the lysophospholipase PNPLA7, markedly decreases hepatic GPC, choline, and several metabolites related to choline/methionine metabolism.^52^ While in our study, the *PNPLA3* SNP had only weak associations with both HCC risk and overall HCC-associated metabolite changes, the two acyl-cholines arachidonoylcholine and dihomo-linolenoyl-choline, and the lysophospholipid 1-arachidonoyl-GPC (20:4n6) were negatively associated with the *PNPLA3* rs738409 risk allele G.

Finally, AFP showed good performance even years before HCC diagnosis in agreement with a prior report that serum AFP levels increase more than 10 years before detection of HCC.^53^ This suggests that AFP could be used together with some of the metabolites identified, to assign patients to high-risk *vs.* low-risk groups for surveillance, helping the field move toward precision surveillance, where surveillance tests and intervals are tailored to individual HCC risk.

Overall, N-acetylglycine, amino acids, bile acids and choline-derived metabolites were identified as biomarkers to better identity patients with cirrhosis at high risk of HCC. Such biomarkers may significantly improve early-stage HCC detection for patients undergoing HCC surveillance, a critical step to increasing curative treatment opportunities and reducing mortality. This study also demonstrated the critical role of the gut microbiome in the progression of cirrhosis to HCC, in particular within the year prior to HCC development. Modulation of these microbiome-related metabolites by gut microbiome-targeted therapies is therefore an attractive potential strategy for reducing HCC risk.

## Abbreviations

AFP, alpha-fetoprotein; aOR, adjusted odds ratio; FDR, false discovery rate; GPC, glycerophosphorylcholine; HCC, hepatocellular carcinoma; LI-RADS, Liver Imaging Reporting and Data System; MASLD, metabolic dysfunction-associated steatotic liver disease; PNPLA3, patatin-like phospholipase domain-containing protein 3; SNP, single nucleotide polymorphism; TM6SF2, transmembrane 6 superfamily member 2.

## Financial support

This study was supported by National Institute of Health/National Cancer Institute R01 CA195524 to L.B.

## Conflict of interest

Dr. John Vierling is a scientific advisor to Bristol Myers Squibb, Merck, Novartis, LabCorp and Genentech.

Please refer to the accompanying ICMJE disclosure forms for further details.

## Authors’ contributions

Conceptualization, LB; Methodology, SYK, TLC, JMV, DWV, LB; Formal Analysis, JIS, ACF, SYK, CIS, PW; Investigation, JIS, CIS, TLC; Resources, JL, AE, DWC, JMV, DWV; Writing-Original Draft, JIS, ACF, SYK, LB; Visualization, CIS, ACF, SYK; Supervision, LB; Project Administration, TLC; Funding Acquisition, L.B.

## Data availability statement

The metabolomics data have been uploaded to the MetaboLights database (https://www.ebi.ac.uk/metabolights), under study accession number MTBLS8764.
