# Supplementary material for: Metabolomics biomarkers of hepatocellular carcinoma in a prospective cohort of patients with cirrhosis
Source: JHEP Rep. 2024 May 15;6(8):101119. doi: 10.1016/j.jhepr.2024.101119 (PMC11321296; doi:10.1016/j.jhepr.2024.101119)
Supplement: Multimedia component 1 [file mmc1.pdf]

Metabolomics biomarkers of hepatocellular carcinoma in a prospective cohort of patients with cirrhosis

Jessica I. Sanchez, Antoine C. Fontillas, Suet-Ying Kwan, Caren I. Sanchez, Tiffany L. Calderone, Jana L. Lee, Ahmed Elsaiey, Darrel W. Cleere, Peng Wei, John M. Vierling, David W. Victor, Laura Beretta

Table of contents

Supplementary materials and methods……………………………………………………………2

Supplementary figures………………………………………………………………………………8

Supplementary tables……………………………………………………………………………16

Supplementary results…………………………………………………………………………...29

Supplementary materials and methods

Patients Cohort

Medical history was collected to confirm eligibility (above 18 years of age, confirmed diagnosis of cirrhosis, no clinical evidence of significant hepatic decompensation). Cirrhosis was diagnosed using composite clinical, biochemical, hematological, imaging or histological criteria. Date of birth, gender, race, ethnicity, liver cirrhosis etiology, ascites status, encephalopathy status, Child Pugh score, Child Pugh class, Model for end stage liver disease (MELD) score, year of cirrhosis diagnosis, diabetes and date of diabetes onset were collected. CT was performed instead of MRI for 11 patients and 17 additional patients received a CT scan at one of the visits while receiving MRI for the remaining visits. Five of the 37 patients who developed HCC, presented with multifocal disease with the largest lesion ranging from 2.0 to 5.3cm (median=2.1cm). For the remaining 32 patients, the large majority presented with a small single lesion (median=2.1cm), with only 2 patients with a lesion larger than 4.0cm. Among the seven patients followed after treatment, four received transarterial chemoembolization alone, one received atezolizumab plus bevacizumab, one patient received transarterial chemoembolization followed by ablation and a liver transplant, and the last patient received Y90 radioembolization followed by a liver transplant. The median time of sample collection after treatment was 6.2 months.

Collected blood samples were kept at 4°C until processed, within 4 hours of collection. Serum, plasma and buffy coat aliquots were then stored in -80°C. The following clinical data were also collected at baseline and at each follow-up visit using electronic medical records: alpha-fetoprotein (AFP), alanine aminotransferase (ALT), aspartate aminotransferase (AST), alkaline phosphatase (ALP), total protein, albumin, total bilirubin, bilirubin direct, platelets, body mass index (BMI). All clinical data were entered into the study data capture system as an integrated part of a secure website supported by the Data Management Services team at MD Anderson Cancer Center. The secure website also provides an interface

to upload the related de-identified image files and documentation to a secure dedicated server space.

Global Metabolomics Profiling

Samples were prepared using the automated MicroLab STAR® system (Hamilton Company). For quality control (QC) purposes, several recovery standards were added prior to the first step in the extraction. Proteins were precipitated with methanol under vigorous shaking for 2 minutes (Glen Mills GrnoGrinder 2000) followed by centrifugation. The resulting extract was divided into five fractions: two for analysis by two separate reverse phase (RP)/UPLC-MS/MS methods with positive ion mode electrospray ionization (ESI), one for analysis by RP/UPLC-MS/MS with negative ion mode ESI, one for analysis by HILIC/UPLC-MS/MS with negative ion mode ESI, and one sample was reserved for backup.

Samples were briefly placed on a TurboVap® (Zymark) to remove the organic solvent. The sample extracts were stored overnight under nitrogen before preparation for analysis. Several types of controls were analyzed with the experimental samples: a pooled matrix sample generated by taking a small volume of each experimental sample served as a technical replicate throughout the data set; extracted water samples served as process blanks; and a cocktail of QC standards. Instrument variability was determined by calculating the median relative standard deviation (RSD) for the standards that were added to each sample prior to injection into the mass spectrometers. Overall process variability was determined by calculating the median RSD for all endogenous metabolites present in 100% of the pooled matrix samples. Experimental samples were randomized across the platform run with QC samples spaced evenly among the injections. All methods utilized a Waters ACQUITY ultra-performance liquid chromatography (UPLC) and a Thermo Scientific Q-Exactive high resolution/accurate mass spectrometer interfaced with a heated electrospray ionization (HESI-II) source and Orbitrap mass analyzer operated at 35,000 mass resolution. The sample extract was dried then reconstituted in solvents compatible to each of the four methods. Each reconstitution solvent contained a series of standards at fixed concentrations to ensure injection and

chromatographic consistency. One aliquot was analyzed using acidic positive ion conditions, chromatographically optimized for more hydrophilic compounds. In this method, the extract was gradient eluted from a C18 column (Waters UPLC BEH C18-2.1x100 mm, 1.7 µm) using water and methanol, containing 0.05% perfluoropentanoic acid (PFPA) and 0.1% formic acid (FA). Another aliquot was also analyzed using acidic positive ion conditions, however it was chromatographically optimized for more hydrophobic compounds. In this method, the extract was gradient eluted from the same afore mentioned C18 column using methanol, acetonitrile, water, 0.05% PFPA and 0.01% FA and was operated at an overall higher organic content. Another aliquot was analyzed using basic negative ion optimized conditions using a separate dedicated C18 column. The basic extracts were gradient eluted from the column using methanol and water, however with 6.5mM Ammonium Bicarbonate at pH 8. The fourth aliquot was analyzed via negative ionization following elution from a HILIC column (Waters UPLC BEH Amide 2.1x150 mm, 1.7 µm) using a gradient consisting of water and acetonitrile with 10mM Ammonium Formate, pH 10.8. The MS analysis alternated between MS and data-dependent MS^n scans using dynamic exclusion. The scan range varied slighted between methods but covered 70-1000 m/z. Raw data was extracted, peak-identified and QC processed using Metabolon's hardware and software. Compounds were identified by comparison to library entries of purified standards or recurrent unknown entities.

Statistical analyses

Table S1: Two-tailed t-test for continuous variables and Fisher test for categorical variables were used to compare demographic and clinical parameters between patients enrolled at the two sites as well as between patients who developed HCC during follow-up (Cases) and those who didn't (Controls).

Tables S2 and S3, Figures 1A and S5, Supplementary Figure and Table: To determine the association between circulating metabolite levels and HCC outcome (at any time point; within 6 months, 12 months or 24 months prior to HCC diagnosis) or HCC treatment, linear mixed-effects modeling using Maximum

Likelihood Estimation was performed, using the “lmer” function of the “lme4” R package. Grouping based on binary clinical outcome (Cases versus Controls for HCC development vs no development; Cases-T vs Cases for HCC patients post- versus pre-treatment), time (in months to latest visit) and the time × group interaction term were modeled as fixed effects, whereas patient ID was modeled as a random effect to account for repeated measures from the same patient. Log-transformed metabolite levels were used as the outcome variables. For each metabolite, the model was represented by [$y = \beta_0 + \beta_1 x_1 + \beta_2 x_2 + \beta_3 x_1 x_2 + \mu + \epsilon$], where y is the log-transformed metabolite abundance, β_0 is the global intercept, β_1 is the coefficient for time (x_1), β_2 is the coefficient for group (x_2) (binary clinical outcome, with Controls=0 and Cases=1; or Cases=0 and Cases-T=1), β_3 is the coefficient for time × group interaction (i.e. difference between cases vs controls), μ is the patient-specific random effect, and ϵ *is the residual error.* The *p*-value of each fixed effect was calculated using normal approximation and was also corrected for multiple testing by the Benjamini-Hochberg method, giving *q*-values. The coefficient, *p*-value and *q*-value for the fixed effect “group” were used for subsequent volcano plots.

Figures S2 and 1B: To identify highly correlated clusters of HCC-associated metabolites, Spearman’s correlation was performed between the 150 metabolites associated with HCC.

Figure 1C: Logistic regression was performed to determine the accuracy of each metabolite in predicting HCC development after adjusting for clinical covariates. Using the “glm.fit” function, we obtained odds ratios adjusted for age, gender and diabetes (AOR) and 95% confidence intervals for each unit increase in the level of metabolite (normalized, imputed, log-transformed values).

Figures S3, S6, 2A and 3A: To determine the predictive ability of a selected combination of HCC-related metabolites, the conditional inference random forest machine learning algorithm was implemented, using the “cforest” function of the “party” package, combined with the “caret” package. Demographic and genetic variables (age, gender, PNPLA3 rs738409 GG genotype, TM6SF2 rs58542926 CT/TT genotype), AFP and the HCC-associated metabolites were used as independent variables, while HCC diagnosis was

used as the binary outcome. Using the “train” function of the “caret” package, the optimal “mtry” value giving the maximum AUC was determined by 5-fold cross validation. Conditional importance scores (Mean Decrease in Accuracy) at the optimal “mtry” were generated to obtain final rankings of importance.

Figures 2B-C and 3B-C: To further select for top variables, recursive feature elimination was implemented using the “rfe” function of the “caret” package and incorporating resampling through 3-fold cross validation. The number of variables giving optimum model performance by the “accuracy” metric was determined. Receiver operating characteristic (ROC) curve analysis was performed to determined the predictive accuracy of a minimal panel, consisting of the 6 predictors with the highest importance by recursive feature elimination. Logistic regression models were fit using the “glm” function in R; the resulting fitted probabilities were used for graphing ROC curves and computing the area under the curve (AUC) using the pROC and ROCR packages.

Table S4, Figure 4: To determine the effect of demographic, clinical or genetic variables on HCC-associated metabolomic profiles, redundancy analysis (RDA) was performed using the “capscale” function in the Vegan package for R. Normalized, imputed, log-transformed values of the HCC-associated metabolites were used as the response variables, while age, gender, etiologies, PNPLA3 rs738409 (in increasing number of the risk allele G) and TM6SF2 rs58542926 (in increasing number of the risk allele T), were used as the explanatory variables. To identify the specific metabolites affected by gender, TM6SF2 rs58542926 and PNPLA3 rs738409, linear mixed-effects modeling was again performed. For each of the three clinical variables, the model was represented by [$y = \beta_0 + \beta_1 x_1 + \mu + \epsilon$], where y is the log-transformed metabolite abundance, β_0 is the global intercept, β_1 is the coefficient for the clinical variable (x_1) (gender: female=0, male=1; TM6SF2 rs58542926: CC=0, CT=1, TT=2; PNPLA3 rs738409: CC=0, GG=1), μ is the patient-specific random effect, and ϵ is the residual error. For each model, the coefficient, *p*-value and Benjamini-Hochberg-adjusted *q*-values of the clinical variable was generated.

Table S5, Figure 5: To determine whether HCC-associated metabolites could distinguish between patients with LI-RADS-3 lesions that developed HCC (Cases-LR3) and those that did not (Controls-LR3), linear mixed-effects modeling was again performed using only Controls and Cases with LI-RADS-3 lesions. Log-transformed metabolite levels were used as the outcome variable. For each metabolite, the model was represented by [$y = \beta_0 + \beta_1 x_1 + \mu + \epsilon$], where y is the log-transformed metabolite abundance, β_0 is the global intercept, β_1 is the coefficient for group (x_1) (binary clinical outcome, with Controls=0 and Cases=1), μ is the patient-specific random effect, and ϵ is the residual error. The *p*-value of each fixed effect was calculated using normal approximation and was also corrected for multiple testing by the Benjamini-Hochberg method, giving *q*-values. The coefficient, *p*-value and *q*-value for the fixed effect "group" were used for subsequent volcano plots. Using the 23 significant metabolites from linear mixed-effects modeling, principal component analysis (PCA) was performed using the "cmdscale" function, and Euclidean distances based on log-transformed metabolite levels. Beta dispersion and permutational multivariate analysis of variance (PERMANOVA) tests were performed with the Vegan package. Ellipses were drawn using the standard deviation of point scores.

Supplementary figures

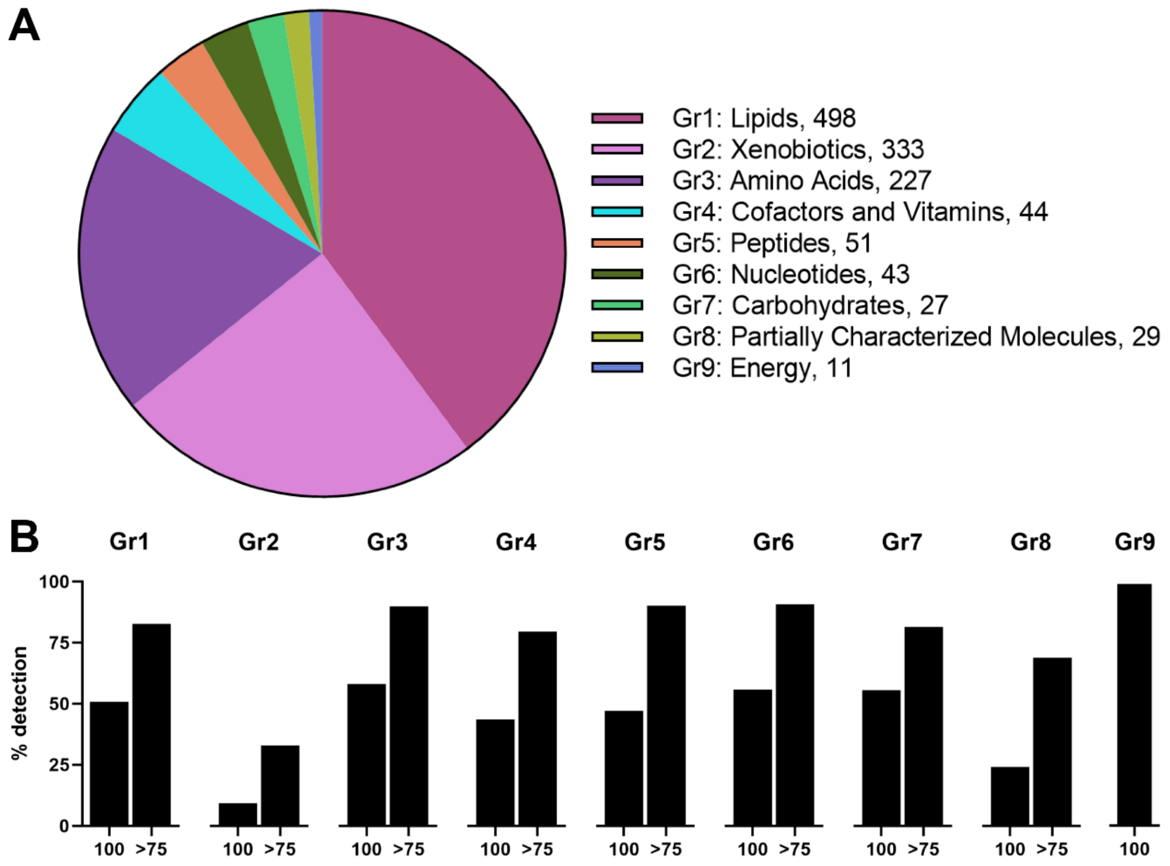

Fig. S1. Overall distribution of the metabolites measured in the study. (A) Pie chart displaying the distribution among different pathways, of the metabolites measured in the 612 serum samples. **(B)** Column graphs showing for each pathway (Gr1 to Gr9), the percentage of metabolites detected in 100% of the samples or in over 75% of the samples. The majority (58.1%) of the metabolites in the Amino Acids super-pathway were detected in all 612 serum samples, with 89.9% of them detected in at least 75% of the samples. Similarly, the majority of the Lipids-related metabolites (50.8%), of the Carbohydrates-related metabolites (55.5%) and of the Nucleotides-related metabolites (55.8%), were detected in all samples, with 82.7%, 81.5% and 90.7% of them detected in at least 75% of the samples, respectively. The 11 energy-related metabolites were also detected in the large majority of samples (at least 99% of the samples). More variation was observed for the detection of the Cofactors and Vitamins-related metabolites (43.5% detected in all samples, 79.5% detected in at least 75% of the samples) and of the Peptides-related

metabolites (47.1% detected in all samples, 90.2% detected in at least 75% of the samples). The largest variations were observed as anticipated for the Partially Characterized Molecules, with 24.1% of them detected in all samples and 68.9% in at least 75% of the samples, as well as for Xenobiotics with only 9.3% detected in all samples and 33.0% detected in at least 75% of the samples. A large number of xenobiotics (33.6%) were detected in less than 5% of the samples.

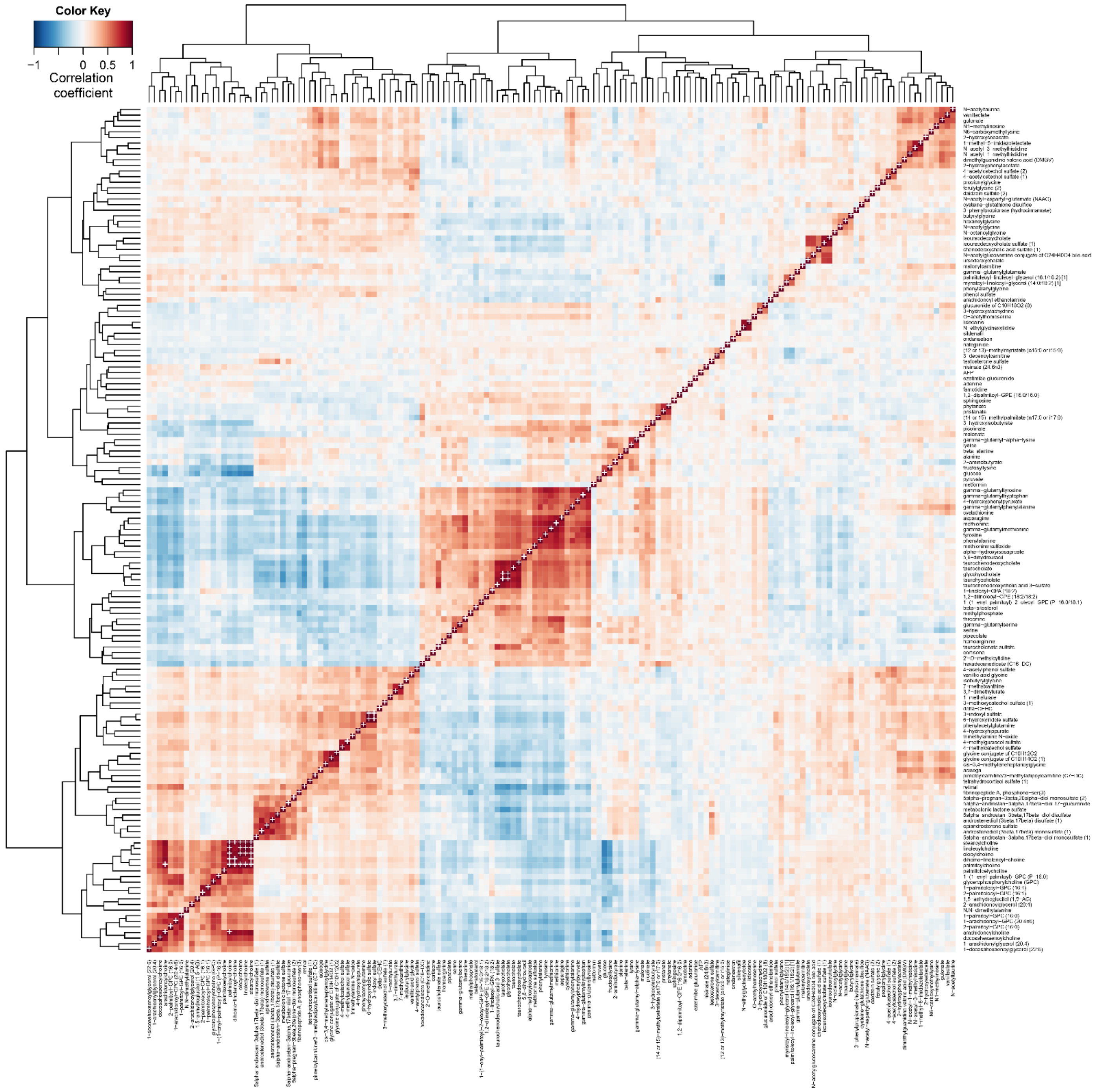

Fig. S2. Correlation matrix of the 151 differential metabolites between cases and controls.

Metabolites with a Spearman's correlation coefficient of r>0.9 and p<0.05 are indicated with a white cross.

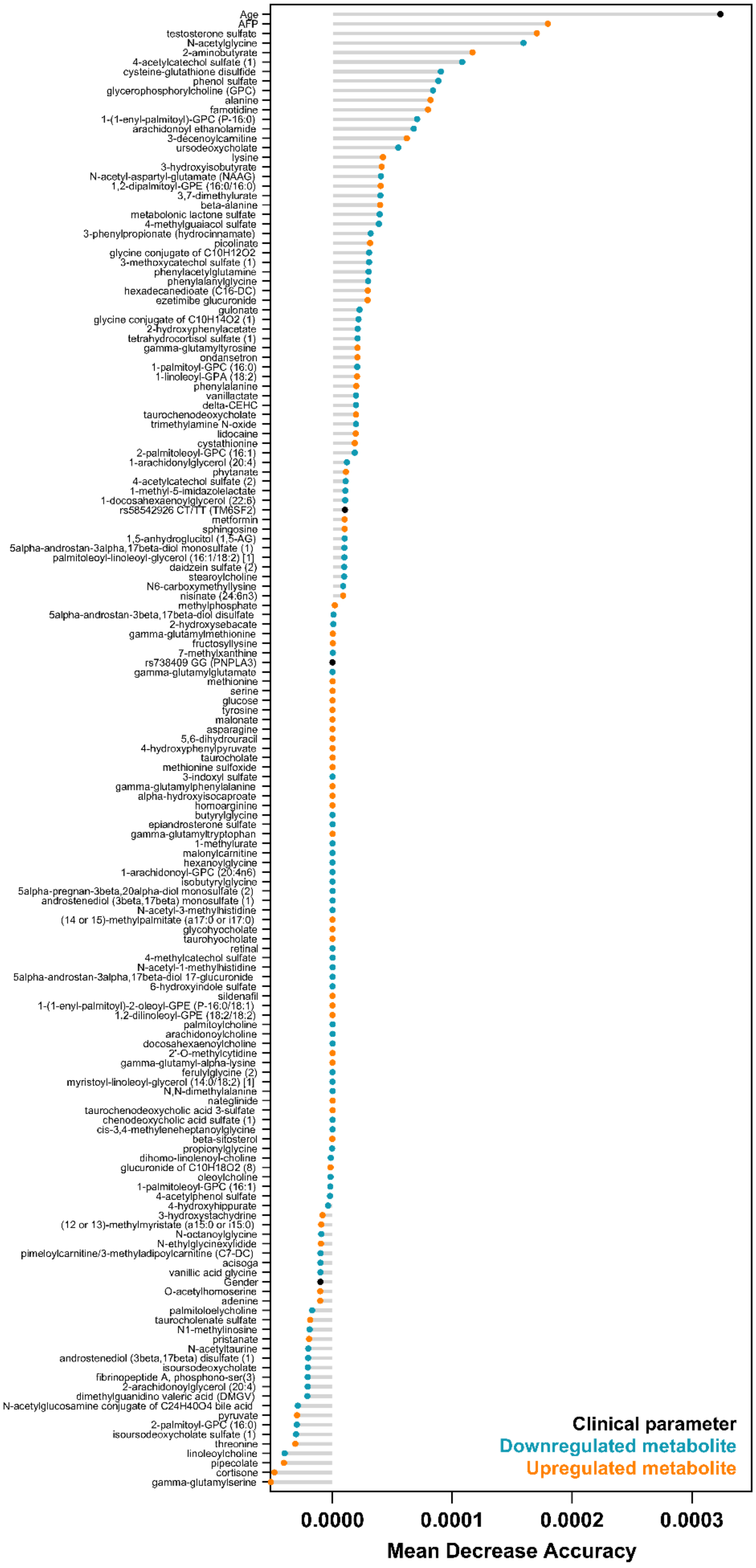
Age
AFP
testosterone sulfate
N-acetylglycine
2-aminobutyrate
4-acetylcatechol sulfate (1)
cysteine-glutathione disulfide
phenol sulfate
glycerophosphorylcholine (GPC)
alanine
famotidine
1-(1-enyl-palmitoyl)-GPC (P-16:0)
arachidonoyl ethanolamide
3-decenoylcarnitine
ursodeoxycholate
lysine
3-hydroxyisobutyrate
N-acetyl-aspartyl-glutamate (NAAG)
1,2-dipalmitoyl-GPE (16:0/16:0)
3,7-dimethylurate
beta-alanine
metabolonic lactone sulfate
4-methylguaiacol sulfate
3-phenylpropionate (hydrocinnamate)
picolinate
glycine conjugate of C10H12O2
3-methoxycatechol sulfate (1)
phenylacetylglutamine
phenylalanylglycine
hexadecanedioate (C16-DC)
ezetimibe glucuronide
gulonate
glycine conjugate of C10H14O2 (1)
2-hydroxyphenylacetate
tetrahydrocortisol sulfate (1)
gamma-glutamyltyrosine
ondansetron
1-palmitoyl-GPC (16:0)
1-linoleoyl-GPA (18:2)
phenylalanine
vanillactate
delta-CEHC
taurochenodeoxycholate
trimethylamine N-oxide
lidocaine
cystathionine
2-palmitoleoyl-GPC (16:1)
1-arachidonylglycerol (20:4)
phytanate
4-acetylcatechol sulfate (2)
1-methyl-5-imidazolelactate
1-docosahexaenoylglycerol (22:6)
rs58542926 CT/TT (TM6SF2)
metformin
sphingosine
1,5-anhydroglucitol (1,5-AG)
5alpha-androstan-3alpha,17beta-diol monosulfate (1)
palmitoleoyl-linoleoyl-glycerol (16:1/18:2) [1]
daidzein sulfate (2)
stearoylcholine
N6-carboxymethyllysine
nisinate (24:6n3)
methylphosphate
5alpha-androstan-3beta,17beta-diol disulfate
2-hydroxysebacate
gamma-glutamylmethionine
fructosyllysine
7-methylxanthine
rs738409 GG (PNPLA3)
gamma-glutamylglutamate
methionine
serine
glucose
tyrosine
malonate
asparagine
5,6-dihydrouracil
4-hydroxyphenylpyruvate
taurocholate
methionine sulfoxide
3-indoxyl sulfate
gamma-glutamylphenylalanine
alpha-hydroxyisocaproate
homoarginine
butyrylglycine
epiandrosterone sulfate
gamma-glutamyltryptophan
1-methylurate
malonylcarnitine
hexanoylglycine
1-arachidonoyl-GPC (20:4n6)
isobutyrylglycine
5alpha-pregnan-3beta,20alpha-diol monosulfate (2)
androstenediol (3beta,17beta) monosulfate (1)
N-acetyl-3-methylhistidine
(14 or 15)-methylpalmitate (a17:0 or i17:0)
glycohyocholate
taurohyocholate
retinal
4-methylcatechol sulfate
N-acetyl-1-methylhistidine
5alpha-androstan-3alpha,17beta-diol 17-glucuronide
6-hydroxyindole sulfate
sildenafil
1-(1-enyl-palmitoyl)-2-oleoyl-GPE (P-16:0/18:1)
1,2-dilinoleoyl-GPE (18:2/18:2)
palmitoylcholine
arachidonoylcholine
docosahexaenoylcholine
2'-O-methylcytidine
gamma-glutamyl-alpha-lysine
feruloylglycine (2)
myristoyl-linoleoyl-glycerol (14:0/18:2) [1]
N,N-dimethylalanine
nateglinide
taurochenodeoxycholic acid 3-sulfate
chenodeoxycholic acid sulfate (1)
cis-3,4-methyleneheptanoylglycine
beta-sitosterol
propionylglycine
dihomo-linolenoyl-choline
glucuronide of C10H18O2 (8)
oleoylcholine
1-palmitoleoyl-GPC (16:1)
4-acetylphenol sulfate
4-hydroxyhippurate
3-hydroxystachydrine
(12 or 13)-methylmyristate (a15:0 or i15:0)
N-octanoylglycine
N-ethylglycinexylidide
pimeloylcarnitine/3-methyladipoylcarnitine (C7-DC)
acisoga
vanillic acid glycine
Gender
O-acetylhomoserine
adenine
palmitoloelycholine
taurocholenate sulfate
N1-methylinosine
pristanate
N-acetyltaurine
androstenediol (3beta,17beta) disulfate (1)
isoursodeoxycholate
fibrinopeptide A, phospho-ser(3)
2-arachidonoylglycerol (20:4)
dimethylguanidino valeric acid (DMGV)
N-acetylglucosamine conjugate of C24H40O4 bile acid
pyruvate
2-palmitoyl-GPC (16:0)
isoursodeoxycholate sulfate (1)
threonine
linoleoylcholine
pipecolate
cortisone
gamma-glutamylserine
Clinical parameter
Downregulated metabolite
Upregulated metabolite
0.0000
0.0001
0.0002
0.0003
Mean Decrease Accuracy

Fig. S3. Importance of individual clinical parameters and metabolites in predicting HCC diagnosis, as determined by conditional inference random forest. The conditional inference random forest machine learning algorithm was implemented to determine the contribution of each clinical parameter and metabolite to the prediction of HCC diagnosis. The importance of each variable was determined by the permutation-based mean decrease accuracy, representing the loss in model performance when each variable is excluded. Variables are sorted by descending importance.

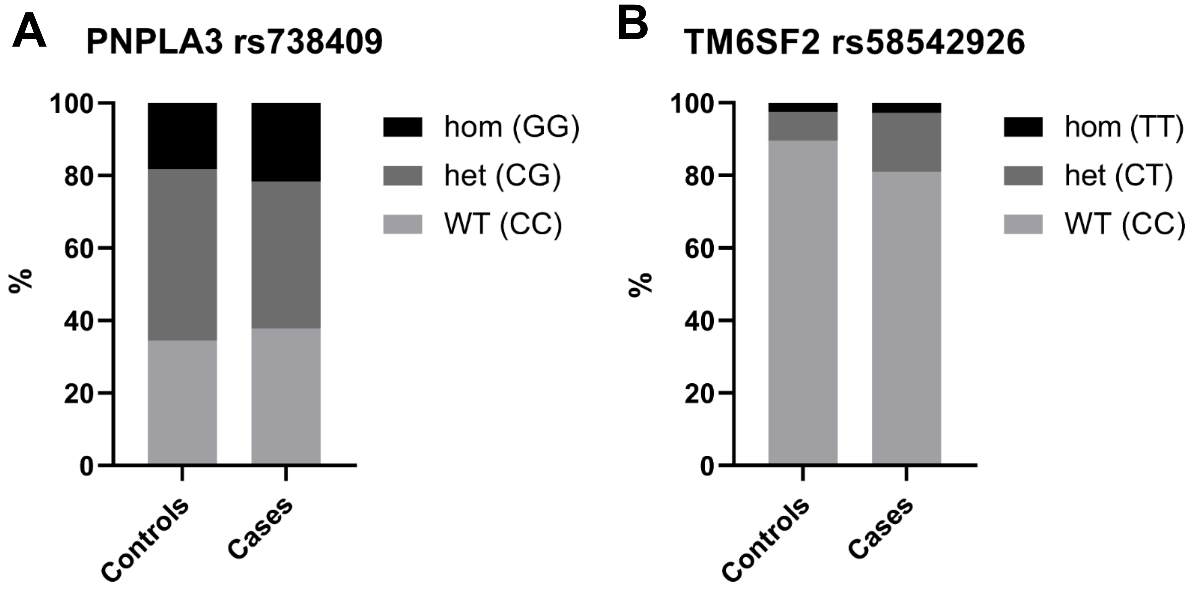

Fig. S4. PNPLA3 rs738409 and TM6SF2 rs58542926 genotyping in study participants. Genotype frequencies of **(A)** PNPLA3 rs738409, and **(B)** TM6SF2 rs58542926 in the 203 patients included in this study. WT: wild-type; het: heterozygous; hom: homozygous.

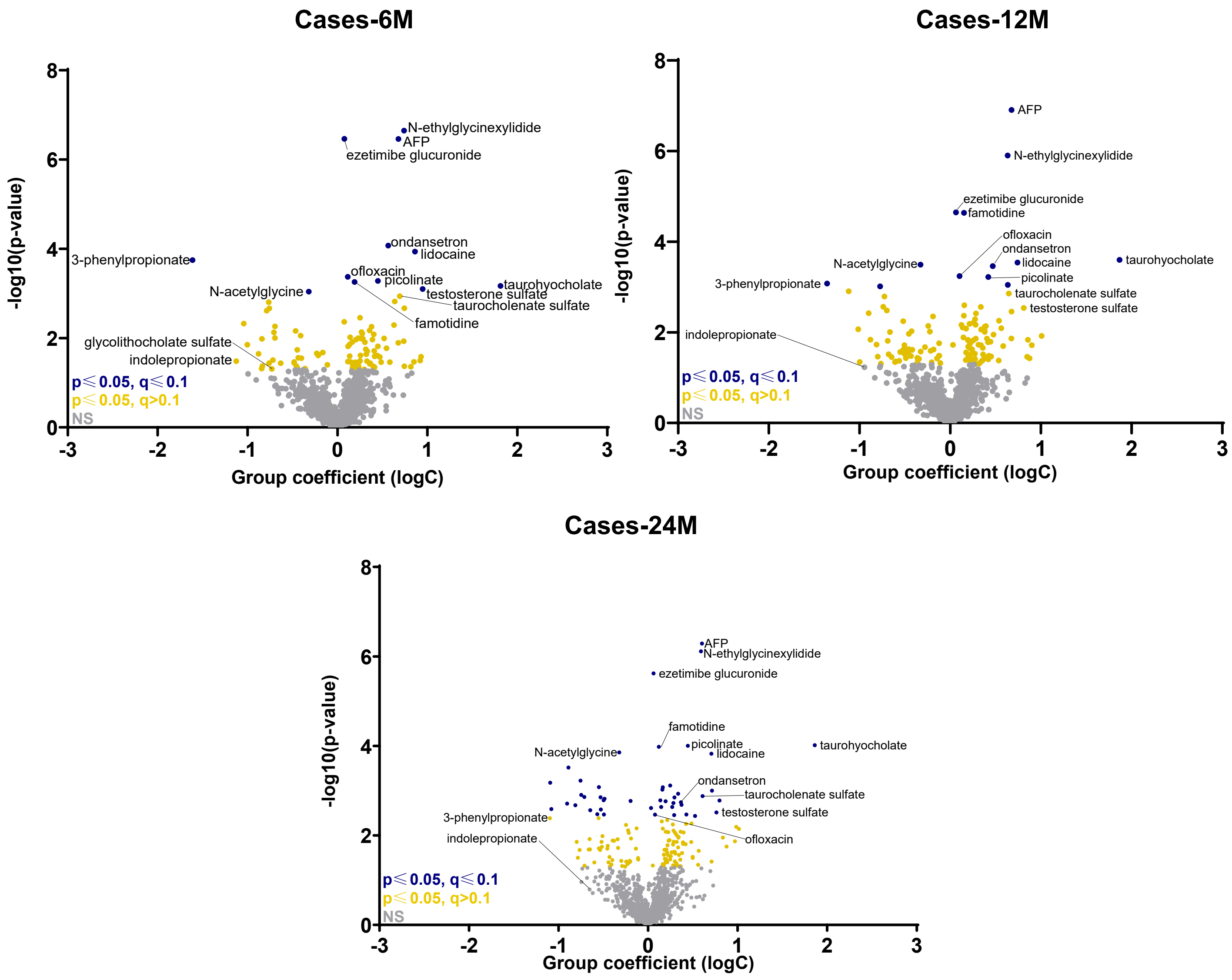

Fig. S5. Metabolite abundance changes in Cases within 6, 12 or 24 months prior to HCC diagnosis compared to Controls. Volcano plots for differential metabolites, between Controls and Cases-6M, Cases-12M or Cases-24M. The significance and coefficient of group (Cases within 6, 12, or 24 months versus Controls) as a fixed effect on metabolite abundance was determined by linear mixed-effects modeling. Group coefficients for log-transformed metabolite abundance (x-axis) and minus log10 p-values (y-axis) are shown. Metabolites that remained significant ($p \leq 0.05$, $q \leq 0.1$) after adjustment with the Benjamini–Hochberg method are shown in blue. Genes that did not remain significant ($p \leq 0.05$, $q > 0.1$) after adjustment with the Benjamini-Hochberg method are shown in yellow.

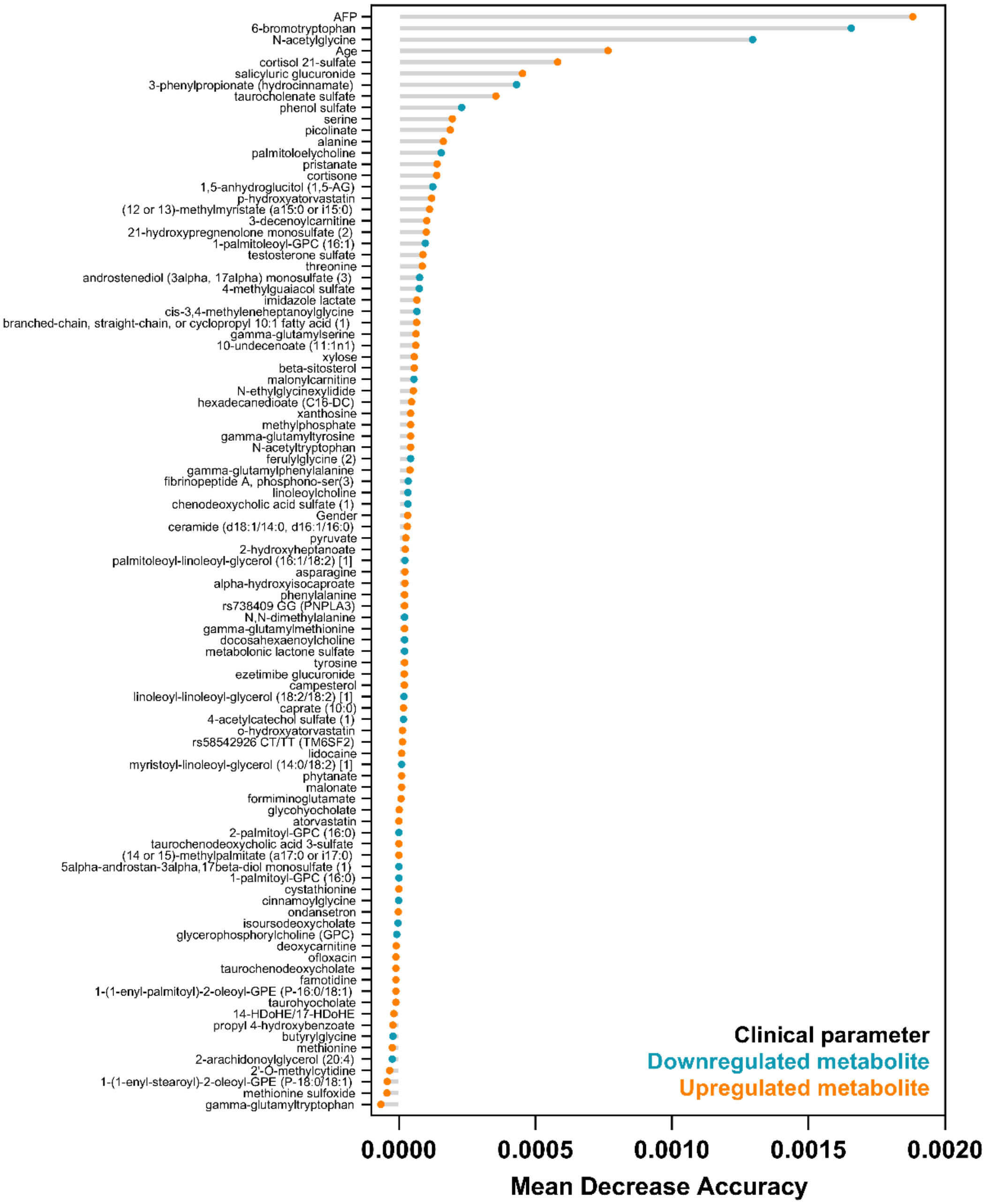

Fig. S6. Importance of individual clinical parameters and metabolites in predicting HCC diagnosis within 12 months, as determined by conditional inference random forest. The conditional inference random forest machine learning algorithm was implemented to determine the contribution of each clinical parameter and metabolite to the prediction of HCC diagnosis within 12 months of follow-up. The importance of each variable was determined by the permutation-based mean decrease accuracy, representing the loss in model performance when each variable is excluded. Variables are sorted by descending importance.

Supplementary tables

Table S1: Demographic and clinical parameters for 203 cirrhotic patients included in the study. Data are displayed as n (%) or mean (range) - median. AFP: alpha-fetoprotein; ALP: alkaline phosphatase; ALT: alanine aminotransferase; AST: aspartate aminotransferase; MELD score: model for end stage liver disease; NASH/NAFLD: nonalcoholic steatohepatitis/nonalcoholic fatty liver disease. P-values between the two sites and between Cases and Controls are shown.

Demographics	ALL (n=203)	Site 1 (n=108)	Site 2 (n=95)	p values	Controls (n=165)	Cases (n=38)	p values
**Gender (male)**	102 (50%)	64 (59%)	38 (40%)	0.231	81 (49%)	21 (55%)	0.590
**Race**				0.071			0.801
White	178 (88%)	100 (93%)	78 (82%)		146 (88%)	32 (84%)	
Black	17 (8%)	5 (5%)	12 (13%)		13 (8%)	4 (11%)	
Other	8 (4%)	3 (3%)	5 (5%)		6 (4%)	2 (5%)	
**Ethnicity**				0.324			0.827
Hispanic	41 (20%)	19 (18%)	22 (23%)		33 (20%)	8 (21%)	
Non-Hispanic	162 (80%)	89 (82%)	73 (77%)		132 (80%)	30 (79%)	
**Etiology**				0.01			0.439
HCV	62 (31%)	28 (26%)	34 (36%)		49 (30%)	13 (34%)	
HBV	14 (7%)	6 (6%)	8 (8%)		13 (8%)	1 (3%)	
NASH/NAFLD	77 (38%)	34 (31%)	43 (45%)		59 (36%)	18 (47%)	
Alcohol	52 (26%)	38 (35%)	14 (15%)		42 (25%)	10 (26%)	
Other	29 (14%)	13 (12%)	16 (17%)		26 (16%)	3 (8%)	
**Ascites**				<0.001			0.633
Absent	80 (39%)	20 (19%)	60 (63%)		63 (38%)	17 (45%)	
Controlled medically	113 (56%)	79 (73%)	34 (36%)		93 (56%)	20 (53%)	
Poorly controlled	10 (5%)	9 (8%)	1 (1%)		9 (5%)	1 (3%)	
**Encephalopathy**				<0.001			0.173
Absent	95 (47%)	28 (26%)	67 (71%)		81 (49%)	14 (37%)	
Controlled medically	108 (53%)	80 (74%)	28 (29%)		84 (51%)	24 (63%)	
**Child Pugh Class**				<0.001			0.632
Child Class A	81 (40%)	16 (15%)	65 (68%)		68 (41%)	13 (34%)	
Child Class B	98 (48%)	71 (66%)	27 (28%)		77 (47%)	21 (55%)	
Child Class C	24 (12%)	21 (19%)	3 (3%)		20 (12%)	4 (11%)	
**Child Pugh Score**	7 (5-11) - 7	8 (5-11) - 8	6 (5-10) - 6	<0.001	7.1 (5-11) -7	7.4 (5-11) -8	0.133
**Diabetes**	91 (45%)	51 (47%)	40 (42%)	0.483	67 (41%)	24 (63%)	0.018
**Age**	61 (24-83) -62	61 (24-78) -63	60 (24-83) -61	0.753	60 (24-83) -61	64 (48-79) -66	0.010
**Body Mass Index**	30.8 (18.5-49.5) -29.4	30.8 (18.9-45.1) -29.1	30.9 (18.5-49.5) -30.2	0.835	31.1 (18.5-48.6) - 29.9	29.6 (20.6-49.5) -28.3	0.189
**MELD Score**	11 (6-20) -11	13 (7-20) -13	9 (6-20) -8	<0.001	10.9 (6-20) -10	11.9 (6-20) -12	0.133
**Clinical Labs**							
**Protein (g/dL)**	7.3 (3.7-9.9) -7.4	7.2 (3.0-9.9) -7.2	7.3 (5.2-8.5) -7.4	0.547	7.3 (3.7-9.3) -7.4	7.2 (5.5-9.9) -7.3	0.589
**ALB (g/dL)**	3.6 (1.2-5.0) -3.6	3.3 (1.2-4.8) -3.3	3.9 (2.5-5.0) -4.0	<0.001	3.6 (1.2-5) -3.6	3.4 (1.8-4.6) -3.4	0.040
**Total Bilirubin (mg/dL)**	1.5 (0.2-7.7) -1.1	1.8 (0.2-5.9) -1.4	1.3 (0.3-7.7) -1.0	0.004	1.5 (0.2-5.7) -1.1	1.9 (0.3-7.7) -1.5	0.035

**Bilirubin Direct (mg/dL)**	0.6 (0.1-4.1) -0.4	0.6 (0.2-2.8) -0.4	0.5 (0.1-4.1) -0.3	0.666	0.5 (0.1-4.1) -0.4	0.7 (0.1-3.7) -0.5	0.053
**ALK (U/L)**	125.2 (11-911) -104	137 (54-911) -111	112 (11-413) -99	0.046	122.2 (11-911) -99	137.9 (61-273) -133.5	0.321
**AST (U/L)**	43.1 (14-225) -37	46 (14-225) -42	40 (16-223) -33	0.095	41.3 (14-223) -36	51.1 (19-225) -46	0.045
**ALT (U/L)**	33.3 (8-236) -26	34 (8-236) -26	33 (10-131) -26	0.831	32.2 (8-131) -26	37.8 (10-236) -28	0.199
**AFP (ng/mL)**	53.2 (0.9-5848) -3.8	6.7 (0.9-99.7) -3.7	106.4 (1.4-5848) - 4.1	0.118	4.4 (0.9-43) -3.7	271.7 (1.4-5848) -4.2	0.001
**Platelets (billion/L)**	118 (5.5-334) -104	114 (5.5-334) -97.5	123 (33-330) -113	0.272	119.7 (5.5-334) -107.5	108.9 (44-245) -98	0.323

Table S2: Abundance changes in metabolites between controls and cases. Significance of metabolites abundance between cases and controls was determined by linear mixed model. Super-pathway groups for metabolites are abbreviated as follows: Gr1: Lipids, Gr2: Xenobiotics, Gr3: Amino Acids: Gr4: Cofactors and Vitamins, Gr5: Peptides, Gr6: Nucleotides, Gr7: Carbohydrates, Gr8: Partially Characterized Molecules. Controls (%) and Cases (%) reflect the percentage of detection of each metabolite in controls and cases samples, respectively. AFP: Alpha-fetoprotein, GPE: Glycerophosphorylethanolamine, GPC: Glycerophosphorylcholine, GPA: Glycerophosphatidic acid, CEHC: carboxyethyl-hydroxychroman, C: group coefficient (log-transformed). The metabolites are ordered from highest to lowest group coefficient (C).

Metabolite	Group	Pathway	Controls (%)	Cases (%)	p	q	C
Taurohyocholate	Gr1	Secondary Bile Acid Metabolism	74.9	87.5	<0.001	0.041	1.53
Taurocholate	Gr1	Primary Bile Acid Metabolism	99.6	100	0.005	0.152	0.92
Metformin	Gr2	Drug - Metabolic	12.4	26.1	0.049	0.406	0.89
Taurochenodeoxycholate	Gr1	Primary Bile Acid Metabolism	100	100	0.01	0.242	0.82
3-hydroxystachydrine	Gr2	Food Component/Plant	68.6	80.7	0.05	0.408	0.80
Taurochenodeoxycholic acid 3-sulfate	Gr1	Secondary Bile Acid Metabolism	98.0	95.5	0.012	0.242	0.78
Glycohyocholate	Gr1	Secondary Bile Acid Metabolism	98.2	98.9	0.013	0.247	0.70
Pristanate	Gr1	Fatty Acid, Branched	56.1	68.2	0.002	0.107	0.67
Beta-sitosterol	Gr1	Sterol	65.9	71.6	0.005	0.149	0.52
Testosterone sulfate	Gr1	Androgenic Steroids	30.2	53.4	0.019	0.285	0.52
Glucuronide of C10H18O2 (8)	Gr8	Partially Characterized Molecules	70.4	86.4	0.04	0.379	0.48
AFP			100	100	<0.001	0.004	0.48
Taurocholenate sulfate	Gr1	Secondary Bile Acid Metabolism	100	100	0.005	0.152	0.45
Lidocaine	Gr2	Drug - Analgesics, Anesthetics	7.1	14.8	0.005	0.148	0.45
(12 or 13)-methylmyristate (a15:0 or i15:0)	Gr1	Fatty Acid, Branched	43.5	51.1	0.008	0.211	0.45
3-decenoylcarnitine	Gr1	Fatty Acid Metabolism (Acyl Carnitine, Monounsaturated)	52.0	62.5	0.006	0.152	0.43
O-acetylhomoserine	Gr3	Glycine, Serine and Threonine Metabolism	77.6	73.9	0.016	0.259	0.41
5,6-dihydrouracil	Gr6	Pyrimidine Metabolism, Uracil containing	91.0	96.6	0.018	0.28	0.40
1,2-dilinoleoyl-GPE (18:2/18:2)	Gr1	Phosphatidylethanolamine (PE)	87.6	89.8	0.033	0.354	0.36
1,2-dipalmitoyl-GPE (16:0/16:0)	Gr1	Phosphatidylethanolamine (PE)	58.0	64.8	<0.001	0.041	0.36
N-ethylglycinexylidide	Gr2	Drug - Analgesics, Anesthetics	5.1	12.5	0.001	0.068	0.35
Picolinate	Gr3	Tryptophan Metabolism	99.8	100	<0.001	0.048	0.35

Hexadecanedioate (C16-DC)	Gr1	Fatty Acid, Dicarboxylate	99.2	98.9	0.015	0.253	0.32
Phytanate	Gr2	Food Component/Plant	99.6	100	0.018	0.28	0.31
Cystathionine	Gr3	Methionine, Cysteine, SAM and Taurine Metabolism	99.4	100	0.048	0.406	0.29
Nisinate (24:6n3)	Gr1	Long Chain Polyunsaturated Fatty Acid (n3 and n6)	28.6	36.4	0.038	0.371	0.29
Malonate	Gr1	Fatty Acid Synthesis	98.8	98.9	0.014	0.253	0.28
Gamma-glutamylmethionine	Gr5	Gamma-glutamyl Amino Acid	100	100	0.001	0.079	0.28
Gamma-glutamyltyrosine	Gr5	Gamma-glutamyl Amino Acid	100	100	0.001	0.068	0.26
Pipecolate	Gr3	Lysine Metabolism	100	100	0.033	0.354	0.26
Cortisone	Gr1	Corticosteroids	98.8	100	0.013	0.247	0.26
Gamma-glutamyltryptophan	Gr5	Gamma-glutamyl Amino Acid	99.0	100	0.001	0.063	0.25
Alpha-hydroxyisocaproate	Gr3	Leucine, Isoleucine and Valine Metabolism	98.6	100	0.022	0.293	0.24
Ondansetron	Gr2	Drug - Gastrointestinal	3.1	8.0	0.02	0.287	0.24
Methionine sulfoxide	Gr3	Methionine, Cysteine, SAM and Taurine Metabolism	100	100	0.003	0.11	0.24
Methionine	Gr3	Methionine, Cysteine, SAM and Taurine Metabolism	100	100	<0.001	0.041	0.23
(14 or 15)-methylpalmitate (a17:0 or i17:0)	Gr1	Fatty Acid, Branched	98.4	98.9	0.042	0.386	0.23
Pyruvate	Gr7	Glycolysis, Gluconeogenesis, and Pyruvate Metabolism	100	100	0.012	0.242	0.22
3-hydroxyisobutyrate	Gr3	Leucine, Isoleucine and Valine Metabolism	98.4	100	0.025	0.31	0.22
1-linoleoyl-GPA (18:2)	Gr1	Lysophospholipid	99.8	100	0.016	0.262	0.21
Homoarginine	Gr3	Urea cycle; Arginine and Proline Metabolism	100	100	0.019	0.286	0.20
Sphingosine	Gr1	Sphingosines	94.3	98.9	0.012	0.242	0.20
4-hydroxyphenylpyruvate	Gr3	Tyrosine Metabolism	100	100	0.031	0.352	0.20
Tyrosine	Gr3	Tyrosine Metabolism	100	100	0.003	0.11	0.20
Methylphosphate	Gr6	Purine and Pyrimidine Metabolism	100	100	0.015	0.253	0.18
Fructosyllysine	Gr3	Lysine Metabolism	100	100	0.034	0.355	0.18
Beta-alanine	Gr6	Pyrimidine Metabolism, Uracil containing	99.8	100	0.012	0.242	0.17
Gamma-glutamylserine	Gr5	Gamma-glutamyl Amino Acid	99.6	100	0.002	0.107	0.16
2-aminobutyrate	Gr3	Glutathione Metabolism	100	100	0.015	0.253	0.16
Alanine	Gr3	Alanine and Aspartate Metabolism	100	100	<0.001	0.025	0.16
2'-O-methylcytidine	Gr6	Pyrimidine Metabolism, Cytidine containing	97.6	100	0.033	0.354	0.16
Gamma-glutamylphenylalanine	Gr5	Gamma-glutamyl Amino Acid	100	100	0.033	0.354	0.15
Serine	Gr3	Glycine, Serine and Threonine Metabolism	100	100	<0.001	0.051	0.14
Threonine	Gr3	Glycine, Serine and Threonine Metabolism	100	100	0.002	0.11	0.14
Adenine	Gr6	Purine Metabolism, Adenine containing	100	100	0.004	0.138	0.14
1-(1-enyl-palmitoyl)-2-oleoyl-GPE (P-16:0/18:1)	Gr1	Plasmalogen	99.6	98.9	0.047	0.401	0.13
Asparagine	Gr3	Alanine and Aspartate Metabolism	100	100	0.002	0.107	0.13

Glucose	Gr7	Glycolysis, Gluconeogenesis, and Pyruvate Metabolism	100	100	0.021	0.291	0.12
Phenylalanine	Gr3	Phenylalanine Metabolism	100	100	0.002	0.107	0.12
Gamma-glutamyl-alpha-lysine	Gr5	Gamma-glutamyl Amino Acid	100	100	0.029	0.335	0.11
Lysine	Gr3	Lysine Metabolism	100	100	0.025	0.31	0.07
Famotidine	Gr2	Drug - Gastrointestinal	1.2	5.7	0.011	0.242	0.07
Ezetimibe glucuronide	Gr2	Drug - Metabolic	0.0	9.1	<.001	0.009	0.05
Sildenafil	Gr2	Drug - Cardiovascular	0.2	2.3	0.043	0.389	0.04
Nateglinide	Gr2	Drug - Metabolic	0.2	1.1	0.008	0.211	0.02
1-palmitoyl-GPC (16:0)	Gr1	Lysophospholipid	100	100	0.034	0.354	-0.09
GPC	Gr1	Phospholipid Metabolism	100	100	0.015	0.253	-0.13
2-palmitoyl-GPC (16:0)	Gr1	Lysophospholipid	100	100	0.036	0.361	-0.15
1-(1-enyl-palmitoyl)-GPC (P-16:0)	Gr1	Lysoplasmalogen	100	100	0.021	0.291	-0.15
1-arachidonoyl-GPC (20:4n6)	Gr1	Lysophospholipid	100	100	0.049	0.406	-0.17
1-palmitoleoyl-GPC (16:1)	Gr1	Lysophospholipid	100	100	0.012	0.242	-0.18
N1-methylinosine	Gr6	Purine Metabolism, (Hypo)Xanthine/Inosine containing	99.6	96.6	0.034	0.355	-0.19
N-acetyl-aspartyl-glutamate	Gr3	Glutamate Metabolism	93.9	89.8	0.025	0.31	-0.19
Phenylalanylglycine	Gr5	Dipeptide	51.0	25.0	0.011	0.242	-0.20
N-acetyltaurine	Gr3	Methionine, Cysteine, SAM and Taurine Metabolism	99.0	98.9	0.044	0.392	-0.21
Gulonate	Gr4	Ascorbate and Aldarate Metabolism	96.7	94.3	0.049	0.408	-0.23
Vanillactate	Gr3	Tyrosine Metabolism	99.0	96.6	0.022	0.293	-0.24
Arachidonoyl ethanolamide	Gr1	Endocannabinoid	50.4	30.7	0.002	0.107	-0.24
2-hydroxysebacate	Gr1	Fatty Acid, Dicarboxylate	97.6	97.7	0.043	0.389	-0.24
Glycine conjugate of C10H14O2 (1)	Gr8	Partially Characterized Molecules	99.8	100	0.033	0.354	-0.25
2-palmitoleoyl-GPC (16:1)	Gr1	Lysophospholipid	99.4	98.9	0.01	0.235	-0.25
Isobutyrylglycine	Gr3	Leucine, Isoleucine and Valine Metabolism	60.6	48.9	0.036	0.364	-0.28
Gamma-glutamylglutamate	Gr5	Gamma-glutamyl Amino Acid	86.1	83.0	0.023	0.298	-0.29
1-arachidonylglycerol (20:4)	Gr1	Monoacylglycerol	99.6	98.9	0.018	0.28	-0.29
Retinal	Gr4	Vitamin A Metabolism	88.2	90.9	0.045	0.393	-0.29
Myristoyl-linoleoyl-glycerol (14:0/18:2) [1]	Gr1	Diacylglycerol	23.1	18.2	0.027	0.326	-0.29
N-acetylglycine	Gr3	Glycine, Serine and Threonine Metabolism	100	100	<0.001	0.022	-0.29
N-octanoylglycine	Gr1	Fatty Acid Metabolism (Acyl Glycine)	56.1	28.4	0.011	0.242	-0.30
Butyrylglycine	Gr1	Fatty Acid Metabolism (also BCAA Metabolism)	45.1	31.8	0.028	0.331	-0.30
Delta-CEHC	Gr4	Tocopherol Metabolism	92.7	84.1	0.026	0.312	-0.30
2-hydroxyphenylacetate	Gr3	Phenylalanine Metabolism	90.0	83.0	0.038	0.371	-0.30
Phenylacetylglutamine	Gr5	Acetylated Peptides	100	100	0.042	0.388	-0.31
Malonylcarnitine	Gr1	Fatty Acid Synthesis	68.6	60.2	0.007	0.192	-0.31
4-hydroxyhippurate	Gr2	Benzoate Metabolism	100	100	0.033	0.354	-0.32

N-acetyl-1-methylhistidine	Gr3	Histidine Metabolism	98.6	96.6	0.05	0.408	-0.34
Trimethylamine N-oxide	Gr1	Phospholipid Metabolism	100	100	0.024	0.31	-0.35
Hexanoylglycine	Gr1	Fatty Acid Metabolism (Acyl Glycine)	45.7	21.6	0.005	0.148	-0.35
Acisoga	Gr3	Polyamine Metabolism	94.3	88.6	0.008	0.211	-0.35
5alpha-androstan-3alpha,17beta-diol 17-glucuronide	Gr1	Androgenic Steroids	31.0	22.7	0.035	0.355	-0.36
N,N-dimethylalanine	Gr3	Alanine and Aspartate Metabolism	95.7	94.3	0.035	0.355	-0.36
Tetrahydrocortisol sulfate (1)	Gr1	Corticosteroids	66.7	56.8	0.045	0.393	-0.37
Propionylglycine	Gr1	Fatty Acid Metabolism (also BCAA Metabolism)	55.3	52.3	0.015	0.253	-0.37
Pimeloylcarnitine/3-methyladipoylcarnitine (C7-DC)	Gr1	Fatty Acid Metabolism (Acyl Carnitine, Dicarboxylate)	90.0	88.6	0.02	0.286	-0.37
Palmitoylcholine	Gr1	Fatty Acid Metabolism (Acyl Choline)	99.8	100	0.003	0.131	-0.38
3-indoxyl sulfate	Gr3	Tryptophan Metabolism	100	100	0.022	0.293	-0.38
Oleoylcholine	Gr1	Fatty Acid Metabolism (Acyl Choline)	99.4	95.5	0.003	0.131	-0.39
1-docosahexaenoylglycerol (22:6)	Gr1	Monoacylglycerol	63.3	48.9	0.041	0.384	-0.39
Linoleoylcholine	Gr1	Fatty Acid Metabolism (Acyl Choline)	100	97.7	0.005	0.148	-0.39
Ferulylglycine (2)	Gr2	Food Component/Plant	34.9	21.6	0.021	0.293	-0.39
Phenol sulfate	Gr3	Tyrosine Metabolism	100	100	0.045	0.393	-0.40
Stearoylcholine	Gr1	Fatty Acid Metabolism (Acyl Choline)	99.2	93.2	0.004	0.142	-0.40
1-methyl-5-imidazolelactate	Gr3	Histidine Metabolism	98.2	92.0	0.021	0.293	-0.42
5alpha-pregnan-3beta,20alpha-diol monosulfate (2)	Gr1	Progestin Steroids	79.2	70.5	0.027	0.326	-0.42
3,7-dimethylurate	Gr2	Xanthine Metabolism	73.3	54.5	0.033	0.354	-0.42
4-acetylcatechol sulfate (2)	Gr2	Food Component/Plant	44.7	27.3	0.031	0.352	-0.44
4-acetylphenol sulfate	Gr2	Benzoate Metabolism	90.4	85.2	0.014	0.253	-0.45
3-methoxycatechol sulfate (1)	Gr2	Benzoate Metabolism	99.2	98.9	0.046	0.398	-0.45
Palmitoloelycholine	Gr1	Fatty Acid Metabolism (Acyl Choline)	80.0	54.5	0.002	0.108	-0.46
glycine conjugate of C10H12O2	Gr8	Partially Characterized Molecules	80.8	70.5	0.004	0.138	-0.46
Epiandrosterone sulfate	Gr1	Androgenic Steroids	99.8	100	0.038	0.371	-0.47
1-methylurate	Gr2	Xanthine Metabolism	77.1	72.7	0.011	0.242	-0.47
Dihomo-linolenoyl-choline	Gr1	Fatty Acid Metabolism (Acyl Choline)	95.1	86.4	0.004	0.138	-0.47
N6-carboxymethyllysine	Gr7	Advanced Glycation End-product	75.7	60.2	0.045	0.393	-0.47
Arachidonoylcholine	Gr1	Fatty Acid Metabolism (Acyl Choline)	99.0	94.3	0.004	0.138	-0.47
cis-3,4-methyleneheptanoylglycine	Gr1	Fatty Acid Metabolism (Acyl Glycine)	94.7	93.2	0.001	0.068	-0.48
6-hydroxyindole sulfate	Gr2	Chemical	98.8	96.6	0.012	0.242	-0.48

Daidzein sulfate (2)	Gr2	Food Component/Plant	34.7	25.0	0.025	0.31	-0.49
Cysteine-glutathione disulfide	Gr3	Glutathione Metabolism	88.8	84.1	0.043	0.389	-0.50
Docosahexaenoylcholine	Gr1	Fatty Acid Metabolism (Acyl Choline)	84.3	68.2	0.012	0.242	-0.50
7-methylxanthine	Gr2	Xanthine Metabolism	88.0	86.4	0.023	0.298	-0.50
Androstenediol (3beta,17beta) monosulfate (1)	Gr1	Androgenic Steroids	90.6	87.5	0.039	0.371	-0.51
Dimethylguanidino valeric acid	Gr3	Urea cycle; Arginine and Proline Metabolism	79.4	69.3	0.050	0.408	-0.51
Palmitoleoyl-linoleoyl-glycerol (16:1/18:2) [1]	Gr1	Diacylglycerol	52.7	39.8	0.005	0.149	-0.52
Androstenediol (3beta,17beta) disulfate (1)	Gr1	Androgenic Steroids	99.6	100	0.044	0.39	-0.52
N-acetyl-3-methylhistidine	Gr3	Histidine Metabolism	73.9	67.0	0.009	0.232	-0.55
Metabolonic lactone sulfate	Gr8	Partially Characterized Molecules	54.7	53.4	0.031	0.352	-0.55
5alpha-androstan-3alpha,17beta-diol monosulfate (1)	Gr1	Androgenic Steroids	28.4	15.9	0.003	0.131	-0.56
N-acetylglucosamine conjugate of C24H40O4 bile acid	Gr8	Partially Characterized Molecules	65.9	48.9	0.019	0.285	-0.57
Vanillic acid glycine	Gr2	Food Component/Plant	75.7	53.4	0.014	0.253	-0.60
4-methylcatechol sulfate	Gr2	Benzoate Metabolism	98.8	98.9	0.040	0.379	-0.61
Isoursodeoxycholate sulfate (1)	Gr1	Secondary Bile Acid Metabolism	65.3	53.4	0.015	0.253	-0.62
1,5-anhydroglucitol	Gr7	Glycolysis, Gluconeogenesis, and Pyruvate Metabolism	99.8	100	0.002	0.107	-0.66
3-phenylpropionate (hydrocinnamate)	Gr2	Benzoate Metabolism	80.2	63.6	0.040	0.378	-0.68
Fibrinopeptide A, phosphono-ser(3)	Gr5	Fibrinogen Cleavage Peptide	87.1	80.7	<0.001	0.041	-0.68
Chenodeoxycholic acid sulfate (1)	Gr1	Primary Bile Acid Metabolism	69.0	51.1	0.002	0.107	-0.69
4-methylguaiacol sulfate	Gr2	Benzoate Metabolism	73.3	61.4	0.012	0.242	-0.70
4-acetylcatechol sulfate (1)	Gr2	Food Component/Plant	60.2	45.5	0.002	0.107	-0.73
5alpha-androstan-3beta,17beta-diol disulfate	Gr1	Androgenic Steroids	86.9	84.1	0.029	0.338	-0.75
Ursodeoxycholate	Gr1	Secondary Bile Acid Metabolism	96.1	93.2	0.025	0.31	-0.90
2-arachidonoylglycerol (20:4)	Gr1	Monoacylglycerol	61.2	38.6	<0.001	0.039	-1.03
Isoursodeoxycholate	Gr1	Secondary Bile Acid Metabolism	94.7	93.2	0.001	0.054	-1.05

Table S3. Changes in metabolites abundance in cases collected 12 (Cases-12M) or 6 (Cases-6M) months prior to HCC diagnosis compared to controls. Significance of metabolites abundance changes between controls and cases-12M or cases-6M was determined by linear mixed model. For these metabolites, significance between cases-24M and controls is also shown. Super-pathway groups are abbreviated as follows: Gr1: Lipids, Gr2: Xenobiotics, Gr3: Amino Acids: Gr5: Peptides, Gr6: Nucleotides, Gr7: Carbohydrates, Gr8: Partially Characterized Molecules. AFP: alpha-fetoprotein, C: group coefficient (log-transformed), *: not significant. The metabolites are separated into those with positive C and those with negative C.

			Cases-24M			Cases-12M			Cases-6M		
**Metabolite**	**Group**	**Pathway**	**p**	**q**	**C**	**p**	**q**	**C**	**p**	**q**	**C**
AFP			<0.001	<0.001	0.60	<0.001	<0.001	0.68	<0.001	<0.001	0.68
Asparagine	Gr3	Alanine and Aspartate Metabolism	0.001	0.074	0.16	0.004	0.213	0.15	0.014	0.381	0.14
Alanine	Gr3	Alanine and Aspartate Metabolism	0.002	0.082	0.15	0.003	0.180	0.16	0.010	0.337	0.14
testosterone sulfate	Gr1	Androgenic Steroids	0.003	0.095	0.76	0.003	0.180	0.81	0.001	0.090	0.95
propyl 4-hydroxybenzoate	Gr2	Benzoate Metabolism	*	*	*	0.039	0.437	0.42	0.035	0.541	0.49
deoxycarnitine	Gr1	Carnitine Metabolism	*	*	*	0.028	0.406	0.18	0.047	0.582	0.17
ceramide (d18:1/14:0, d16:1/16:0)	Gr1	Ceramides	*	*	*	0.024	0.377	0.24	0.024	0.503	0.26
Cortisone	Gr1	Corticosteroids	0.002	0.079	0.37	0.009	0.263	0.34	0.006	0.288	0.38
cortisol 21-sulfate	Gr1	Corticosteroids	0.008	0.165	0.20	0.015	0.321	0.20	0.003	0.229	0.25
14-HDoHE/17-HDoHE	Gr1	Docosanoid	0.030	0.322	0.50	0.020	0.351	0.58	0.013	0.359	0.68
salicyluric glucuronide	Gr2	Drug - Analgesics, Anesthetics	*	*	*	0.035	0.427	0.85	0.032	0.541	0.92
Lidocaine	Gr2	Drug - Analgesics, Anesthetics	<0.001	0.023	0.71	<0.001	0.054	0.74	<0.001	0.029	0.86
N-ethylglycinexylidide	Gr2	Drug - Analgesics, Anesthetics	<0.001	<0.001	0.59	<0.001	0.001	0.63	<0.001	<0.001	0.74
Ofloxacin	Gr2	Drug - Antibiotic	0.003	0.097	0.08	0.001	0.075	0.10	<0.001	0.075	0.12
Ondansetron	Gr2	Drug - Gastrointestinal	0.002	0.078	0.37	<0.001	0.054	0.47	<0.001	0.026	0.57
Famotidine	Gr2	Drug - Gastrointestinal	<0.001	0.022	0.12	<0.001	0.007	0.15	0.001	0.076	0.19
Atorvastatin	Gr2	Drug - Metabolic	0.035	0.346	0.36	0.016	0.331	0.45	0.025	0.503	0.44
p-hydroxyatorvastatin	Gr2	Drug - Metabolic	0.011	0.195	0.34	0.007	0.259	0.39	0.008	0.337	0.40
o-hydroxyatorvastatin	Gr2	Drug - Metabolic	0.023	0.276	0.30	0.008	0.260	0.38	0.011	0.337	0.39
ezetimibe glucuronide	Gr2	Drug - Metabolic	<0.001	0.001	0.06	<0.001	0.007	0.06	<0.001	<0.001	0.08
3-decenoylcarnitine	Gr1	Fatty Acid Metabolism (Acyl Carnitine, Monounsaturated)	0.004	0.100	0.53	0.001	0.092	0.64	0.002	0.130	0.64
Malonate	Gr1	Fatty Acid Synthesis	0.011	0.196	0.34	0.009	0.266	0.38	0.033	0.541	0.33
Pristanate	Gr1	Fatty Acid, Branched	0.002	0.076	0.80	0.014	0.321	0.68	0.012	0.356	0.74
(12 or 13)-methylmyristate (a15:0 or i15:0)	Gr1	Fatty Acid, Branched	0.014	0.208	0.48	0.008	0.263	0.56	0.005	0.288	0.63

(14 or 15)-methylpalmitate (a17:0 or i17:0)	Gr1	Fatty Acid, Branched	0.014	0.208	0.320	0.050	0.475	0.28	0.050	0.585	0.30
hexadecanedioate (C16-DC)	Gr1	Fatty Acid, Dicarboxylate	0.006	0.132	0.42	0.014	0.321	0.41	0.019	0.462	0.41
2-hydroxyheptanoate	Gr1	Fatty Acid, Monohydroxy	0.021	0.256	0.20	0.016	0.331	0.23	0.042	0.572	0.21
Phytanate	Gr2	Food Component/Plant	0.009	0.166	0.40	0.049	0.474	0.32	0.036	0.541	0.37
gamma-glutamyltyrosine	Gr5	Gamma-glutamyl Amino Acid	0.001	0.076	0.30	0.005	0.237	0.27	0.010	0.337	0.26
gamma-glutamylmethionine	Gr5	Gamma-glutamyl Amino Acid	0.004	0.097	0.29	0.015	0.321	0.26	0.010	0.337	0.28
gamma-glutamyltryptophan	Gr5	Gamma-glutamyl Amino Acid	0.002	0.082	0.27	0.006	0.256	0.26	0.007	0.332	0.27
gamma-glutamylserine	Gr5	Gamma-glutamyl Amino Acid	0.002	0.076	0.20	0.008	0.263	0.18	0.034	0.541	0.15
gamma-glutamylphenylalanine	Gr5	Gamma-glutamyl Amino Acid	0.021	0.256	0.18	0.041	0.437	0.17	0.043	0.572	0.18
Serine	Gr3	Glycine, Serine and Threonine Metabolism	0.001	0.076	0.16	0.008	0.263	0.14	0.033	0.541	0.12
Threonine	Gr3	Glycine, Serine and Threonine Metabolism	0.005	0.121	0.15	0.013	0.321	0.15	0.025	0.504	0.134
Pyruvate	Gr7	Glycolysis, Gluconeogenesis, and Pyruvate Metabolism	0.001	0.076	0.34	0.003	0.180	0.34	0.012	0.358	0.30
imidazole lactate	Gr3	Histidine Metabolism	*	*	*	0.029	0.417	0.24	0.034	0.541	0.24
Formiminoglutamate	Gr3	Histidine Metabolism	0.014	0.208	0.28	0.029	0.417	0.27	0.007	0.330	0.36
alpha-hydroxyisocaproate	Gr3	Leucine, Isoleucine and Valine Metabolism	0.010	0.181	0.32	0.019	0.342	0.31	0.023	0.502	0.32
caprate (10:0)	Gr1	Medium Chain Fatty Acid	0.014	0.209	0.39	0.016	0.331	0.42	0.015	0.398	0.45
10-undecenoate (11:1n1)	Gr1	Medium Chain Fatty Acid	0.009	0.166	0.30	0.047	0.470	0.25	0.018	0.441	0.31
Cystathionine	Gr3	Methionine, Cysteine, SAM and Taurine Metabolism	0.016	0.225	0.42	0.037	0.430	0.39	0.043	0.572	0.40
Methionine	Gr3	Methionine, Cysteine, SAM and Taurine Metabolism	0.001	0.074	0.25	0.006	0.258	0.21	0.011	0.337	0.21
methionine sulfoxide	Gr3	Methionine, Cysteine, SAM and Taurine Metabolism	0.013	0.208	0.23	0.033	0.424	0.21	0.046	0.582	0.21
branched-chain, straight-chain, or cyclopropyl 10:1 fatty acid (1)	Gr8	Partially Characterized Molecules	0.049	0.399	0.37	0.033	0.424	0.43	0.026	0.505	0.47
Xylose	Gr7	Pentose Metabolism	*	*	*	0.034	0.427	0.45	0.017	0.429	0.55
Phenylalanine	Gr3	Phenylalanine Metabolism	0.002	0.076	0.14	0.007	0.258	0.12	0.015	0.398	0.12
1-(1-enyl-stearoyl)-2-oleoyl-GPE (P-18:0/18:1)	Gr1	Plasmalogen	0.033	0.340	0.19	0.033	0.424	0.20	0.050	0.585	0.19
1-(1-enyl-palmitoyl)-2-oleoyl-GPE (P-16:0/18:1)	Gr1	Plasmalogen	0.027	0.300	0.18	0.021	0.359	0.20	0.010	0.337	0.23
21-hydroxypregnenolone monosulfate (2)	Gr1	Pregnenolone Steroids	*	*	*	0.041	0.437	0.36	0.026	0.504	0.41
taurochenodeoxycholate	Gr1	Primary Bile Acid Metabolism	0.007	0.153	1.02	0.012	0.304	1.01	0.026	0.505	0.93
methylphosphate	Gr6	Purine and Pyrimidine Metabolism	0.008	0.166	0.23	0.018	0.342	0.22	0.048	0.585	0.19
Xanthosine	Gr6	Purine Metabolism, (Hypo)Xanthine/Inosine containing	*	*	*	0.006	0.243	0.54	0.010	0.337	0.52
2'-O-methylcytidine	Gr6	Pyrimidine Metabolism, Cytidine containing	0.017	0.232	0.22	0.023	0.375	0.22	0.050	0.585	0.20
Taurohyocholate	Gr1	Secondary Bile Acid Metabolism	<0.001	0.022	1.86	<0.001	0.054	1.87	0.001	0.084	1.81
taurochenodeoxycholic acid 3-sulfate	Gr1	Secondary Bile Acid Metabolism	0.006	0.145	0.99	0.019	0.342	0.90	0.034	0.541	0.85
Glycohyocholate	Gr1	Secondary Bile Acid Metabolism	0.011	0.196	0.84	0.015	0.321	0.86	0.043	0.572	0.74

taurocholenate sulfate	Gr1	Secondary Bile Acid Metabolism	0.001	0.076	0.61	0.001	0.115	0.65	0.001	0.110	0.69
beta-sitosterol	Gr1	Sterol	0.001	0.076	0.71	0.003	0.196	0.68	0.002	0.157	0.74
Campesterol	Gr1	Sterol	0.022	0.265	0.57	0.042	0.442	0.53	0.035	0.541	0.58
N-acetyltryptophan	Gr3	Tryptophan Metabolism	*	*	*	0.040	0.437	0.22	0.044	0.572	0.24
Picolinate	Gr3	Tryptophan Metabolism	<0.001	0.022	0.44	0.001	0.075	0.42	0.001	0.076	0.45
Tyrosine	Gr3	Tyrosine Metabolism	0.005	0.115	0.21	0.015	0.321	0.20	0.041	0.572	0.17
N,N-dimethylalanine	Gr3	Alanine and Aspartate Metabolism	0.045	0.386	-0.41	0.019	0.342	-0.51	0.034	0.541	-0.48
5alpha-androstan-3alpha,17beta-diol monosulfate (1)	Gr1	Androgenic Steroids	0.001	0.076	-0.71	0.003	0.195	-0.70	0.005	0.288	-0.70
androstenediol (3alpha, 17alpha) monosulfate (3)	Gr1	Androgenic Steroids	0.014	0.208	-0.80	0.019	0.342	-0.81	0.036	0.541	-0.76
4-methylguaiacol sulfate	Gr2	Benzoate Metabolism	0.021	0.256	-0.76	0.014	0.321	-0.88	0.023	0.501	-0.88
3-phenylpropionate (hydrocinnamate)	Gr2	Benzoate Metabolism	0.004	0.107	-1.10	0.001	0.092	-1.36	<0.001	0.037	-1.61
myristoyl-linoleoyl-glycerol (14:0/18:2) [1]	Gr1	Diacylglycerol	0.013	0.208	-0.38	0.009	0.266	-0.43	0.007	0.330	-0.47
linoleoyl-linoleoyl-glycerol (18:2/18:2) [1]	Gr1	Diacylglycerol	0.020	0.253	-0.46	0.028	0.410	-0.46	0.043	0.572	-0.46
palmitoleoyl-linoleoyl-glycerol (16:1/18:2) [1]	Gr1	Diacylglycerol	0.003	0.087	-0.65	0.002	0.126	-0.73	0.002	0.130	-0.76
palmitoloelycholine	Gr1	Fatty Acid Metabolism (Acyl Choline)	0.003	0.087	-0.53	0.006	0.243	-0.52	0.028	0.521	-0.44
Linoleoylcholine	Gr1	Fatty Acid Metabolism (Acyl Choline)	0.001	0.074	-0.55	0.040	0.437	-0.36	0.048	0.585	-0.36
docosahexaenoylcholine	Gr1	Fatty Acid Metabolism (Acyl Choline)	0.001	0.076	-0.75	0.003	0.180	-0.74	0.002	0.168	-0.79
cis-3,4-methyleneheptanoylglycine	Gr1	Fatty Acid Metabolism (Acyl Glycine)	0.003	0.097	-0.49	0.011	0.287	-0.46	0.018	0.448	-0.45
Butyrylglycine	Gr1	Fatty Acid Metabolism (also BCAA Metabolism)	0.013	0.208	-0.40	0.036	0.430	-0.36	0.028	0.523	-0.40
Malonylcarnitine	Gr1	Fatty Acid Synthesis	0.012	0.205	-0.34	0.026	0.397	-0.33	0.009	0.337	-0.41
fibrinopeptide A, phosphono-ser(3)	Gr5	Fibrinogen Cleavage Peptide	0.001	0.074	-0.75	0.001	0.092	-0.77	0.002	0.157	-0.76
ferulylglycine (2)	Gr2	Food Component/Plant	0.010	0.189	-0.51	0.025	0.377	-0.48	0.036	0.541	-0.48
4-acetylcatechol sulfate (1)	Gr2	Food Component/Plant	0.020	0.256	-0.65	0.035	0.428	-0.65	0.031	0.541	-0.72
Cinnamoylglycine	Gr2	Food Component/Plant	*	*	*	0.049	0.474	-0.77	0.043	0.572	-0.84
N-acetylglycine	Gr3	Glycine, Serine and Threonine Metabolism	<0.001	0.023	-0.32	<0.001	0.054	-0.33	0.001	0.095	-0.32
1,5-anhydroglucitol (1,5-AG)	Gr7	Glycolysis, Gluconeogenesis, and Pyruvate Metabolism	<0.001	0.042	-0.89	0.010	0.266	-0.67	0.010	0.337	-0.70
1-palmitoyl-GPC (16:0)	Gr1	Lysophospholipid	0.007	0.153	-0.13	0.017	0.342	-0.12	0.040	0.572	-0.11
2-palmitoyl-GPC (16:0)	Gr1	Lysophospholipid	0.009	0.166	-0.22	0.010	0.266	-0.23	0.023	0.503	-0.21
1-palmitoleoyl-GPC (16:1)	Gr1	Lysophospholipid	0.008	0.162	-0.22	0.007	0.259	-0.24	0.024	0.503	-0.22
2-arachidonoylglycerol (20:4)	Gr1	Monoacylglycerol	0.001	0.074	-1.09	0.001	0.110	-1.12	0.005	0.281	-1.04
metabolonic lactone sulfate	Gr8	Partially Characterized Molecules	0.002	0.078	-0.91	0.004	0.198	-0.90	0.010	0.337	-0.84
glycerophosphorylcholine (GPC)	Gr1	Phospholipid Metabolism	0.002	0.076	-0.20	0.004	0.213	-0.19	0.021	0.483	-0.16
chenodeoxycholic acid sulfate (1)	Gr1	Primary Bile Acid Metabolism	0.002	0.079	-0.81	0.015	0.325	-0.69	0.037	0.541	-0.63
Isoursodeoxycholate	Gr1	Secondary Bile Acid Metabolism	0.003	0.086	-1.08	0.009	0.263	-1.02	0.014	0.381	-1.00
6-bromotryptophan	Gr3	Tryptophan Metabolism	0.006	0.135	-0.25	0.026	0.388	-0.22	0.022	0.496	-0.23
phenol sulfate	Gr3	Tyrosine Metabolism	0.019	0.253	-0.55	0.027	0.404	-0.55	0.007	0.332	-0.71

Table S4. Metabolites significantly affected by PNPLA3 SNP, TM6SF2 SNP or Gender. Metabolite significance was determined with a linear-mixed model. Super-pathway groups are abbreviated as follows: Gr1: Lipids, Gr2: Xenobiotics, Gr3: Amino Acids: Gr5: Peptides, Gr6: Nucleotides, Gr7: Carbohydrates, Gr8: Partially Characterized Molecules. C: coefficient for the clinical variable (log-transformed). The metabolites are sectioned into being significantly affected by PNPLA3, TM6SF2, or Gender. The metabolites are further separated into those with positive C and those with negative C.

Metabolite Name	Group	Pathway	p	Q	C
**PNPLA3**					
taurohyocholate	Gr1	Secondary Bile Acid Metabolism	0.009	0.117	1.14
taurochenodeoxycholic acid 3-sulfate	Gr1	Secondary Bile Acid Metabolism	0.007	0.117	0.89
glycohyocholate	Gr1	Secondary Bile Acid Metabolism	0.005	0.117	0.83
taurochenodeoxycholate	Gr1	Primary Bile Acid Metabolism	0.020	0.154	0.82
taurocholate	Gr1	Primary Bile Acid Metabolism	0.034	0.191	0.76
alpha-hydroxyisocaproate	Gr3	Leucine, Isoleucine and Valine Metabolism	0.019	0.154	0.25
gamma-glutamylmethionine	Gr5	Gamma-glutamyl Amino Acid	0.010	0.117	0.23
2'-O-methylcytidine	Gr6	Pyrimidine Metabolism, Cytidine containing	0.021	0.154	0.23
ondansetron	Gr2	Drug - Gastrointestinal	0.014	0.151	0.22
methylphosphate	Gr6	Purine and Pyrimidine Metabolism	0.010	0.117	0.21
1-linoleoyl-GPA (18:2)*	Gr1	Lysophospholipid	0.039	0.200	0.19
methionine	Gr3	Methionine, Cysteine, SAM and Taurine Metabolism	0.007	0.117	0.18
Tyrosine	Gr3	Tyrosine Metabolism	0.030	0.188	0.15
gamma-glutamylserine	Gr5	Gamma-glutamyl Amino Acid	0.006	0.117	0.13
asparagine	Gr3	Alanine and Aspartate Metabolism	0.005	0.117	0.12
Serine	Gr3	Glycine, Serine and Threonine Metabolism	0.022	0.156	0.10
phenylalanine	Gr3	Phenylalanine Metabolism	0.023	0.156	0.09
1-arachidonoyl-GPC (20:4n6)	Gr1	Lysophospholipid	0.006	0.117	-0.24
1-arachidonylglycerol (20:4)	Gr1	Monoacylglycerol	0.028	0.180	-0.27
dihomo-linolenoyl-choline	Gr1	Fatty Acid Metabolism (Acyl Choline)	0.034	0.191	-0.38
arachidonoylcholine	Gr1	Fatty Acid Metabolism (Acyl Choline)	0.015	0.151	-0.42
fibrinopeptide A, phosphono-ser(3)	Gr5	Fibrinogen Cleavage Peptide	0.017	0.154	-0.48
1-docosahexaenoylglycerol (22:6)	Gr1	Monoacylglycerol	0.007	0.117	-0.51
1-methylurate	Gr2	Xanthine Metabolism	0.004	0.117	-0.52
metabolonic lactone sulfate	Gr8	Partially Characterized Molecules	0.002	0.117	-0.87
**TM6SF2**					
5,6-dihydrouracil	Gr6	Pyrimidine Metabolism, Uracil containing	0.043	0.421	0.29
alpha-hydroxyisocaproate	Gr3	Leucine, Isoleucine and Valine Metabolism	0.018	0.421	0.21
gamma-glutamylmethionine	Gr5	Gamma-glutamyl Amino Acid	0.041	0.421	0.16
methionine	Gr3	Methionine, Cysteine, SAM and Taurine Metabolism	0.023	0.421	0.13
1-(1-enyl-palmitoyl)-2-oleoyl-GPE (P-16:0/18:1)	Gr1	Plasmalogen	0.034	0.421	0.12
asparagine	Gr3	Alanine and Aspartate Metabolism	0.042	0.421	0.08
nateglinide	Gr2	Drug - Metabolic	0.044	0.421	0.01
N-acetyltaurine	Gr3	Methionine, Cysteine, SAM and Taurine Metabolism	0.023	0.421	-0.21
gamma-glutamylglutamate	Gr5	Gamma-glutamyl Amino Acid	0.004	0.303	-0.29

Acisoga	Gr3	Polyamine Metabolism	0.016	0.421	-0.29
5alpha-androstan-3alpha,17beta-diol monosulfate (1)	Gr1	Androgenic Steroids	0.033	0.421	-0.38
tetrahydrocortisol sulfate (1)	Gr1	Corticosteroids	0.01	0.421	-0.42
androstenediol (3beta,17beta) disulfate (1)	Gr1	Androgenic Steroids	0.003	0.303	-0.70
5alpha-androstan-3beta,17beta-diol disulfate	Gr1	Androgenic Steroids	0.024	0.421	-0.71
**Gender**					
5alpha-androstan-3beta,17beta-diol disulfate	Gr1	Androgenic Steroids	<0.001	<0.001	2.48
testosterone sulfate	Gr1	Androgenic Steroids	<0.001	<0.001	1.38
5alpha-androstan-3alpha,17beta-diol 17-glucuronide	Gr1	Androgenic Steroids	<0.001	<0.001	1.01
epiandrosterone sulfate	Gr1	Androgenic Steroids	<0.001	<0.001	1.00
androstenediol (3beta,17beta) disulfate (1)	Gr1	Androgenic Steroids	<0.001	<0.001	0.96
androstenediol (3beta,17beta) monosulfate (1)	Gr1	Androgenic Steroids	<0.001	<0.001	0.91
5alpha-androstan-3alpha,17beta-diol monosulfate (1)	Gr1	Androgenic Steroids	<0.001	<0.001	0.60
metabolonic lactone sulfate	Gr8	Partially Characterized Molecules	0.027	0.137	0.43
5alpha-pregnan-3beta,20alpha-diol monosulfate (2)	Gr1	Progestin Steroids	0.005	0.046	0.39
3-decenoylcarnitine	Gr1	Fatty Acid Metabolism (Acyl Carnitine, Monounsaturated)	0.002	0.024	0.36
7-methylxanthine	Gr2	Xanthine Metabolism	0.023	0.123	0.36
tetrahydrocortisol sulfate (1)	Gr1	Corticosteroids	0.008	0.058	0.36
3,7-dimethylurate	Gr2	Xanthine Metabolism	0.035	0.162	0.30
phytanate	Gr2	Food Component/Plant	0.007	0.057	0.26
cortisone	Gr1	Corticosteroids	0.003	0.036	0.23
alpha-hydroxyisocaproate	Gr3	Leucine, Isoleucine and Valine Metabolism	0.003	0.036	0.22
N-acetyl-aspartyl-glutamate (NAAG)	Gr3	Glutamate Metabolism	<0.001	0.005	0.22
picolinate	Gr3	Tryptophan Metabolism	0.010	0.065	0.19
homoarginine	Gr3	Urea cycle; Arginine and Proline Metabolism	0.010	0.065	0.17
2-aminobutyrate	Gr3	Glutathione Metabolism	0.019	0.107	0.12
beta-alanine	Gr6	Pyrimidine Metabolism, Uracil containing	0.017	0.099	0.10
Alanine	Gr3	Alanine and Aspartate Metabolism	0.035	0.162	0.06
1-palmitoleoyl-GPC (16:1)	Gr1	Lysophospholipid	0.014	0.085	-0.13
N-acetylglycine	Gr3	Glycine, Serine and Threonine Metabolism	0.005	0.046	-0.15
1-linoleoyl-GPA (18:2)	Gr1	Lysophospholipid	0.008	0.058	-0.17
vanillactate	Gr3	Tyrosine Metabolism	0.017	0.099	-0.18
2-hydroxysebacate	Gr1	Fatty Acid, Dicarboxylate	0.027	0.137	-0.19
cis-3,4-methyleneheptanoylglycine	Gr1	Fatty Acid Metabolism (Acyl Glycine)	0.064	0.242	-0.20
propionylglycine	Gr1	Fatty Acid Metabolism (also BCAA Metabolism)	0.049	0.209	-0.21
2-palmitoleoyl-GPC (16:1)	Gr1	Lysophospholipid	0.002	0.024	-0.22
1-arachidonylglycerol (20:4)	Gr1	Monoacylglycerol	<0.001	0.005	-0.32
isoursodeoxycholate	Gr1	Secondary Bile Acid Metabolism	0.045	0.198	-0.45
1-docosahexaenoylglycerol (22:6)	Gr1	Monoacylglycerol	<0.001	0.005	-0.50
N-acetylglucosamine conjugate of C24H40O4 bile acid	Gr8	Partially Characterized Molecules	0.005	0.046	-0.50
isoursodeoxycholate sulfate (1)	Gr1	Secondary Bile Acid Metabolism	0.005	0.046	-0.52
ursodeoxycholate	Gr1	Secondary Bile Acid Metabolism	0.034	0.162	-0.63

Table S5. Abundance changes in metabolites between Controls with LIRAD-3 lesions (Controls-LR3) and Cases with LIRAD-3 lesions (Cases-LR3). Metabolites among the 150 HCC-associated

metabolites identified in **Table S2**, which had significant differential abundance between Cases-LR3 and Controls-LR3, as determined by linear mixed-effects model analysis, are listed. Super-pathway groups for metabolites are abbreviated as follows: Gr1: Lipids, Gr2: Xenobiotics, Gr3: Amino Acids, Gr5: Peptides, Gr6: Nucleotides, Gr7: Carbohydrates. AFP: Alpha-fetoprotein, GPE: Glycerophosphorylethanolamine, GPC: Glycerophosphorylcholine, C: group coefficient (log-transformed). Metabolites are ordered from highest to lowest C.

Metabolite	Group	Pathway	p	q	C
1,2-dilinoleoyl-GPE (18:2/18:2)	Gr1	Phosphatidylethanolamine (PE)	0.020	0.231	0.71
Malonate	Gr1	Fatty Acid Synthesis	0.007	0.186	0.71
AFP			0.043	0.268	0.51
Pipecolate	Gr3	Lysine Metabolism	0.017	0.212	0.48
Picolinate	Gr3	Tryptophan Metabolism	0.013	0.204	0.43
Pyruvate	Gr7	Glycolysis, Gluconeogenesis, and Pyruvate Metabolism	0.011	0.188	0.42
gamma-glutamyltyrosine	Gr5	Gamma-glutamyl Amino Acid	0.033	0.239	0.30
beta-alanine	Gr6	Pyrimidine Metabolism, Uracil containing	0.011	0.188	0.26
methionine sulfoxide	Gr3	Methionine, Cysteine, SAM and Taurine Metabolism	0.015	0.204	0.24
gamma-glutamyltryptophan	Gr5	Gamma-glutamyl Amino Acid	0.029	0.237	0.23
1-palmitoyl-GPC (16:0)	Gr1	Lysophospholipid	0.006	0.186	-0.20
N-acetylglycine	Gr3	Glycine, Serine and Threonine Metabolism	0.027	0.237	-0.24
GPC	Gr1	Phospholipid Metabolism	0.011	0.188	-0.28
phenylalanylglycine	Gr5	Dipeptide	0.044	0.268	-0.30
arachidonoyl ethanolamide	Gr1	Endocannabinoid	0.028	0.238	-0.34
1-arachidonoyl-GPC (20:4n6)	Gr1	Lysophospholipid	0.005	0.186	-0.41
N-octanoylglycine	Gr1	Fatty Acid Metabolism (Acyl Glycine)	0.028	0.238	-0.50
1-(1-enyl-palmitoyl)-GPC (P-16:0)	Gr1	Lysoplasmalogen	<0.001	0.024	-0.50
palmitoylcholine	Gr1	Fatty Acid Metabolism (Acyl Choline)	0.031	0.237	-0.54
docosahexaenoylcholine	Gr1	Fatty Acid Metabolism (Acyl Choline)	0.037	0.255	-0.80
arachidonoylcholine	Gr1	Fatty Acid Metabolism (Acyl Choline)	0.006	0.186	-0.86
dihomo-linolenoyl-choline	Gr1	Fatty Acid Metabolism (Acyl Choline)	0.001	0.078	-0.86
phenol sulfate	Gr3	Tyrosine Metabolism	0.021	0.231	-0.98

Supplementary results

Effect of treatment on identified metabolites changes

The inclusion in this study of samples collected after HCC diagnosis and treatment for some of the patients who developed HCC during surveillance (Cases-T), allowed us to determine for which of the metabolites identified as significantly increased or decreased in Cases vs Controls, a reverse phenotype was observed after treatment. AFP was not significantly decreased in Cases-T compared to Cases. Among the 64 metabolites significantly increased in Cases vs Controls, 33 showed significant abundance decrease after treatment in Cases-T vs Cases (**Supplementary Results Table; Supplementary Results Figure panel A**). Significance remained after adjusting for FDR for 32 of these 33 metabolites (**Supplementary Results Figure panel A**). These were largely metabolites associated with Amino Acids metabolism, Bile Acid metabolism and Purine & Pyrimidine metabolism, supporting a major role of these pathways in liver progression from cirrhosis to HCC. The largest decreases were observed for tauro-conjugated bile acids: taurohyocholate ($C=-2.80$, $p<0.001$, $q<0.001$), taurocholate ($C=-2.77$, $p<0.001$, $q<0.001$), taurochenodeoxycholate ($C=-2.22$, $p<0.001$, $q<0.001$), taurochenodeoxycholic acid 3-sulfate ($C=-2.06$, $p<0.001$, $q<0.001$) and taurocholenate sulfate ($C=-1.15$, $p<0.001$, $q<0.001$). Among the 86 metabolites significantly decreased in Cases vs Controls, 44 showed significant abundance increase after treatment in Cases-T vs Cases, with 36 of them remaining significant after adjusting for FDR (**Supplementary Results Table; Supplementary Results Figure panel A**). The largest increase was observed for isoursodeoxycholate ($C=2.09$, $p<0.001$, $q<0.001$). Interestingly, positive interactions with time to last visit in Cases and inverse negative interactions with time in Cases-T, were observed in some of the metabolites from **Supplementary Results Table**. These included homoarginine and the tauro-conjugated bile acids taurocholate, taurochenodeoxycholate and taurohyocholate (**Supplementary Results Figure panel B**).

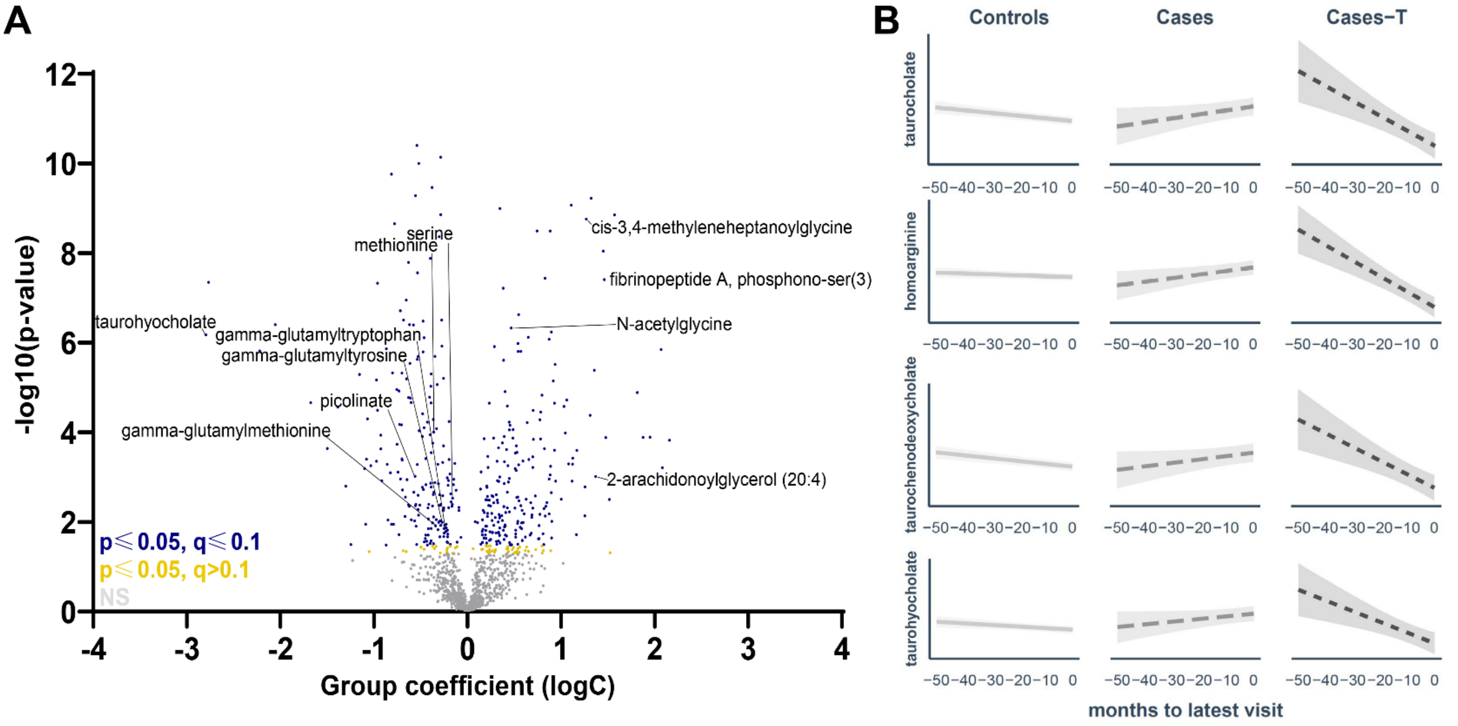

Supplementary Results Figure. Metabolite abundance changes in Cases following HCC treatment. (A) Volcano plot for differential metabolites, between Cases (samples collected pre-HCC) and Cases-T (samples collected after HCC treatment). The group coefficient (Cases-T vs Cases) for log-transformed metabolite levels (x-axis) and minus log10 p-values (y-axis) are shown. Metabolites that remained significant (p≤0.05, q≤0.1) after adjustment with the Benjamini–Hochberg method are shown in blue. Metabolites that were not significant (p≤0.05, q>0.1) after FDR adjustment are shown in yellow. **(B)** Interaction plots showing metabolite levels over time by group, with regression line and 95% confidence intervals. Metabolite level were plotted against months to latest visit for selected metabolites; taurocholate, within Controls, Cases and Cases-T.

Supplementary Results Table. Abundance changes in metabolites from Table S2 after HCC treatment. Significance of metabolites abundance changes between cases (samples collected prior to HCC diagnosis) and cases-T (samples collected after HCC treatment) was determined by linear mixed model. Super-pathway groups for metabolites are abbreviated as follows: Gr1: Lipids, Gr2: Xenobiotics, Gr3: Amino Acids: Gr4: Cofactors and Vitamins, Gr5: Peptides, Gr6: Nucleotides, Gr7: Carbohydrates,

Gr8: Partially Characterized Molecules. Cases (%) and Cases-T (%) reflect the percentage of detection of each metabolite in cases and cases-T samples, respectively. GPA: Glycerophosphatidic acid, CEHC: carboxyethyl-hydroxychroman, logC: group coefficient (log values). The metabolites are ordered from highest to lowest logC.

Metabolite	Group	Pathway	Cases (%)	Cases-T (%)	p	q	logC
isoursodeoxycholate	Gr1	Secondary Bile Acid Metabolism	93.2	96.8	<0.001	<0.001	2.068
daidzein sulfate (2)	Gr2	Food Component/Plant	25.0	45.2	<0.001	<0.001	1.814
fibrinopeptide A, phosphono-ser(3)	Gr5	Fibrinogen Cleavage Peptide	80.7	90.3	<0.001	<0.001	1.461
2-arachidonoylglycerol (20:4)	Gr1	Monoacylglycerol	38.6	83.9	0.001	0.007	1.370
glycine conjugate of C10H12O2	Gr8	Partially Characterized Molecules	70.5	93.5	<0.001	<0.001	1.322
cysteine-glutathione disulfide	Gr3	Glutathione Metabolism	84.1	100	<0.001	0.001	1.310
cis-3,4-methyleneheptanoylglycine	Gr1	Fatty Acid Metabolism (Acyl Glycine)	93.2	100	<0.001	<0.001	1.268
N6-carboxymethyllysine	Gr7	Advanced Glycation End-product	60.2	90.3	0.002	0.010	1.256
ursodeoxycholate	Gr1	Secondary Bile Acid Metabolism	93.2	90.3	0.007	0.032	1.254
trimethylamine N-oxide	Gr1	Phospholipid Metabolism	100	100	<0.001	0.003	1.125
6-hydroxyindole sulfate	Gr2	Chemical	96.6	100	0.001	0.004	1.121
isoursodeoxycholate sulfate (1)	Gr1	Secondary Bile Acid Metabolism	53.4	77.4	0.001	0.008	1.117
metabolonic lactone sulfate	Gr8	Partially Characterized Molecules	53.4	58.1	0.001	0.004	1.080
dimethylguanidino valeric acid	Gr3	Urea cycle; Arginine and Proline Metabolism	69.3	77.4	0.001	0.008	0.942
androstenediol (3beta,17beta) disulfate (1)	Gr1	Androgenic Steroids	100	100	<0.001	<0.001	0.917
7-methylxanthine	Gr2	Xanthine Metabolism	86.4	83.9	0.002	0.013	0.897
3-indoxyl sulfate	Gr3	Tryptophan Metabolism	100.0	100	0.001	0.006	0.883
4-acetylphenol sulfate	Gr2	Benzoate Metabolism	85.2	93.5	0.007	0.030	0.871
hexanoylglycine	Gr1	Fatty Acid Metabolism (Acyl Glycine)	21.6	64.5	<0.001	<0.001	0.871
androstenediol (3beta,17beta) monosulfate (1)	Gr1	Androgenic Steroids	87.5	80.6	0.004	0.022	0.836
4-methylcatechol sulfate	Gr2	Benzoate Metabolism	98.9	100	0.050	0.133	0.798
retinal	Gr4	Vitamin A Metabolism	90.9	100	<0.001	<0.001	0.796
isobutyrylglycine	Gr3	Leucine, Isoleucine and Valine Metabolism	48.9	64.5	<0.001	<0.001	0.783
acisoga	Gr3	Polyamine Metabolism	88.6	100	<0.001	0.001	0.781
1,5-anhydroglucitol (1,5-AG)	Gr7	Glycolysis, Gluconeogenesis, and Pyruvate Metabolism	100	100	<0.001	0.001	0.703
pimeloylcarnitine/3-methyladipoylcarnitine (C7-DC)	Gr1	Fatty Acid Metabolism (Acyl Carnitine, Dicarboxylate)	88.6	100	0.001	0.005	0.696
glycine conjugate of C10H14O2 (1)	Gr8	Partially Characterized Molecules	100	100	<0.001	0.000	0.649

chenodeoxycholic acid sulfate (1)	Gr1	Primary Bile Acid Metabolism	51.1	77.4	0.036	0.107	0.632
epiandrosterone sulfate	Gr1	Androgenic Steroids	100	100	0.007	0.030	0.605
docosahexaenoylcholine	Gr1	Fatty Acid Metabolism (Acyl Choline)	68.2	90.3	0.026	0.084	0.585
N-acetylglucosamine conjugate of C24H40O4 bile acid	Gr8	Partially Characterized Molecules	48.9	83.9	0.036	0.107	0.556
arachidonoylcholine	Gr1	Fatty Acid Metabolism (Acyl Choline)	94.3	100	0.047	0.129	0.553
2-hydroxyphenylacetate	Gr3	Phenylalanine Metabolism	83.0	100	0.003	0.018	0.550
gulonate	Gr4	Ascorbate and Aldarate Metabolism	94.3	100	<0.001	<0.001	0.546
4-hydroxyhippurate	Gr2	Benzoate Metabolism	100	100	0.010	0.040	0.536
delta-CEHC	Gr4	Tocopherol Metabolism	84.1	90.3	0.003	0.016	0.536
5alpha-androstan-3beta,17beta-diol disulfate	Gr1	Androgenic Steroids	84.1	80.6	0.050	0.134	0.528
3,7-dimethylurate	Gr2	Xanthine Metabolism	54.5	71.0	0.046	0.126	0.526
N-acetyl-aspartyl-glutamate	Gr3	Glutamate Metabolism	89.8	93.5	<0.001	0.001	0.486
N-acetylglycine	Gr3	Glycine, Serine and Threonine Metabolism	100	100	<0.001	<0.001	0.467
1-arachidonoyl-GPC (20:4n6)	Gr1	Lysophospholipid	100	100	0.001	0.007	0.447
arachidonoyl ethanolamide	Gr1	Endocannabinoid	30.7	87.1	<0.001	0.001	0.447
N-acetyltaurine	Gr3	Methionine, Cysteine, SAM and Taurine Metabolism	98.9	100	0.034	0.104	0.243
vanillactate	Gr3	Tyrosine Metabolism	96.6	93.5	0.047	0.128	0.214
lysine	Gr3	Lysine Metabolism	100	100	<0.001	0.004	-0.136
serine	Gr3	Glycine, Serine and Threonine Metabolism	100	100	0.003	0.016	-0.160
phenylalanine	Gr3	Phenylalanine Metabolism	100	100	0.002	0.014	-0.179
asparagine	Gr3	Alanine and Aspartate Metabolism	100	100	0.002	0.010	-0.208
threonine	Gr3	Glycine, Serine and Threonine Metabolism	100	100	0.001	0.007	-0.217
methylphosphate	Gr6	Purine and Pyrimidine Metabolism	100	100	0.011	0.044	-0.227
gamma-glutamyltryptophan	Gr5	Gamma-glutamyl Amino Acid	100	100	0.016	0.059	-0.233
1-(1-enyl-palmitoyl)-2-oleoyl-GPE (P-16:0/18:1)	Gr1	Plasmalogen	98.9	100	0.012	0.046	-0.246
adenine	Gr6	Purine Metabolism, Adenine containing	100	100	<0.001	0.002	-0.257
gamma-glutamyltyrosine	Gr5	Gamma-glutamyl Amino Acid	100	100	0.010	0.041	-0.272
methionine sulfoxide	Gr3	Methionine, Cysteine, SAM and Taurine Metabolism	100	100	0.010	0.039	-0.294
4-hydroxyphenylpyruvate	Gr3	Tyrosine Metabolism	100	100	0.021	0.073	-0.296
gamma-glutamylmethionine	Gr5	Gamma-glutamyl Amino Acid	100	100	0.012	0.047	-0.341
methionine	Gr3	Methionine, Cysteine, SAM and Taurine Metabolism	100	100	<0.001	0.001	-0.361
phytanate	Gr2	Food Component/Plant	100	100	0.035	0.105	-0.375
testosterone sulfate	Gr1	Androgenic Steroids	53.4	29.0	0.032	0.099	-0.430
sphingosine	Gr1	Sphingosines	98.9	96.8	<0.001	0.001	-0.430
alpha-hydroxyisocaproate	Gr3	Leucine, Isoleucine and Valine Metabolism	100	100	<0.001	0.003	-0.446

1-linoleoyl-GPA (18:2)	Gr1	Lysophospholipid	100	100	0.002	0.011	-0.471
tyrosine	Gr3	Tyrosine Metabolism	100	100	<0.001	<0.001	-0.475
picolinate	Gr3	Tryptophan Metabolism	100	100	0.001	0.007	-0.562
2'-O-methylcytidine	Gr6	Pyrimidine Metabolism, Cytidine containing	100	90.3	<0.001	<0.001	-0.656
5,6-dihydrouracil	Gr6	Pyrimidine Metabolism, Uracil containing	96.6	83.9	0.008	0.033	-0.668
hexadecanedioate (C16-DC)	Gr1	Fatty Acid, Dicarboxylate	98.9	100	<0.001	0.003	-0.702
pristanate	Gr1	Fatty Acid, Branched	68.2	77.4	0.019	0.067	-0.732
homoarginine	Gr3	Urea cycle; Arginine and Proline Metabolism	100	100	<0.001	<0.001	-0.814
beta-sitosterol	Gr1	Sterol	71.6	83.9	<0.001	<0.001	-0.868
taurocholenate sulfate	Gr1	Secondary Bile Acid Metabolism	100	100	<0.001	<0.001	-1.154
glycohyocholate	Gr1	Secondary Bile Acid Metabolism	98.9	93.5	<0.001	<0.001	-1.676
taurochenodeoxycholic acid 3-sulfate	Gr1	Secondary Bile Acid Metabolism	95.5	96.8	<0.001	<0.001	-2.056
taurochenodeoxycholate	Gr1	Primary Bile Acid Metabolism	100	100	<0.001	<0.001	-2.222
taurocholate	Gr1	Primary Bile Acid Metabolism	100	96.8	<0.001	<0.001	-2.767
taurohyocholate	Gr1	Secondary Bile Acid Metabolism	87.5	61.3	<0.001	<0.001	-2.796
