# Supplementary material for: Metabolomics biomarkers of hepatocellular carcinoma in a prospective cohort of patients with cirrhosis
Source: JHEP Rep. 2024 May 15;6(8):101119. doi: 10.1016/j.jhepr.2024.101119 (PMC11321296; doi:10.1016/j.jhepr.2024.101119)
Supplement: Multimedia component 4 [file mmc4.pdf]

# Metabolomics biomarkers of hepatocellular carcinoma in a prospective cohort of patients with cirrhosis

Jessica I. Sanchez[1], Antoine C. Fontillas[1], Suet-Ying Kwan[1], Caren I. Sanchez[1], Tiffany L. Calderone[1], Jana L. Lee[2], Ahmed Elsaiey[3], Darrel W. Cleere[3], Peng Wei[4], John M. Vierling[2], David W. Victor[3], Laura Beretta[1,*]

JHEP Reports **2024**. vol. 6 | 1–10

Check for updates

**Background & Aims:** The effectiveness of surveillance for hepatocellular carcinoma (HCC) in patients with cirrhosis is limited, due to inadequate risk stratification and suboptimal performance of current screening modalities.

**Methods:** We developed a multicenter prospective cohort of patients with cirrhosis undergoing surveillance with MRI and applied global untargeted metabolomics to 612 longitudinal serum samples from 203 patients. Among them, 37 developed HCC during follow-up.

**Results:** We identified 150 metabolites with significant abundance changes in samples collected prior to HCC (Cases) compared to samples from patients who did not develop HCC (Controls). Tauro-conjugated bile acids and gamma-glutamyl amino acids were increased, while acyl-cholines and deoxycholate derivatives were decreased. Seven amino acids including serine and alanine had strong associations with HCC risk, while strong protective effects were observed for N-acetylglycine and glycerophosphorylcholine. Machine learning using the 150 metabolites, age, gender, and *PNPLA3* and *TMS6SF2* single nucleotide polymorphisms, identified 15 variables giving optimal performance. Among them, N-acetylglycine had the highest AUC in discriminating Cases and Controls. When restricting Cases to samples collected within 1 year prior to HCC (Cases-12M), additional metabolites including microbiota-derived metabolites were identified. The combination of the top six variables identified by machine learning (alpha-fetoprotein, 6-bromotryptophan, N-acetylglycine, salicyluric glucuronide, testosterone sulfate and age) had good performance in discriminating Cases-12M from Controls (AUC 0.88, 95% CI 0.83-0.93). Finally, 23 metabolites distinguished Cases with LI-RADS-3 lesions from Controls with LI-RADS-3 lesions, with reduced abundance of acyl-cholines and glycerophosphorylcholine-related lysophospholipids in Cases.

**Conclusions:** This study identified N-acetylglycine, amino acids, bile acids and choline-derived metabolites as biomarkers of HCC risk, and microbiota-derived metabolites as contributors to HCC development.

© 2024 The Authors. Published by Elsevier B.V. on behalf of European Association for the Study of the Liver (EASL). This is an open access article under the CC BY license (http://creativecommons.org/licenses/by/4.0/).

## Introduction

Liver cancer is a major cause of death globally, and the number of people diagnosed with liver cancer is predicted to rise by 55% between 2020 and 2040.[1] In the United States, mortality rates for hepatocellular carcinoma (HCC) have started to decline, although HCC incidence continues to increase in over half of states, due in part to racial/ethnic disparity.[2,3] While the overall survival of patients with HCC has improved over recent decades, with increasing detection of localized HCC,[2] the overall prognosis of HCC remains dismal. To improve early diagnosis and access to potentially curative therapies, HCC surveillance based on semiannual liver ultrasound with or without serum alpha-fetoprotein (AFP) is recommended in patients with cirrhosis or chronic hepatitis B.[4] However, the effectiveness of HCC surveillance is limited by inadequate risk stratification and suboptimal performance of the current screening modalities for the detection of early-stage tumors.[5,6]

Promising new tools for HCC surveillance have been introduced in recent years. The detection rate of focal liver lesions is excellent with contrast-enhanced MRI, making it the best alternative to ultrasound for surveillance.[7,8] However, performing a full contrast-enhanced MRI for HCC surveillance in the currently defined risk populations is unrealistic due to availability and cost. Alternative imaging surveillance modalities include non-contrast MRI[9,10] and abbreviated MRI.[11–13] AFP remains the most widely used serum marker, despite its poor performance. Osteopontin has been identified as a promising serological biomarker for early detection of HCC and is highly complementary to AFP,[14–17] while proteome multimarker panels have also been proposed.[18–20] Meanwhile, cell-free DNA methylation patterns and extracellular vesicle markers have evolved as promising surveillance tools for early HCC detection in populations at risk.[21–27] Finally, algorithms and risk scores have also been developed. The performance of the

* Corresponding author. Address: Department of Molecular and Cellular Oncology, The University of Texas MD Anderson Cancer Center, Houston, TX, USA.
Tel: 713-792-9100.
*E-mail address:* lberetta@mdanderson.org (L. Beretta).
https://doi.org/10.1016/j.jhepr.2024.101119

GALAD score to detect early-stage HCC remains controversial.[28–30] Other scores, such as the HCC risk score aMAP and the Toronto HCC risk index (THRI), were developed to discriminate between low- and high-risk patients.[31–33] The combination of clinical and genetic predictors, such as patatin-like phospholipase domain-containing protein 3 (*PNPLA3*) and transmembrane 6 superfamily member 2 (*TM6SF2*) single nucleotide polymorphisms (SNPs), may improve HCC risk stratification.[34,35] Additional innovative approaches to non-invasively diagnose early HCC include gut and circulating targeted microbiota,[36,37] viral exposure signatures[38] and cell-free DNA fragmentomics features.[39]

Considering the rapidly changing epidemiology of HCC and the effects of etiology, age and gender on risk of HCC in cirrhosis, a "personalized" approach to surveillance is gaining growing support. In this context, novel biomarkers are needed both to stratify patients into low- and high-risk groups and to screen high-risk patients for detection of early-stage HCC. These biomarkers need to be evaluated in prospective cohorts of at-risk individuals under surveillance with imaging allowing for detection of small lesions and high sensitivity for early stage HCC. We therefore developed a multi-center prospective cohort of patients with cirrhosis undergoing surveillance with contrast-enhanced MRI and applied comprehensive metabolomics profiling to a large number of longitudinal samples collected from patients who developed or did not develop HCC during surveillance.

## Patients and methods

### Patient cohort

This study was conducted in accordance with the Declaration of Helsinki and approved by the Institutional review Board of all participating institutions. At recruitment, written informed consent was obtained from each participant. A nested group of 203 patients with cirrhosis under surveillance for HCC with contract-enhanced MRI were selected from a large multicenter prospective cohort initiated in 2017. The recruitment sites for this nested cohort study included Houston Methodist Hospital (Site 1: n = 108) and Baylor College of Medicine (Site 2: n = 95). All participants were followed during the duration of the study with contrast-enhanced MRI and blood collection, at time intervals defined by their surveillance standard of care at the participating institutions. The median duration of follow-up was 4.2 years, ranging from 1.9 to 4.8 years. Pre-HCC and HCC lesions were classified by the Liver Imaging Reporting and Data System (LI-RADS) classification system. Among the 203 patients, 38 developed liver cancer during follow-up (37 HCC and 1 cholangiocarcinoma). At enrollment and at each follow-up visit, one lavender and two red top venous blood tubes were collected from each patient. Serum, plasma and buffy coat aliquots were then stored in –80 °C. Detailed information on eligibility criteria, collected demographic and clinical parameters, HCC characteristics and secure data capture system are available in the Supplementary materials and methods.

### Global metabolomics profiling

A total of 612 serum samples (250 µl) collected from the 203 patients, with an average of three prospectively collected samples per patient and up to 11 samples, were submitted for global metabolomics profiling (Metabolon Inc, Durham, NC). The median time between successively collected samples was 5.9 months. Sample preparation and processing, and raw data analysis are described in detail in the Supplementary materials and methods.

### DNA extraction and SNP genotyping

Genomic DNA was extracted from 200 µl of buffy coat using the QIAamp DNA blood mini kit (Qiagen Co. Ltd., DE, Düsseldorf, Germany). *PNPLA3* rs738409 and *TM6SF2* rs58542926 were genotyped by TaqMan 5'-nuclease assays using predesigned TaqMan probes (Applied Biosystems, Foster City, CA), on a ViiA7 Real time PCR system (Applied Biosystems, Foster City, CA).

### Statistical analyses

All statistical analyses are described in detail in the Supplementary materials and methods.

## Results

### Nested cohort study of patients with cirrhosis under HCC surveillance by contrast MRI

In this study, we used a nested cohort of 203 patients selected from two sites in a multicenter prospective cohort of patients with cirrhosis under surveillance for HCC by contrast MRI. The demographic and clinical parameters of the study participants at enrollment are shown in Table S1. Males and females were equally distributed and most patients were non-Hispanic White. The median age (61), BMI (30.8) and presence of diabetes (45%) at recruitment were similar at both sites. The most common etiologies, MASLD (38%) and HCV (31%), were similarly observed in patients from both sites. Alcohol was an important etiology in patients from Site 1 (35%). A significant difference in Child Pugh class was observed between the two sites ($p$ <0.001), with a majority of class A (68%) at Site 2 and a majority of class B (66%) at Site 1. Among the 203 patients, 38 developed liver cancer during follow-up (37 HCC and 1 cholangiocarcinoma). Overall, patients who developed liver cancer were significantly older with a median age of 66 compared to 61, and were more likely to be diabetic (63% *vs.* 41%) (Table S1).

Clinical data, imaging and biospecimens were collected for a total of 612 visits between February 2017 and November 2021. Patient outcome was again reviewed in May 2023. Samples from the patient who developed cholangiocarcinoma were excluded for data analysis. Controls included 490 samples collected from 165 patients who never developed HCC. Cases included 88 pre-HCC samples collected from the 37 patients who developed HCC during follow-up. An additional group of 31 samples (Cases-T) were collected from seven of the patients with HCC, following treatment.

### Metabolite abundance changes associated with risk of HCC

Global metabolomics profiling was performed on all 612 serum samples collected from the 203 patients. A total of 1,263 metabolites were measured across the following super-pathways: Amino Acids (n = 227), Carbohydrates (n = 27), Cofactors and Vitamins (n = 44), Energy (n = 11), Lipids (n = 498), Nucleotides

(n = 43), Peptides (n = 51), Xenobiotics (n = 333) and Partially Characterized Molecules (n = 29) (Fig. S1A). For each super-pathway, the percentages of metabolites detected in 100% of the samples and in over 75% of the samples are described in Fig. S1B.

We used linear mixed-effects models, incorporating time to last visit, to identify metabolites with significant abundance changes in serum samples collected prior to diagnosis in patients who developed HCC during follow-up (Cases) to samples collected from patients who did not develop HCC (Controls). AFP was also added to this analysis. AFP abundance was significantly increased in Cases compared to Controls, and remained significant after adjusting for false discovery rate (FDR) (q = 0.004). In addition, the abundance of 64 metabolites was significantly increased while the abundance of 86 metabolites was significantly decreased (Table S2; Fig. 1A). Significance remained after adjusting for FDR for 16 of these 150 metabolites (Fig. 1A). The largest increase was observed for taurohyocholate (q = 041). Four other tauro-conjugated bile acids had increases similar or higher than AFP: taurocholate (*p* = 0.005), taurochenodeoxycholate (*p* = 0.010), taurochenodeoxycholic acid 3-sulfate (*p* = 0.012) and taurocholenate sulfate (*p* = 0.005). The bile acid glycohyocholate was also strongly increased in Cases (*p* = 0.013). Several gamma-glutamyl amino acids were elevated in Cases compared to Controls: gamma-glutamylmethionine, gamma-glutamyltyrosine, gamma-glutamyltryptophan, gamma-glutamylserine, gamma-glutamylphenylalanine, and gamma-glutamyl-alpha-lysine. The largest decreases were observed for derivatives of the bile acid deoxycholate: isoursodeoxycholate (q = 0.054), ursodeoxycholate (*p* = 0.025), chenodeoxycholic acid sulfate (1) (*p* = 0.002), and isoursodeoxycholate sulfate (1) (*p* = 0.015). The pathways Androgenic Steroids, Benzoate Metabolism and Fatty Acid Metabolism (Acyl Choline) were also enriched among metabolites significantly decreased in Cases compared to Controls. Spearman's correlation of all 150 metabolites associated with HCC (Fig. S2) identified three highly correlated clusters (r >0.9): 1) glycohyocholate and taurocholate; 2) 3-indoxyl sulfate and 6−hydroxyindole sulfate; and 3) stearoylcholine, linoleoylcholine, oleoylcholine, dihomo−linolenoyl−choline, palmitoylcholine and arachidonoylcholine (Fig. 1B). The strength of the association between abundance of the 150 metabolites or AFP with risk for HCC was further determined by logistic regression analysis, adjusting for age, gender and diabetes (Fig. 1C). While a strong association was observed for AFP (adjusted odds ratio [aOR] 4.11, 95% CI 2.45-6.87, *p* <0.001), eight metabolites including six amino acids had stronger or similar associations with HCC risk. The strongest associations were observed for serine (aOR 11.28, 95% CI 3.76-33.89, *p* <0.001), alanine (aOR 7.73, 95% CI 2.68-22.30, *p* <0.001) and gamma-glutamylserine (aOR 6.05, 95% CI 2.39-15.27, *p* <0.001). In contrast, the high abundance of four metabolites was associated with significantly reduced risk of HCC (Fig. 1C). The strongest protective effects were observed for N-acetylglycine (aOR 0.12, 95% CI 0.06-0.27, *p* <0.001) and glycerophosphorylcholine (GPC) (aOR 0.28, 95% CI 0.12-0.64, *p* = 0.003).

The inclusion of samples collected after HCC diagnosis and treatment for some of the patients who developed HCC during surveillance allowed us to determine whether any of the metabolites identified as significantly increased or decreased in Cases *vs.* Controls demonstrated a reverse phenotype after treatment (Supplementary results).

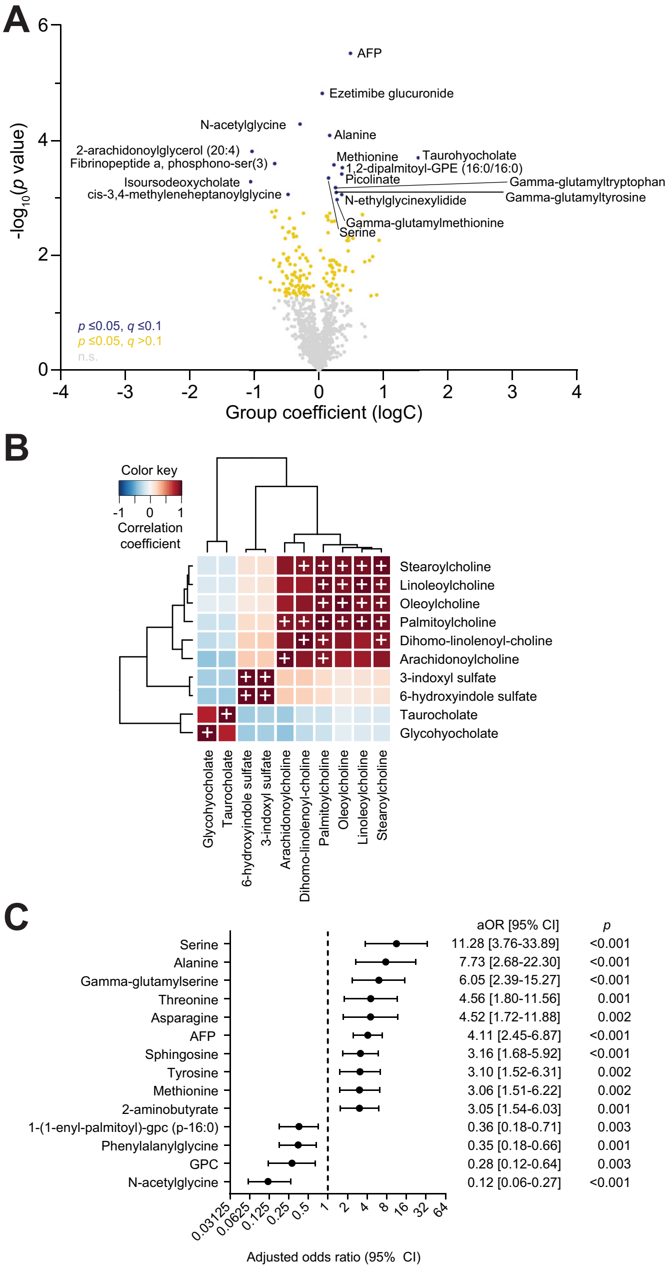


**Fig. 1. Metabolite abundance changes in Cases compared to Controls.** (A) Volcano plot for differential metabolites, between cases and controls. The significance and coefficient of group (Cases *vs.* Controls) as a fixed effect on metabolite abundance was determined by linear mixed-effects modeling. Group coefficients for log-transformed metabolite abundance (x-axis) and minus $\log_{10} p$ values (y-axis) are shown. Differential metabolites that remained significant after FDR adjustment (*p* ≤0.05, q ≤0.1) are shown in blue instead of yellow. (B) Spearman's correlation matrix of highly correlated clusters among the 150 differential metabolites. White cross: metabolite pairs with r >0.9 and *p* <0.05. (C) Forest plot depicting the aORs [95% CI] and *p* values determined by logistic regression, for selected metabolites associated with risk of HCC, after adjusting for age, gender and diabetes. AFP, alpha-fetoprotein; aOR, adjusted odds ratio; FDR, false discovery rate; GPC, glycerophosphocholine; HCC, hepatocellular carcinoma.

## Machine learning for HCC risk modeling

To determine which among AFP and the 150 metabolites associated with risk of HCC (Table S2) contributed the most to

risk prediction, the conditional inference random forest machine learning algorithm was implemented (Figs S3 and Fig. 2A). In this model, we wanted to also include age, gender and the genotypes of SNPs previously associated with HCC risk, so we genotyped *PNPLA3* rs738409 and *TMS6SF2* rs58542926 in all study participants (Fig. S4). The frequency of the *PNPLA3* rs738409 homozygous GG genotype was 21.6% in Cases and 18.2% in Controls. The frequency of the *TM6SF2* rs58542926 heterozygous CT genotype increased – but not significantly – in Cases (16.2%) compared to Controls (7.9%). The rare *TM6SF2* rs58542926 homozygous TT genotype was also detected in 2.7% and 2.4% of Cases and Controls, respectively. The conditional inference random forest model performed well in discriminating Cases and Controls (AUC 0.87, 95% CI 0.84-0.91). Based on permutation-based importance scores, age was the most important variable overall followed by AFP, testosterone sulfate and N-acetylglycine. The other demographic and genetic parameters, *TMS6SF2* rs58542926 CT/TT genotypes, *PNPLA3* rs738409 GG genotype, and gender were ranked 53th, 69st and 134st, respectively. To further determine best features of the model, we further performed feature selection by recursive feature elimination, which identified 15 variables giving optimal model performance (Fig. 2B). Among them, age and five metabolites had better individual AUCs than AFP (AUC 0.64, 95% CI 0.58-0.71), with the best AUC observed for N-acetylglycine (AUC 0.69, 95% CI 0.63-0.75) followed by age (AUC 0.68, 95% CI 0.62-0.74), palmitoloelylcholine (AUC 0.67, 95% CI 0.60-0.73), alanine (AUC 0.66, 95% CI 0.60-0.72), 1-(1-enyl-palmitoyl)-GPC (AUC 0.65, 95% CI 0.59-0.71) and picolinate (AUC 0.65, 95% CI 0.59-0.70). The top six variables were AFP, N-acetylglycine, age, alanine, taurocholenate sulfate and palmitoloelylcholine. Receiver-operating characteristic curve analysis using these top six variables showed improved performance in

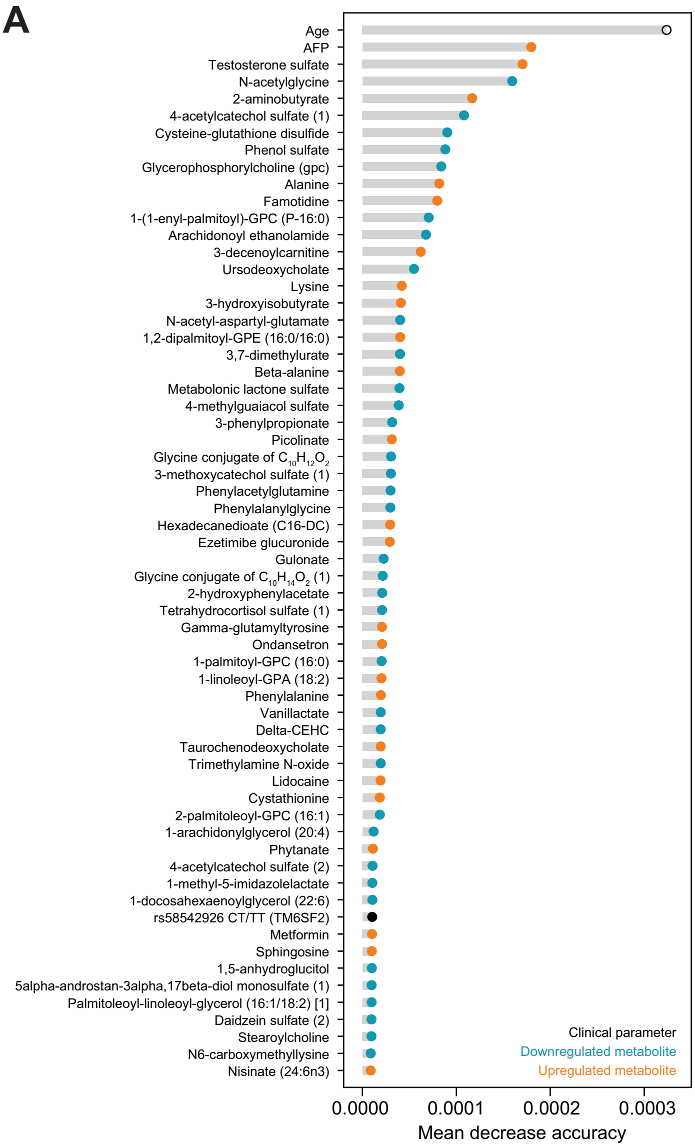


B

| Variable | AUC [95% CI] |
|---|---|
| Age | 0.68 [0.62-0.74] |
| N-acetylglycine | 0.69 [0.63-0.75] |
| AFP | 0.64 [0.58-0.71] |
| Taurocholenate sulfate | 0.63 [0.57-0.69] |
| Palmitoloelycholine | 0.67 [0.60-0.73] |
| Alanine | 0.66 [0.60-0.72] |
| 1,5-anhydroglucitol | 0.61 [0.55-0.67] |
| Ursodeoxycholate | 0.62 [0.55-0.69] |
| 1-(1-enyl-palmitoyl)-GPC (P-16:0) | 0.65 [0.59-0.71] |
| Picolinate | 0.65 [0.59-0.70] |
| Pristanate | 0.61 [0.54-0.67] |
| 4-acetylphenol sulfate | 0.64 [0.58-0.70] |
| 3-phenylpropionate | 0.58 [0.52-0.65] |
| Phenol sulfate | 0.59 [0.52-0.67] |
| Testosterone sulfate | 0.61 [0.56-0.67] |

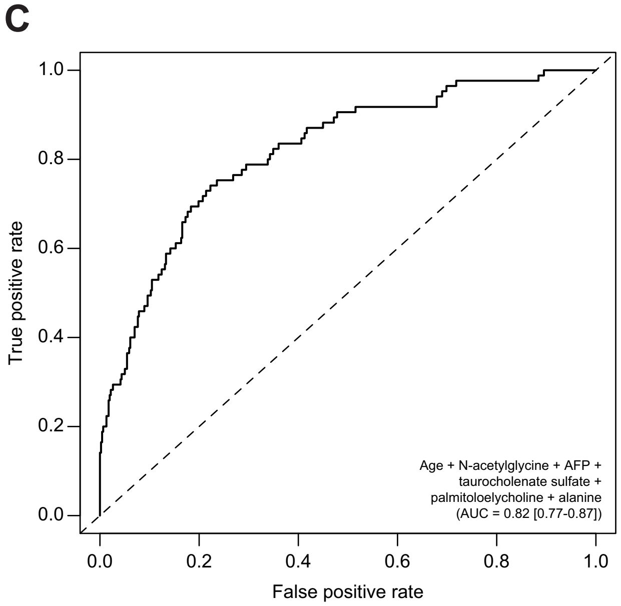


**Fig. 2. Modeling contribution of metabolites abundance, demographic and genetic parameters, in hepatocellular carcinoma prediction.** (A) Conditional inference random forest was implemented. Top variables, sorted by descending importance (Mean Decrease in Accuracy), are shown. (B) Following recursive feature elimination, 15 variables were identified to give optimal model performance. Individual AUCs are shown. (C) Receiver-operating characteristic curve and AUC (95% CI) for the combination of the top six markers comparing Cases to Controls. AFP, alpha-fetoprotein; GPC, glycerophosphocholine.

discriminating Cases from Controls (AUC 0.82, 95% CI 0.77-0.87) compared to AFP alone (Fig. 2C).

### Metabolite abundance changes within a year prior to HCC diagnosis and 1-year HCC risk modeling

We again used linear mixed-effects models, incorporating time to last visit, to identify metabolites with significant abundance changes in samples collected within 12 months prior to diagnosis in patients who developed HCC during follow-up (Cases-12M) to samples collected from patients who did not develop HCC (Controls). For comparison, we also used linear mixed-effects models incorporating time to last visit, to determine their abundance changes compared to Controls, in samples collected within 6 months (Cases-6M) or 24 months (Cases-24M) prior to HCC diagnosis (Table S3; Fig. S5). AFP was again added to this analysis. Ten metabolites, including the microbiota-derived metabolites salicyluric glucuronide and cinnamoylglycine, had significant abundance changes in Cases-12M but not in Cases-24M, compared to Controls, suggesting events occurring close to HCC development (Table S3; Fig. S5). AFP abundance was significantly increased in Cases-12M compared to Controls, remaining significant after adjusting for FDR, and with, as anticipated, a stronger increase than observed when comparing Cases to Controls ($q < 0.001$). The abundance of 62 metabolites was also significantly increased while the abundance of 30 metabolites was significantly decreased (Table S3; Fig. S5). Significance remained after adjusting for FDR for 13 of these 92 metabolites (Fig. S5). The largest increase was observed again for taurohyocholate ($p < 0.001$) and other tauro-conjugated bile acids had increases similar or higher than AFP: taurochenodeoxycholate ($p = 0.012$) and taurochenodeoxycholic acid 3-sulfate ($p = 0.019$). Again, the bile acid glycohyocholate was also strongly increased ($p = 0.015$). New metabolites were found strongly increased in Cases-12M compared to Controls: testosterone sulfate ($p = 0.003$) and salicyluric glucuronide ($p = 0.035$). Remarkably, for these two metabolites, the group coefficient further increased when comparing Cases-6M to Controls. The largest decreases in Cases-12M compared to Controls, were observed for iso-ursodeoxycholate ($p = 0.009$), as well as the microbiota-derived metabolites 3-phenylproprionate ($p = 0.001$) and cinnamoylglycine ($p = 0.049$). For these two microbiota-derived metabolites, the group coefficient further decreased in Cases-6M compared to Controls.

To determine which among AFP and the 92 metabolites associated with HCC diagnosis within 12 months of follow-up contributed the most to risk prediction, conditional inference random forest was again implemented, including age, gender, *PNPLA3* rs738409 GG, and *TM6SF2* rs58542926 CT/TT genotypes (Fig S6 and Fig. 3A). The conditional inference random forest model performed very well in discriminating Cases-12M

A

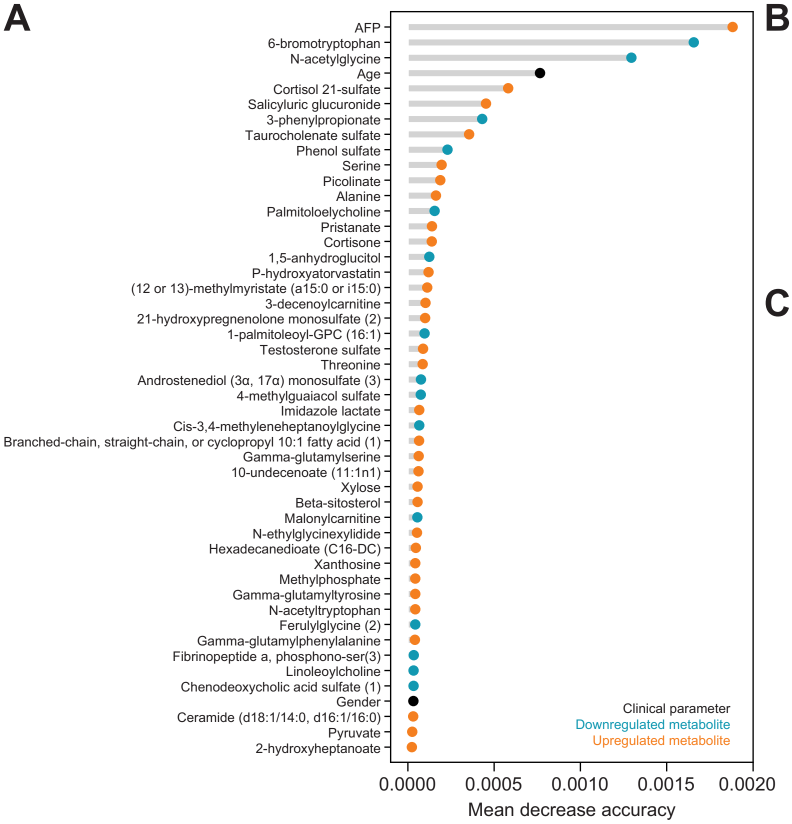


B

| Variable | AUC [95% CI] |
|---|---|
| N-acetylglycine | 0.72 [0.64-0.81] |
| AFP | 0.65 [0.56-0.75] |
| Salicyluric glucuronide | 0.62 [0.52-0.71] |
| 6-bromotryptophan | 0.70 [0.61-0.79] |
| Age | 0.69 [0.62-0.76] |
| Testosterone sulfate | 0.63 [0.55-0.71] |

C

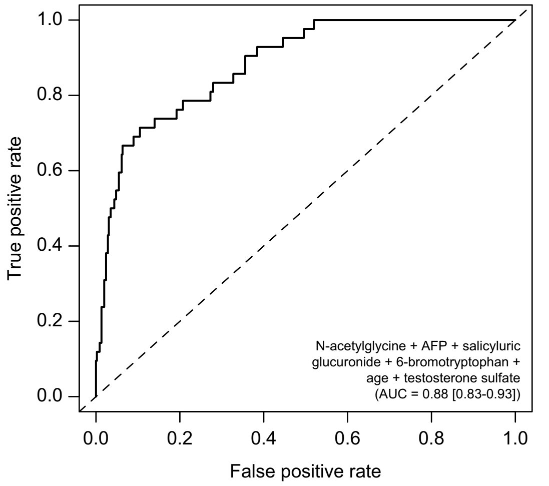


**Fig. 3. Modeling contribution of metabolites abundance, demographic and genetic parameters, in predicting hepatocellular carcinoma diagnosis within 12 months.** (A) Conditional inference random forest was implemented. Top variables, sorted by descending importance (Mean Decrease in Accuracy), are shown. (B) Following recursive feature elimination, six variables were identified to give optimal model performance. Individual AUCs are shown. (C) Receiver-operating characteristic curve and AUC (95% CI) for the combination of the six markers comparing Cases-12M to Controls. AFP, alpha-fetoprotein; GPC, glycerophosphocholine.

and Controls (AUC 0.92, 95% CI 0.87-0.96). AFP was the most important variable overall, followed by 6-bromotryptophan, N-acetylglycine and age. The other demographic and genetic parameters, gender, *PNPLA3* rs738409 GG, and *TMS6SF2* rs58542926 CT/TT were ranked 45th, 53rd and 65th, respectively. Feature selection by recursive feature elimination further identified six variables giving optimal model performance: N-acetylglycine, AFP, salicyluric glucuronide, 6-bromotryptophan, age and testosterone sulfate (Fig. 3B). Among them, N-acetylglycine (AUC 0.72, 95% CI 0.64-0.81), 6-bromotryptophan (AUC 0.70, 95% CI 0.61-0.79) and age (AUC 0.69, 95% CI 0.62-0.76) had better individual AUCs than AFP (AUC 0.65, 95% CI 0.56-0.75). Receiver-operating characteristic curve analysis using the six variables showed improved performance in discriminating Cases-12M from Controls (AUC 0.88, 95% CI 0.83-0.93) when compared to AFP alone (Fig. 3C).

### Clinical and demographic parameters affecting the identified HCC risk-associated metabolites

We evaluated whether clinical, demographic and genetic parameters were associated with abundance changes for the HCC-associated metabolites described in Table S2. In a redundancy analysis, the metabolites were used as response variables and age, gender, etiologies and *PNPLA3* rs738409 and *TM6SF2* rs58542926 SNPs as explanatory variables. The model was statistically significant ($p$ = 0.001, 9.21% variation explained) with the main contribution to variation observed for gender (2.93%, $p$ = 0.001). The contribution of the two SNPs was significant although modest (*TM6SF2*: 0.59%, $p$ = 0.002; *PNPLA3*: 0.47%, $p$ = 0.002) (Fig. 4A). Linear mixed-effects analysis identified metabolites significantly affected by gender, *TM6SF2,* and *PNPLA3* (Table S4). The top three metabolites affected by *PNPLA3* were involved in secondary bile acid metabolism, while the top three metabolites affected by gender were related to androgenic steroids. Redundancy analysis ordination plots showed the association between select metabolites and gender, *TM6SF2*, and *PNPLA3* (Fig. 4B). Testosterone sulfate was associated with male gender ($p$ <0.001), while isoursodeoxycholate sulfate ($p$ = 0.005), ursodeoxycholate ($p$ = 0.034), isoursodeoxycholate ($p$ = 0.045), 1-arachidonylglycerol (20:4) ($p$ <0.001), and 1-docosahexaenoylglycerol ($p$ <0.001) were associated with female gender. Androstenediol (3beta,17beta) disulfate ($p$ = 0.003), 5alpha-androstan-3beta,17beta-diol disulfate ($p$ = 0.024), and 5alpha-androstan-3alpha,17beta-diol monosulfate ($p$ = 0.033) were strongly negatively associated with the *TM6SF2* risk allele T. The acyl-cholines arachidonoylcholine ($p$ = 0.015) and dihomo-linolenoyl-choline ($p$ = 0.034), and lysophospholipid 1-arachidonoyl-GPC (20:4n6) ($p$ = 0.006) were negatively associated with the *PNPLA3* risk allele G, although weakly.

### Distinguishing cases with LI-RADS-3 lesions from controls with LI-RADS-3 lesions

Finally, we tested whether any of the HCC-associated metabolites identified (Table S2) could distinguish between Cases with LI-RADS-3 lesions who developed HCC during follow-up (Cases-LR3) and Controls with LI-RADS-3 lesions who did not develop HCC during follow-up (Controls-LR3). A total of 33 samples from Controls and 15 samples from Cases had a paired MRI showing detection of a single (n = 34), two (n = 11) or three (n = 3) LI-RADS-3 lesions, without detection of any other lesion. In addition to AFP, the abundance of nine metabolites was significantly increased in Cases-LR3, while the abundance of 13 metabolites was significantly decreased (Table S5). 1,2-dilinoleoyl-GPE (18:2/18:2) ($p$ = 0.02) and malonate ($p$ = 0.007) had the greatest increases, while phenolsulfate ($p$ = 0.02) had the greatest decrease, in Cases-LR3 compared to Controls-LR3. Principal component analysis plots based on levels of these 23 metabolites showed a clear separation between Cases-LR3 and Controls-LR3 (PERMANOVA $R^2$=0.157, $p$ = 0.001) (Fig. 5A). Interestingly, the abundance of four acyl-cholines was strongly reduced in Cases-LR3 compared to Controls-LR3: dihomo-linolenoyl-choline ($p$ = 0.001), arachidonoylcholine ($p$ = 0.006), docosahexaenoylcholine ($p$ = 0.037), and palmitoylcholine ($p$ = 0.031) (Fig. 5B). GPC and GPC-related lysophospholipids were also significantly reduced in Cases-LR3 compared to Controls-LR3: GPC ($p$ = 0.011), 1-(1-enyl-palmitoyl)-GPC (P-16:0) ($p$ <0.001), 1-palmitoyl GPC (16:0) ($p$ = 0.006) and 1-arachidonoyl-GPC (20:4n6) ($p$ = 0.005) (Fig. 5C).

## Discussion

The identification of key metabolites that are associated with HCC risk in patients with cirrhosis can expand our knowledge on metabolic reprogramming leading to HCC development, and identify biomarkers for detection of early-stage HCC. To date, only a handful of metabolomics studies in HCC have been reported, including the study from Lewinska *et al.* demonstrating that a panel of serum metabolites outperformed AFP in distinguishing patients with MASLD-HCC from those without HCC.[40] However, no metabolic studies have been performed on longitudinal biospecimens from a prospective cohort of high-risk patients with cirrhosis under surveillance for HCC. Furthermore, the multicenter prospective cohort of patients with cirrhosis we developed was based on surveillance by contrast-enhanced MRI. Such a cohort provides a unique opportunity to study blood biomarkers and imaging features on clinical material from patients rigorously classified as having very early-stage disease in a surveillance setting. The LI-RADS classification system, initially released in 2011, allows for HCC diagnosis with CT or MRI with extracellular contrast agents, as well as for pre-HCC lesion characterization.[41] Longitudinal collection of paired blood samples and MRI images from patients with cirrhosis is particularly valuable in assessing how early blood and imaging markers become positive during the period when lesions are observed to obtain a diagnosis of HCC.

The use of untargeted metabolomics is an attractive method for biomarker discovery, although there are still limitations with this method. Unlike targeted metabolomics, untargeted metabolomics is unable to provide absolute quantification and is sensitive to sample preparation and analytical method.[42,43]

An important finding of our study is the identification of several microbiome-related metabolites, most remarkably in the year prior to HCC diagnosis, suggesting a potential direct effect of these metabolites in HCC development. Patients with HCC have a microbiome signature distinct from non-HCC controls, and evidence supports the critical role of the gut microbiome and its metabolites in influencing immune and metabolic events associated with HCC development.[44] Bile

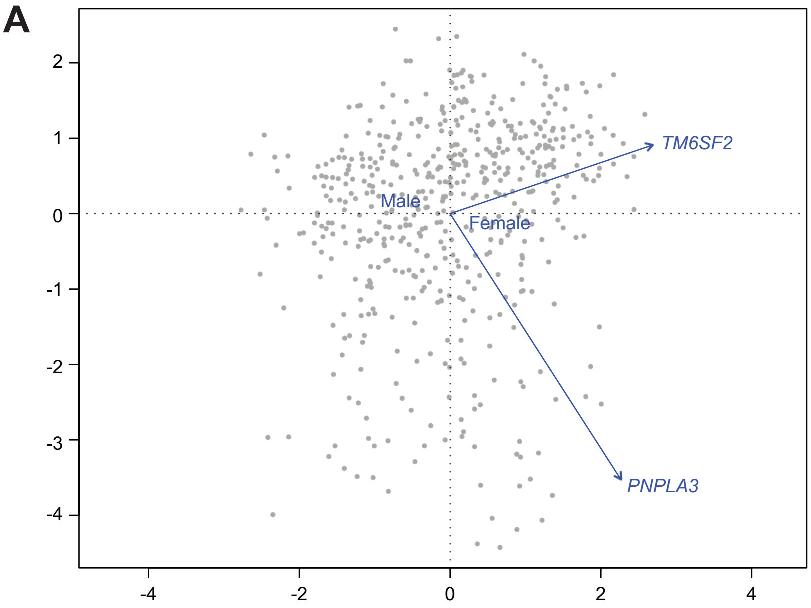


| | *p* | % Variance explained |
|---|---|---|
| **Anova-like test** | | |
| Model | 0.001 | 3.44% |
| axis 1 | 0.003 | 1.00% |
| axis 2 | 0.041 | 0.67% |
| **Marginal test** | | |
| Gender | 0.001 | 2.93% |
| *PNPLA3* | 0.002 | 0.47% |
| *TM6SF2* | 0.002 | 0.59% |

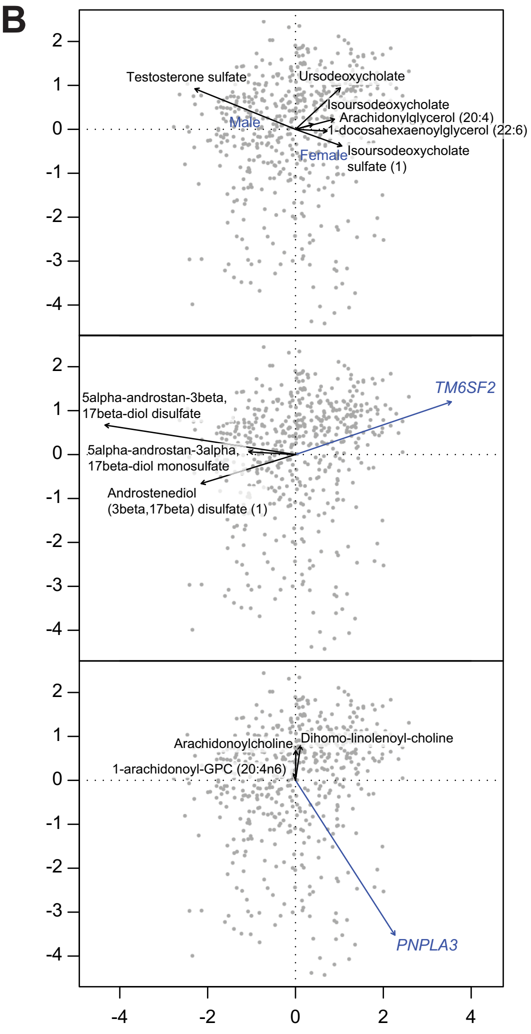


**Fig. 4. Relationship between demographic, clinical and genetic parameters and metabolites associated with hepatocellular carcinoma risk.** (A) Redundancy analysis was conducted to determine the relationship between selected parameters (explanatory variables) and HCC-associated metabolite profile (response variables). The explanatory variables with the stronger effects are shown, together with ANOVA-like significance test of the model, and marginal test of the explanatory variables. (B) Redundancy analysis plots showing the relationship between gender, *TM6SF2* rs58542926 and *PNPLA3* rs738409 and selected metabolites. HCC, hepatocellular carcinoma.

acids have been used in panels to discriminate HCC from cirrhosis.[45] We observed a positive association between HCC risk and the primary taurine-conjugated bile acids and their sulfated derivatives, as well as conjugated forms of the rare bile acid hyocholate. Conversely, we observed that HCC risk was associated with a decrease in the secondary bile acid ursodeoxycholate and its derivatives. While primary bile acids are synthesized and conjugated in the liver, secondary bile acids are derived from metabolism of primary bile acids by gut microbes. Ursodeoxycholate, a non-toxic hydrophilic bile acid used in the treatment of cholestatic liver disease, exhibits protective effects against HCC.[46] Circulating levels of isoursodeoxycholate are mostly determined by the gut microbiome.[47] We also observed an association between HCC risk and multiple amino acids. Notably, HCC risk was strongly associated with elevated levels of alanine and with low levels of several phenylalanine/tyrosine derivatives, including 2-hydroxyphenylacetate and microbiota-derived 3-phenylpropionate, phenylacetylglutamine, and phenol sulfate. Serum levels of 3-phenylpropionate and cinnamoylglycine have been associated with higher gut microbiome diversity and intestinal barrier function.[48,49]

The strongest negative association with HCC risk was observed for N-acetylglycine. N-acetylglycine outperformed AFP in all patient group comparisons performed. N-acetylglycine, a derivative of glycine, has been associated with protection against obesity and obesity-related diseases.[50] Low levels of N-acetylglycine have been shown to mediate gut microbiome-dependent smoking cessation-induced weight gain.[51] Supplementation of high-fat diet-fed mice with N-acetylglycine led to lower levels of the adiposity-related Trem2$^{+}$ macrophages, and altered signaling of multiple pathways in adipose immune cells. N-acetylglycine may therefore have protective effects on HCC by modulating obesity-related immunity.[51]

Another important finding of our study is the identification of several choline-derived metabolites, mostly acyl-cholines and GPC-derived metabolites. The serum abundance of these metabolites was significantly reduced in patients with LI-RADS-3 lesions who developed HCC during follow-up, compared to

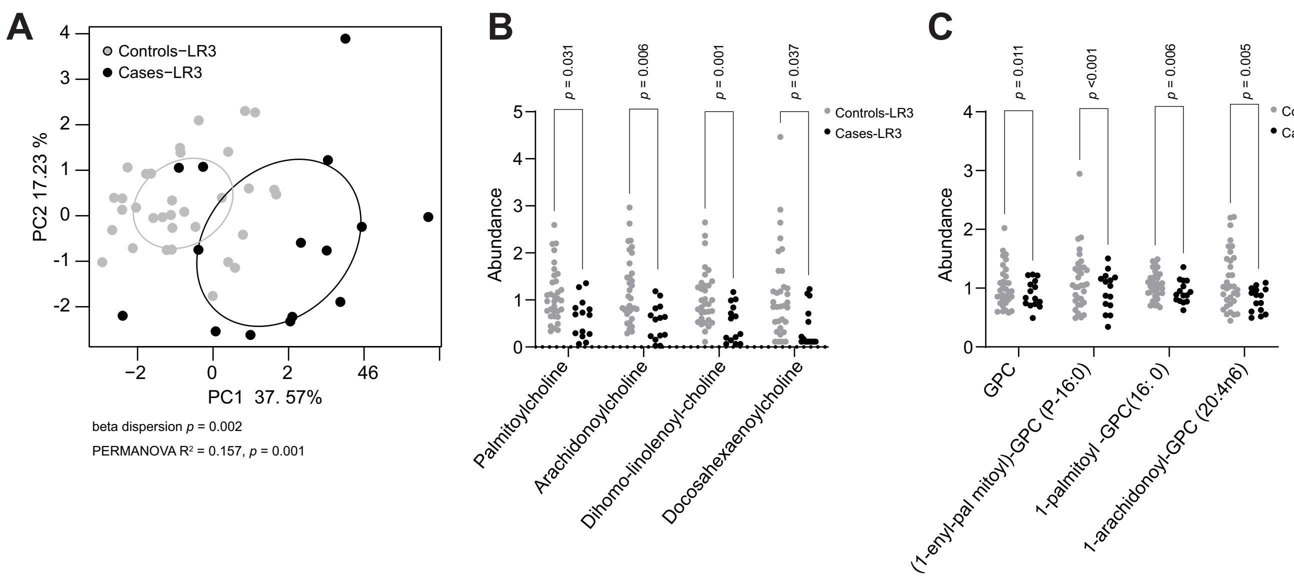


**Fig. 5. Metabolite profiles distinguishing Cases-LR3 and Controls-LR3.** (A) PCA plot of 23 metabolites with significant differences in Cases-LR3 *vs*. Controls-LR3, as determined by linear mixed-effects modeling ($p$ <0.05 for group as a fixed effect). (B–C) Plots showing the significant differences between Cases-LR3 and Controls-LR3 in (B) 4 acyl-cholines and, (C) GPC and GPC-derived metabolites. Significance was determined by linear mixed-effects modeling. GPC, glycerophosphocholine; PCA, principal component analysis.

patients with LI-RADS-3 lesions who did not develop HCC during follow-up. GPC is a precursor of endogenous choline and whether reduction of GPC derivatives results in reduced choline in the liver prior to HCC development should be investigated. In mice, deficiency of the lysophospholipase PNPLA7, markedly decreases hepatic GPC, choline, and several metabolites related to choline/methionine metabolism.[52] While in our study, the *PNPLA3* SNP had only weak associations with both HCC risk and overall HCC-associated metabolite changes, the two acyl-cholines arachidonoylcholine and dihomo-linolenoyl-choline, and the lysophospholipid 1-arachidonoyl-GPC (20:4n6) were negatively associated with the *PNPLA3* rs738409 risk allele G.

Finally, AFP showed good performance even years before HCC diagnosis in agreement with a prior report that serum AFP levels increase more than 10 years before detection of HCC.[53] This suggests that AFP could be used together with some of the metabolites identified, to assign patients to high-risk *vs.* low-risk groups for surveillance, helping the field move toward precision surveillance, where surveillance tests and intervals are tailored to individual HCC risk.

Overall, N-acetylglycine, amino acids, bile acids and choline-derived metabolites were identified as biomarkers to better identity patients with cirrhosis at high risk of HCC. Such biomarkers may significantly improve early-stage HCC detection for patients undergoing HCC surveillance, a critical step to increasing curative treatment opportunities and reducing mortality. This study also demonstrated the critical role of the gut microbiome in the progression of cirrhosis to HCC, in particular within the year prior to HCC development. Modulation of these microbiome-related metabolites by gut microbiome-targeted therapies is therefore an attractive potential strategy for reducing HCC risk.


## Affiliations

[1]*Department of Molecular and Cellular Oncology, The University of Texas MD Anderson Cancer Center, Houston, TX, USA;* [2]*Margaret M. and Albert B. Alkek Department of Medicine, Section of Gastroenterology and Hepatology, Baylor College of Medicine, Houston, TX, USA;* [3]*Department of Gastroenterology, Houston Methodist Hospital, Houston, TX, USA;* [4]*Department of Biostatistics, The University of Texas MD Anderson Cancer Center, Houston, TX, USA*


## Abbreviations

AFP, alpha-fetoprotein; aOR, adjusted odds ratio; FDR, false discovery rate; GPC, glycerophosphorylcholine; HCC, hepatocellular carcinoma; LI-RADS, Liver Imaging Reporting and Data System; MASLD, metabolic dysfunction-associated steatotic liver disease; PNPLA3, patatin-like phospholipase domain-containing protein 3; SNP, single nucleotide polymorphism; TM6SF2, transmembrane 6 superfamily member 2.


## Financial support

This study was supported by National Institute of Health/National Cancer Institute R01 CA195524 to L.B.


## Conflict of interest

Dr. John Vierling is a scientific advisor to Bristol Myers Squibb, Merck, Novartis, LabCorp and Genentech.

Please refer to the accompanying ICMJE disclosure forms for further details.

## Authors' contributions

Conceptualization, LB; Methodology, SYK, TLC, JMV, DWV, LB; Formal Analysis, JIS, ACF, SYK, CIS, PW; Investigation, JIS, CIS, TLC; Resources, JL, AE, DWC, JMV, DWV; Writing-Original Draft, JIS, ACF, SYK, LB; Visualization, CIS, ACF, SYK; Supervision, LB; Project Administration, TLC; Funding Acquisition, L.B.

## Data availability statement

The metabolomics data have been uploaded to the MetaboLights database (https://www.ebi.ac.uk/metabolights), under study accession number MTBLS8764.

## Supplementary data

Supplementary data to this article can be found online at https://doi.org/10.1016/j.jhepr.2024.101119.

## References

*Author names in bold designate shared co-first authorship*


[1] Rumgay H, Arnold M, Ferlay J, et al. Global burden of primary liver cancer in 2020 and predictions to 2040. J Hepatol 2022;77:1598–1606.

[2] Lee YT, Wang JJ, Luu M, et al. The mortality and overall survival trends of primary liver cancer in the United States. J Natl Cancer Inst 2021;113:1531–1541.

[3] Alvarez CS, Petrick JL, Parisi D, et al. Racial/ethnic disparities in hepatocellular carcinoma incidence and mortality rates in the United States, 1992-2018. Hepatology 2022;76:589–598.

[4] Asrani SK, Ghabril MS, Kuo A, et al. Quality measures in HCC care by the practice metrics committee of the American association for the study of liver diseases. Hepatology 2022;75:1289–1299.

[5] **Singal AG**, **Sanduzzi-Zamparelli M**, Nahon P, et al. International Liver Cancer Association (ILCA) white paper on hepatocellular carcinoma risk stratification and surveillance. J Hepatol 2023;79:226–239.

[6] Lee HM, Lidofsky SD, Taddei TH, et al. Attacking the public health crisis of hepatocellular carcinoma at its roots. Hepatology 2023;77:1456–1459.

[7] **Kim HL**, **An J**, Park JA, et al. Magnetic resonance imaging is cost-effective for hepatocellular carcinoma surveillance in high-risk patients with cirrhosis. Hepatology 2019;69:1599–1613.

[8] Nahon P, Najean M, Layese R, et al. Early hepatocellular carcinoma detection using magnetic resonance imaging is cost-effective in high-risk patients with cirrhosis. JHEP Rep 2022;4:100390.

[9] Chan MV, Huo YR, Trieu N, et al. Noncontrast MRI for hepatocellular carcinoma detection: a systematic review and meta-analysis - a potential surveillance tool? Clin Gastroenterol Hepatol 2022;20:44–56.e42.

[10] **Park HJ**, **Jang HY**, Kim SY, et al. Non-enhanced magnetic resonance imaging as a surveillance tool for hepatocellular carcinoma: comparison with ultrasound. J Hepatol 2020;72:718–724.

[11] Gupta P, Soundararajan R, Patel A, et al. Abbreviated MRI for hepatocellular carcinoma screening: a systematic review and meta-analysis. J Hepatol 2021;75:108–119.

[12] Yokoo T, Masaki N, Parikh ND, et al. Multicenter validation of abbreviated MRI for detecting early-stage hepatocellular carcinoma. Radiology 2023;307:e220917.

[13] Ronot M, Nahon P, Rimola J. Screening of liver cancer with abbreviated MRI. Hepatology 2023;78:670–686.

[14] Shang S, Plymoth A, Ge S, et al. Identification of osteopontin as a novel marker for early hepatocellular carcinoma. Hepatology 2012;55:483–490.

[15] Duarte-Salles T, Misra S, Stepien M, et al. Circulating osteopontin and prediction of hepatocellular carcinoma development in a large European population. Cancer Prev Res (Phila) 2016;9:758–765.

[16] Khan IM, Gjuka D, Jiao J, et al. A novel biomarker panel for the early detection and risk assessment of hepatocellular carcinoma in patients with cirrhosis. Cancer Prev Res (Phila) 2021;14:667–674.

[17] **Zhu M**, **Zheng J**, **Wu F**, et al. OPN is a promising serological biomarker for hepatocellular carcinoma diagnosis. J Med Virol 2020;92(12):3596–3603.

[18] **Yeo I**, **Kim GA**, **Kim H**, et al. Proteome multimarker panel with multiple reaction monitoring-mass spectrometry for early detection of hepatocellular carcinoma. Hepatol Commun 2020;4:753–768.

[19] Poynard T, Lacombe JM, Deckmyn O, et al. External validation of LCR1-LCR2, a multivariable HCC risk calculator, in patients with chronic HCV. JHEP Rep 2021;3:100298.

[20] Piratvisuth T, Tanwandee T, Thongsawat S, et al. Multimarker panels for detection of early stage hepatocellular carcinoma: a prospective, multicenter, case-control study. Hepatol Commun 2022;6:679–691.

[21] Chalasani NP, Porter K, Bhattacharya A, et al. Validation of a novel multitarget blood test shows high sensitivity to detect early stage hepatocellular carcinoma. Clin Gastroenterol Hepatol 2022;20:173–182.e177.

[22] **Lin N**, **Lin Y**, **Xu J**, et al. A multi-analyte cell-free DNA-based blood test for early detection of hepatocellular carcinoma. Hepatol Commun 2022;6:1753–1763.

[23] Singal AG, Haaland B, Parikh ND, et al. Comparison of a multitarget blood test to ultrasound and alpha-fetoprotein for hepatocellular carcinoma surveillance: results of a network meta-analysis. Hepatol Commun 2022;6:2925–2936.

[24] Chhatwal J, Samur S, Yang JD, et al. Effectiveness of HCC surveillance programs using multitarget blood test: a modeling study. Hepatol Commun 2023;7.

[25] Fu S, Debes JD, Boonstra A. DNA methylation markers in the detection of hepatocellular carcinoma. Eur J Cancer 2023;191:112960.

[26] Sanchez JI, Jiao J, Kwan SY, et al. Lipidomic profiles of plasma exosomes identify candidate biomarkers for early detection of hepatocellular carcinoma in patients with cirrhosis. Cancer Prev Res (Phila) 2021;14:955–962.

[27] Sun N, Zhang C, Lee YT, et al. HCC EV ECG score: an extracellular vesicle-based protein assay for detection of early-stage hepatocellular carcinoma. Hepatology 2023;77:774–788.

[28] Best J, Bechmann LP, Sowa JP, et al. GALAD score detects early hepatocellular carcinoma in an international cohort of patients with nonalcoholic steatohepatitis. Clin Gastroenterol Hepatol 2020;18:728–735 e724.

[29] Singal AG, Tayob N, Mehta A, et al. GALAD demonstrates high sensitivity for HCC surveillance in a cohort of patients with cirrhosis. Hepatology 2022;75:541–549.

[30] Tayob N, Kanwal F, Alsarraj A, et al. The performance of AFP, AFP-3, DCP as biomarkers for detection of hepatocellular carcinoma (HCC): a phase 3 biomarker study in the United States. Clin Gastroenterol Hepatol 2023;21:415–423.e414.

[31] **Fan R**, **Papatheodoridis G**, **Sun J**, et al. aMAP risk score predicts hepatocellular carcinoma development in patients with chronic hepatitis. J Hepatol 2020;73:1368–1378.

[32] Åström H, Ndegwa N, Hagström H. External validation of the Toronto hepatocellular carcinoma risk index in a Swedish population. JHEP Rep 2021;3:100343.

[33] Johnson PJ, Innes H, Hughes DM, et al. Evaluation of the aMAP score for hepatocellular carcinoma surveillance: a realistic opportunity to risk stratify. Br J Cancer 2022;127:1263–1269.

[34] Nahon P, Bamba-Funck J, Layese R, et al. Integrating genetic variants into clinical models for hepatocellular carcinoma risk stratification in cirrhosis. J Hepatol 2023;78:584–595.

[35] Degasperi E, Galmozzi E, Pelusi S, et al. Hepatic fat-genetic risk score predicts hepatocellular carcinoma in patients with cirrhotic HCV treated with DAAs. Hepatology 2020;72:1912–1923.

[36] **Ren Z**, **Li A**, **Jiang J**, et al. Gut microbiome analysis as a tool towards targeted non-invasive biomarkers for early hepatocellular carcinoma. Gut 2019;68:1014–1023.

[37] **Cho EJ**, **Leem S**, Kim SA, et al. Circulating microbiota-based metagenomic signature for detection of hepatocellular carcinoma. Scientific Rep 2019;9:7536.

[38] **Liu J**, **Tang W**, Budhu A, et al. A viral exposure signature defines early onset of hepatocellular carcinoma. Cell 2020;182:317–328.e310.

[39] **Fan R**, **Chen L**, **Zhao S**, Novel, et al. high accuracy models for hepatocellular carcinoma prediction based on longitudinal data and cell-free DNA signatures. J Hepatol 2023;79(4):933–944.

[40] Lewinska M, Santos-Laso A, Arretxe E, et al. The altered serum lipidome and its diagnostic potential for Non-Alcoholic Fatty Liver (NAFL)-associated hepatocellular carcinoma. EBioMedicine 2021;73:103661.

[41] Chernyak V, Fowler KJ, Do RKG, et al. LI-RADS: looking back, looking forward. Radiology 2023;307:e222801.

[42] Schrimpe-Rutledge AC, Codreanu SG, Sherrod SD, et al. Untargeted metabolomics strategies-challenges and emerging directions. J Am Soc Mass Spectrom 2016;27:1897–1905.

[43] Lin YP, Caldwell GW, Li Y, et al. Inter-laboratory reproducibility of an untargeted metabolomics GC-MS assay for analysis of human plasma. Sci Rep 2020;10.

[44] Rajapakse J, Khatiwada S, Akon AC, et al. Unveiling the complex relationship between gut microbiota and liver cancer: opportunities for novel therapeutic interventions. Gut Microbes 2023;15:2240031.

[45] **Han J**, **Qin WX**, **Li ZL**, et al. Tissue and serum metabolite profiling reveals potential biomarkers of human hepatocellular carcinoma. Clin Chim Acta 2019;488:68–75.

[46] Luo W, Guo S, Zhou Y, et al. Hepatocellular carcinoma: novel understandings and therapeutic strategies based on bile acids (Review). Int J Oncol 2022;61.

[47] Louca P, Meijnikman AS, Nogal A, et al. The secondary bile acid isoursodeoxycholate correlates with post-prandial lipemia, inflammation, and appetite and changes post-bariatric surgery. Cell Rep Med 2023;4:100993.

[48] Menni C, Zhu J, Le Roy CI, et al. Serum metabolites reflecting gut microbiome alpha diversity predict type 2 diabetes. Gut Microbes 2020;11:1632–1642.

[49] Hu J, Chen J, Xu X, et al. Gut microbiota-derived 3-phenylpropionic acid promotes intestinal epithelial barrier function via AhR signaling. Microbiome 2023;11:102.

[50] **Su KJ**, **Chen XY**, Gong R, et al. Systematic metabolomic studies identified adult adiposity biomarkers with acetylglycine associated with fat loss in vivo. Front Mol Biosci 2023;10:1166333.

[51] **Fluhr L**, **Mor U**, Kolodziejczyk AA, et al. Gut microbiota modulates weight gain in mice after discontinued smoke exposure. Nature 2021;600:713–719.
[52] Harada S, Taketomi Y, Aiba T, et al. The lysophospholipase PNPLA7 controls hepatic choline and methionine metabolism. Biomolecules 2023;13.
[53] Hughes DM, Berhane S, Emily de Groot CA, et al. Serum levels of α-fetoprotein increased more than 10 Years before detection of hepatocellular carcinoma. Clin Gastroenterol Hepatol 2021;19:162–170.e164.

Keywords: Hepatocellular carcinoma; Cirrhosis; Surveillance; Biomarkers; Metabolomics.
*Received 23 February 2024; received in revised form 1 May 2024; accepted 8 May 2024; Available online 15 May 2024*

Journal of Hepatology, Volume *6*

## Supplemental information

# Metabolomics biomarkers of hepatocellular carcinoma in a prospective cohort of patients with cirrhosis

**Jessica I. Sanchez, Antoine C. Fontillas, Suet-Ying Kwan, Caren I. Sanchez, Tiffany L. Calderone, Jana L. Lee, Ahmed Elsaiey, Darrel W. Cleere, Peng Wei, John M. Vierling, David W. Victor, and Laura Beretta**

# Metabolomics biomarkers of hepatocellular carcinoma in a prospective cohort of patients with cirrhosis

Jessica I. Sanchez, Antoine C. Fontillas, Suet-Ying Kwan, Caren I. Sanchez, Tiffany L. Calderone, Jana L. Lee, Ahmed Elsaiey, Darrel W. Cleere, Peng Wei, John M. Vierling, David W. Victor, Laura Beretta

Table of contents

Supplementary materials and methods…………………………………………………………2

Supplementary figures……………………………………………………………………………8

Supplementary tables……………………………………………………………………………16

Supplementary results…………………………………………………………………………...29

## Supplementary materials and methods

### Patients Cohort

Medical history was collected to confirm eligibility (above 18 years of age, confirmed diagnosis of cirrhosis, no clinical evidence of significant hepatic decompensation). Cirrhosis was diagnosed using composite clinical, biochemical, hematological, imaging or histological criteria. Date of birth, gender, race, ethnicity, liver cirrhosis etiology, ascites status, encephalopathy status, Child Pugh score, Child Pugh class, Model for end stage liver disease (MELD) score, year of cirrhosis diagnosis, diabetes and date of diabetes onset were collected. CT was performed instead of MRI for 11 patients and 17 additional patients received a CT scan at one of the visits while receiving MRI for the remaining visits. Five of the 37 patients who developed HCC, presented with multifocal disease with the largest lesion ranging from 2.0 to 5.3cm (median=2.1cm). For the remaining 32 patients, the large majority presented with a small single lesion (median=2.1cm), with only 2 patients with a lesion larger than 4.0cm. Among the seven patients followed after treatment, four received transarterial chemoembolization alone, one received atezolizumab plus bevacizumab, one patient received transarterial chemoembolization followed by ablation and a liver transplant, and the last patient received Y90 radioembolization followed by a liver transplant. The median time of sample collection after treatment was 6.2 months.

Collected blood samples were kept at 4°C until processed, within 4 hours of collection. Serum, plasma and buffy coat aliquots were then stored in -80°C. The following clinical data were also collected at baseline and at each follow-up visit using electronic medical records: alpha-fetoprotein (AFP), alanine aminotransferase (ALT), aspartate aminotransferase (AST), alkaline phosphatase (ALP), total protein, albumin, total bilirubin, bilirubin direct, platelets, body mass index (BMI). All clinical data were entered into the study data capture system as an integrated part of a secure website supported by the Data Management Services team at MD Anderson Cancer Center. The secure website also provides an interface

to upload the related de-identified image files and documentation to a secure dedicated server space.

**Global Metabolomics Profiling**

Samples were prepared using the automated MicroLab STAR® system (Hamilton Company). For quality control (QC) purposes, several recovery standards were added prior to the first step in the extraction. Proteins were precipitated with methanol under vigorous shaking for 2 minutes (Glen Mills GrnoGrinder 2000) followed by centrifugation. The resulting extract was divided into five fractions: two for analysis by two separate reverse phase (RP)/UPLC-MS/MS methods with positive ion mode electrospray ionization (ESI), one for analysis by RP/UPLC-MS/MS with negative ion mode ESI, one for analysis by HILIC/UPLC-MS/MS with negative ion mode ESI, and one sample was reserved for backup.

Samples were briefly placed on a TurboVap® (Zymark) to remove the organic solvent. The sample extracts were stored overnight under nitrogen before preparation for analysis. Several types of controls were analyzed with the experimental samples: a pooled matrix sample generated by taking a small volume of each experimental sample served as a technical replicate throughout the data set; extracted water samples served as process blanks; and a cocktail of QC standards. Instrument variability was determined by calculating the median relative standard deviation (RSD) for the standards that were added to each sample prior to injection into the mass spectrometers. Overall process variability was determined by calculating the median RSD for all endogenous metabolites present in 100% of the pooled matrix samples. Experimental samples were randomized across the platform run with QC samples spaced evenly among the injections. All methods utilized a Waters ACQUITY ultra-performance liquid chromatography (UPLC) and a Thermo Scientific Q-Exactive high resolution/accurate mass spectrometer interfaced with a heated electrospray ionization (HESI-II) source and Orbitrap mass analyzer operated at 35,000 mass resolution. The sample extract was dried then reconstituted in solvents compatible to each of the four methods. Each reconstitution solvent contained a series of standards at fixed concentrations to ensure injection and

chromatographic consistency. One aliquot was analyzed using acidic positive ion conditions, chromatographically optimized for more hydrophilic compounds. In this method, the extract was gradient eluted from a C18 column (Waters UPLC BEH C18-2.1x100 mm, 1.7 µm) using water and methanol, containing 0.05% perfluoropentanoic acid (PFPA) and 0.1% formic acid (FA). Another aliquot was also analyzed using acidic positive ion conditions, however it was chromatographically optimized for more hydrophobic compounds. In this method, the extract was gradient eluted from the same afore mentioned C18 column using methanol, acetonitrile, water, 0.05% PFPA and 0.01% FA and was operated at an overall higher organic content. Another aliquot was analyzed using basic negative ion optimized conditions using a separate dedicated C18 column. The basic extracts were gradient eluted from the column using methanol and water, however with 6.5mM Ammonium Bicarbonate at pH 8. The fourth aliquot was analyzed via negative ionization following elution from a HILIC column (Waters UPLC BEH Amide 2.1x150 mm, 1.7 µm) using a gradient consisting of water and acetonitrile with 10mM Ammonium Formate, pH 10.8. The MS analysis alternated between MS and data-dependent $MS^n$ scans using dynamic exclusion. The scan range varied slighted between methods but covered 70-1000 m/z. Raw data was extracted, peak-identified and QC processed using Metabolon's hardware and software. Compounds were identified by comparison to library entries of purified standards or recurrent unknown entities.

**Statistical analyses**

**Table S1**: Two-tailed t-test for continuous variables and Fisher test for categorical variables were used to compare demographic and clinical parameters between patients enrolled at the two sites as well as between patients who developed HCC during follow-up (Cases) and those who didn't (Controls).

**Tables S2 and S3, Figures 1A and S5, Supplementary Figure and Table**: To determine the association between circulating metabolite levels and HCC outcome (at any time point; within 6 months, 12 months or 24 months prior to HCC diagnosis) or HCC treatment, linear mixed-effects modeling using Maximum

Likelihood Estimation was performed, using the "lmer" function of the "lme4" R package. Grouping based on binary clinical outcome (Cases versus Controls for HCC development vs no development; Cases-T vs Cases for HCC patients post- versus pre-treatment), time (in months to latest visit) and the time × group interaction term were modeled as fixed effects, whereas patient ID was modeled as a random effect to account for repeated measures from the same patient. Log-transformed metabolite levels were used as the outcome variables. For each metabolite, the model was represented by [$y = \beta_0 + \beta_1 x_1 + \beta_2 x_2 + \beta_3 x_1 x_2 + \mu + \epsilon$], where y is the log-transformed metabolite abundance, $\beta_0$ is the global intercept, $\beta_1$ is the coefficient for time ($x_1$), $\beta_2$ is the coefficient for group ($x_2$) (binary clinical outcome, with Controls=0 and Cases=1; or Cases=0 and Cases-T=1), $\beta_3$ is the coefficient for time × group interaction (i.e. difference between cases vs controls), $\mu$ is the patient-specific random effect, and $\epsilon$ *is the residual error.* The *p*-value of each fixed effect was calculated using normal approximation and was also corrected for multiple testing by the Benjamini-Hochberg method, giving *q*-values. The coefficient, *p*-value and *q*-value for the fixed effect "group" were used for subsequent volcano plots.

**Figures S2 and 1B:** To identify highly correlated clusters of HCC-associated metabolites, Spearman's correlation was performed between the 150 metabolites associated with HCC.

**Figure 1C:** Logistic regression was performed to determine the accuracy of each metabolite in predicting HCC development after adjusting for clinical covariates. Using the "glm.fit" function, we obtained odds ratios adjusted for age, gender and diabetes (AOR) and 95% confidence intervals for each unit increase in the level of metabolite (normalized, imputed, log-transformed values).

**Figures S3, S6, 2A and 3A**: To determine the predictive ability of a selected combination of HCC-related metabolites, the conditional inference random forest machine learning algorithm was implemented, using the "cforest" function of the "party" package, combined with the "caret" package. Demographic and genetic variables (age, gender, PNPLA3 rs738409 GG genotype, TM6SF2 rs58542926 CT/TT genotype), AFP and the HCC-associated metabolites were used as independent variables, while HCC diagnosis was

used as the binary outcome. Using the "train" function of the "caret" package, the optimal "mtry" value giving the maximum AUC was determined by 5-fold cross validation. Conditional importance scores (Mean Decrease in Accuracy) at the optimal "mtry" were generated to obtain final rankings of importance.

**Figures 2B-C and 3B-C:** To further select for top variables, recursive feature elimination was implemented using the "rfe" function of the "caret" package and incorporating resampling through 3-fold cross validation. The number of variables giving optimum model performance by the "accuracy" metric was determined. Receiver operating characteristic (ROC) curve analysis was performed to determined the predictive accuracy of a minimal panel, consisting of the 6 predictors with the highest importance by recursive feature elimination. Logistic regression models were fit using the "glm" function in R; the resulting fitted probabilities were used for graphing ROC curves and computing the area under the curve (AUC) using the pROC and ROCR packages.

**Table S4, Figure 4:** To determine the effect of demographic, clinical or genetic variables on HCC-associated metabolomic profiles, redundancy analysis (RDA) was performed using the "capscale" function in the Vegan package for R. Normalized, imputed, log-transformed values of the HCC-associated metabolites were used as the response variables, while age, gender, etiologies, PNPLA3 rs738409 (in increasing number of the risk allele G) and TM6SF2 rs58542926 (in increasing number of the risk allele T), were used as the explanatory variables. To identify the specific metabolites affected by gender, TM6SF2 rs58542926 and PNPLA3 rs738409, linear mixed-effects modeling was again performed. For each of the three clinical variables, the model was represented by [$y = \beta_0 + \beta_1 x_1 + \mu + \epsilon$], where y is the log-transformed metabolite abundance, $\beta_0$ is the global intercept, $\beta_1$ is the coefficient for the clinical variable ($x_1$) (gender: female=0, male=1; TM6SF2 rs58542926: CC=0, CT=1, TT=2; PNPLA3 rs738409: CC=0, GG=1), $\mu$ is the patient-specific random effect, and $\epsilon$ is the residual error. For each model, the coefficient, *p*-value and Benjamini-Hochberg-adjusted *q*-values of the clinical variable was generated.

**Table S5, Figure 5:** To determine whether HCC-associated metabolites could distinguish between patients with LI-RADS-3 lesions that developed HCC (Cases-LR3) and those that did not (Controls-LR3), linear mixed-effects modeling was again performed using only Controls and Cases with LI-RADS-3 lesions. Log-transformed metabolite levels were used as the outcome variable. For each metabolite, the model was represented by [$y = \beta_0 + \beta_1 x_1 + \mu + \epsilon$], where y is the log-transformed metabolite abundance, $\beta_0$ is the global intercept, $\beta_1$ is the coefficient for group ($x_1$) (binary clinical outcome, with Controls=0 and Cases=1), $\mu$ is the patient-specific random effect, and $\epsilon$ is the residual error. The *p*-value of each fixed effect was calculated using normal approximation and was also corrected for multiple testing by the Benjamini-Hochberg method, giving *q*-values. The coefficient, *p*-value and *q*-value for the fixed effect "group" were used for subsequent volcano plots. Using the 23 significant metabolites from linear mixed-effects modeling, principal component analysis (PCA) was performed using the "cmdscale" function, and Euclidean distances based on log-transformed metabolite levels. Beta dispersion and permutational multivariate analysis of variance (PERMANOVA) tests were performed with the Vegan package. Ellipses were drawn using the standard deviation of point scores.

**Supplementary figures**

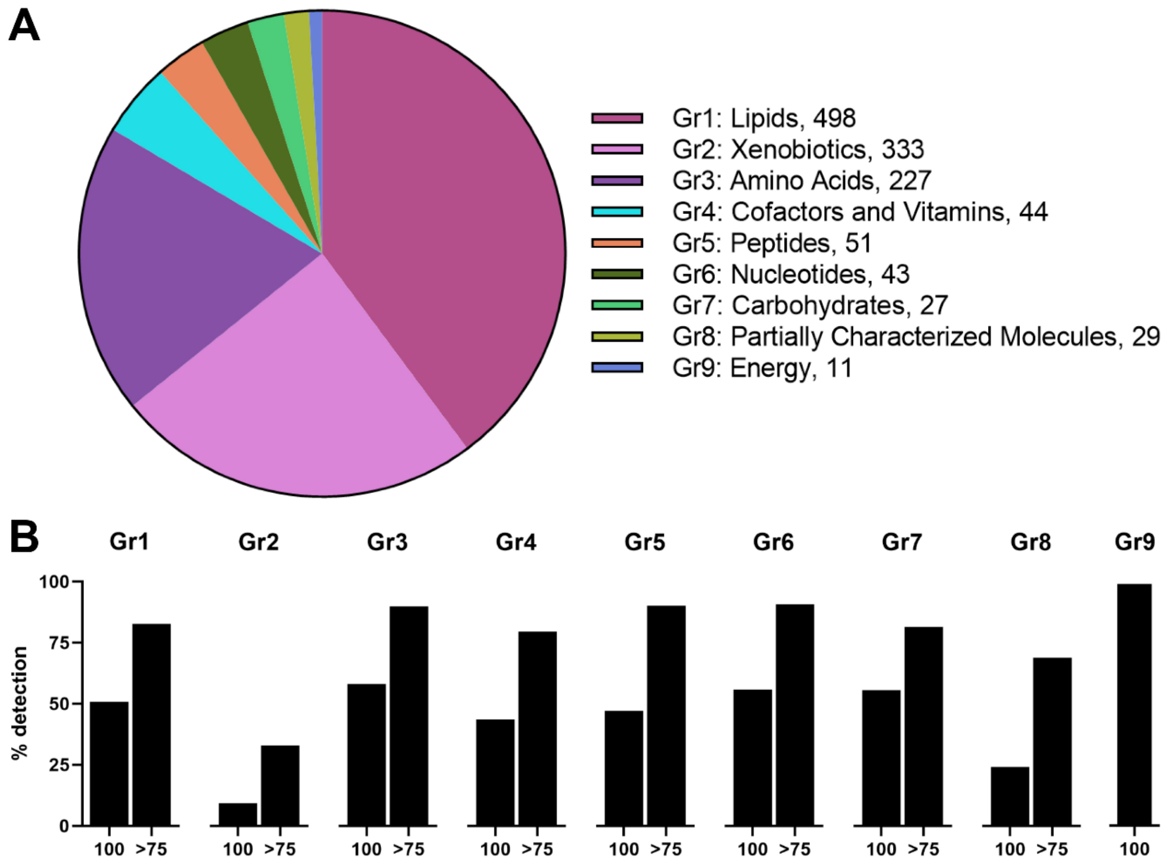


**Fig. S1. Overall distribution of the metabolites measured in the study. (A)** Pie chart displaying the distribution among different pathways, of the metabolites measured in the 612 serum samples. **(B)** Column graphs showing for each pathway (Gr1 to Gr9), the percentage of metabolites detected in 100% of the samples or in over 75% of the samples. The majority (58.1%) of the metabolites in the Amino Acids super-pathway were detected in all 612 serum samples, with 89.9% of them detected in at least 75% of the samples. Similarly, the majority of the Lipids-related metabolites (50.8%), of the Carbohydrates-related metabolites (55.5%) and of the Nucleotides-related metabolites (55.8%), were detected in all samples, with 82.7%, 81.5% and 90.7% of them detected in at least 75% of the samples, respectively. The 11 energy-related metabolites were also detected in the large majority of samples (at least 99% of the samples). More variation was observed for the detection of the Cofactors and Vitamins-related metabolites (43.5% detected in all samples, 79.5% detected in at least 75% of the samples) and of the Peptides-related

metabolites (47.1% detected in all samples, 90.2% detected in at least 75% of the samples). The largest variations were observed as anticipated for the Partially Characterized Molecules, with 24.1% of them detected in all samples and 68.9% in at least 75% of the samples, as well as for Xenobiotics with only 9.3% detected in all samples and 33.0% detected in at least 75% of the samples. A large number of xenobiotics (33.6%) were detected in less than 5% of the samples.

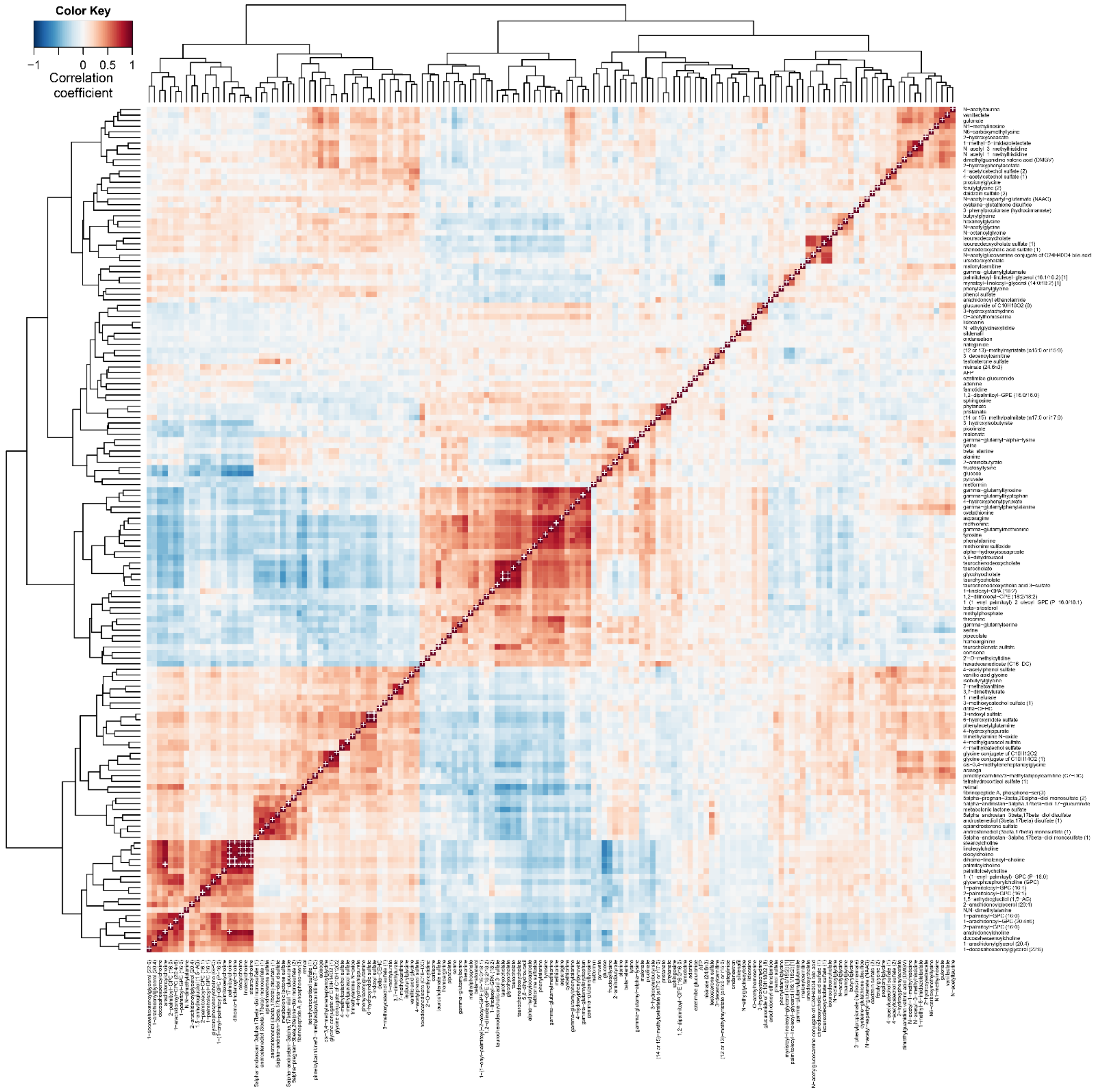


**Fig. S2. Correlation matrix of the 151 differential metabolites between cases and controls.**

Metabolites with a Spearman's correlation coefficient of $r>0.9$ and $p<0.05$ are indicated with a white cross.

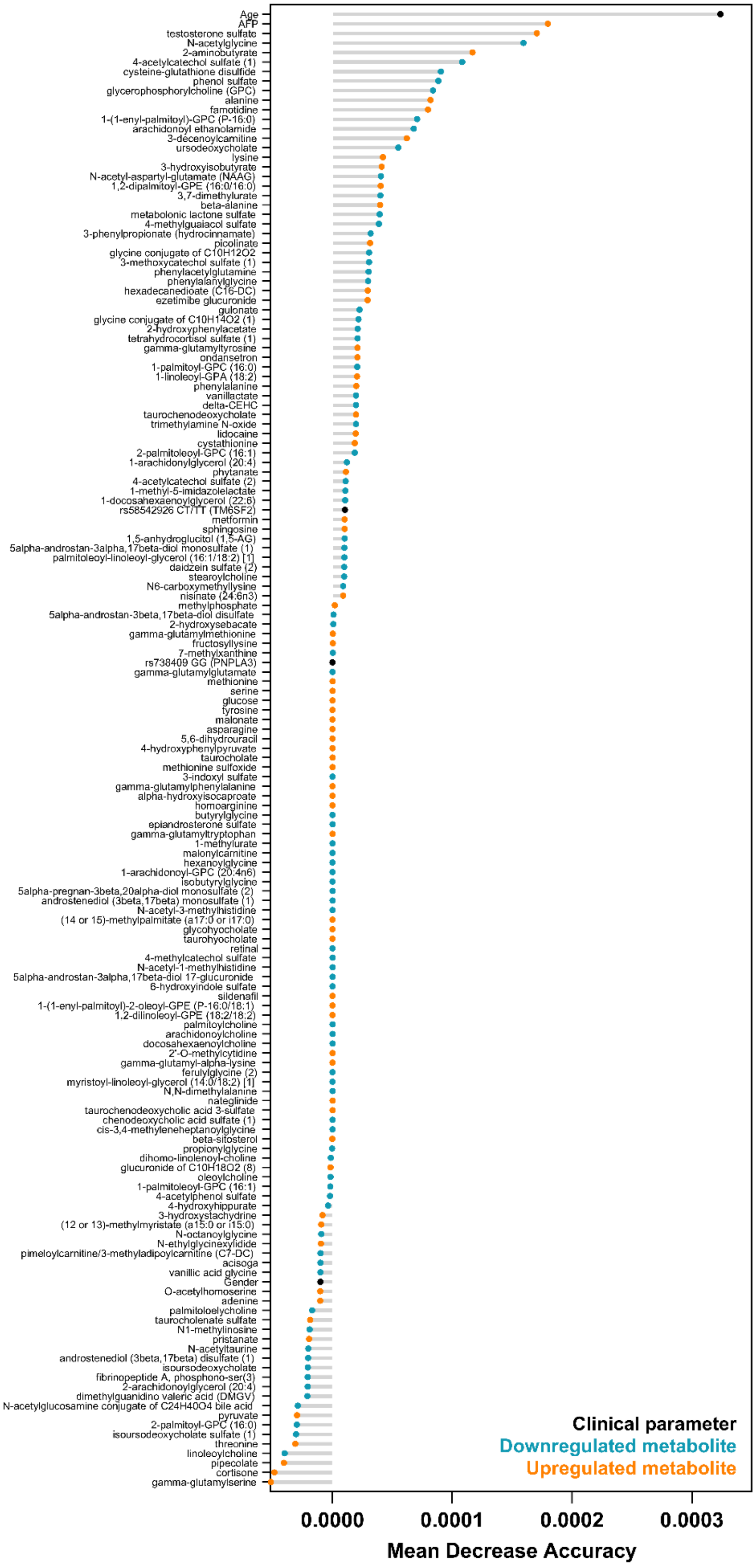

Age
AFP
testosterone sulfate
N-acetylglycine
2-aminobutyrate
4-acetylcatechol sulfate (1)
cysteine-glutathione disulfide
phenol sulfate
glycerophosphorylcholine (GPC)
alanine
famotidine
1-(1-enyl-palmitoyl)-GPC (P-16:0)
arachidonoyl ethanolamide
3-decenoylcamitine
ursodeoxycholate
lysine
3-hydroxyisobutyrate
N-acetyl-aspartyl-glutamate (NAAG)
1,2-dipalmitoyl-GPE (16:0/16:0)
3,7-dimethylurate
beta-alanine
metabolonic lactone sulfate
4-methylguaiacol sulfate
3-phenylpropionate (hydrocinnamate)
picolinate
glycine conjugate of C10H12O2
3-methoxycatechol sulfate (1)
phenylacetylglutamine
phenylalanylglycine
hexadecanedioate (C16-DC)
ezetimibe glucuronide
gulonate
glycine conjugate of C10H14O2 (1)
2-hydroxyphenylacetate
tetrahydrocortisol sulfate (1)
gamma-glutamyltyrosine
ondansetron
1-palmitoyl-GPC (16:0)
1-linoleoyl-GPA (18:2)
phenylalanine
vanillactate
delta-CEHC
taurochenodeoxycholate
trimethylamine N-oxide
lidocaine
cystathionine
2-palmitoleoyl-GPC (16:1)
1-arachidonylglycerol (20:4)
phytanate
4-acetylcatechol sulfate (2)
1-methyl-5-imidazolelactate
1-docosahexaenoylglycerol (22:6)
rs58542926 CT/TT (TM6SF2)
metformin
sphingosine
1,5-anhydroglucitol (1,5-AG)
5alpha-androstan-3alpha,17beta-diol monosulfate (1)
palmitoleoyl-linoleoyl-glycerol (16:1/18:2) [1]
daidzein sulfate (2)
stearoylcholine
N6-carboxymethyllysine
nisinate (24:6n3)
methylphosphate
5alpha-androstan-3beta,17beta-diol disulfate
2-hydroxysebacate
gamma-glutamylmethionine
fructosyllysine
7-methylxanthine
rs738409 GG (PNPLA3)
gamma-glutamylglutamate
methionine
serine
glucose
tyrosine
malonate
asparagine
5,6-dihydrouracil
4-hydroxyphenylpyruvate
taurocholate
methionine sulfoxide
3-indoxyl sulfate
gamma-glutamylphenylalanine
alpha-hydroxyisocaproate
homoarginine
butyrylglycine
epiandrosterone sulfate
gamma-glutamyltryptophan
1-methylurate
malonylcarnitine
hexanoylglycine
1-arachidonoyl-GPC (20:4n6)
isobutyrylglycine
5alpha-pregnan-3beta,20alpha-diol monosulfate (2)
androstenediol (3beta,17beta) monosulfate (1)
N-acetyl-3-methylhistidine
(14 or 15)-methylpalmitate (a17:0 or i17:0)
glycohyocholate
taurohyocholate
retinal
4-methylcatechol sulfate
N-acetyl-1-methylhistidine
5alpha-androstan-3alpha,17beta-diol 17-glucuronide
6-hydroxyindole sulfate
sildenafil
1-(1-enyl-palmitoyl)-2-oleoyl-GPE (P-16:0/18:1)
1,2-dilinoleoyl-GPE (18:2/18:2)
palmitoylcholine
arachidonoylcholine
docosahexaenoylcholine
2'-O-methylcytidine
gamma-glutamyl-alpha-lysine
ferulylglycine (2)
myristoyl-linoleoyl-glycerol (14:0/18:2) [1]
N,N-dimethylalanine
nateglinide
taurochenodeoxycholic acid 3-sulfate
chenodeoxycholic acid sulfate (1)
cis-3,4-methyleneheptanoylglycine
beta-sitosterol
propionylglycine
dihomo-linolenoyl-choline
glucuronide of C10H18O2 (8)
oleoylcholine
1-palmitoleoyl-GPC (16:1)
4-acetylphenol sulfate
4-hydroxyhippurate
3-hydroxystachydrine
(12 or 13)-methylmyristate (a15:0 or i15:0)
N-octanoylglycine
N-ethylglycinexylidide
pimeloylcarnitine/3-methyladipoylcarnitine (C7-DC)
acisoga
vanillic acid glycine
Gender
O-acetylhomoserine
adenine
palmitoloelycholine
taurocholenate sulfate
N1-methylinosine
pristanate
N-acetyltaurine
androstenediol (3beta,17beta) disulfate (1)
isoursodeoxycholate
fibrinopeptide A, phosphono-ser(3)
2-arachidonoylglycerol (20:4)
dimethylguanidino valeric acid (DMGV)
N-acetylglucosamine conjugate of C24H40O4 bile acid
pyruvate
2-palmitoyl-GPC (16:0)
isoursodeoxycholate sulfate (1)
threonine
linoleoylcholine
pipecolate
cortisone
gamma-glutamylserine
Clinical parameter
Downregulated metabolite
Upregulated metabolite
0.0000
0.0001
0.0002
0.0003
Mean Decrease Accuracy

**Fig. S3. Importance of individual clinical parameters and metabolites in predicting HCC diagnosis, as determined by conditional inference random forest.** The conditional inference random forest machine learning algorithm was implemented to determine the contribution of each clinical parameter and metabolite to the prediction of HCC diagnosis. The importance of each variable was determined by the permutation-based mean decrease accuracy, representing the loss in model performance when each variable is excluded. Variables are sorted by descending importance.

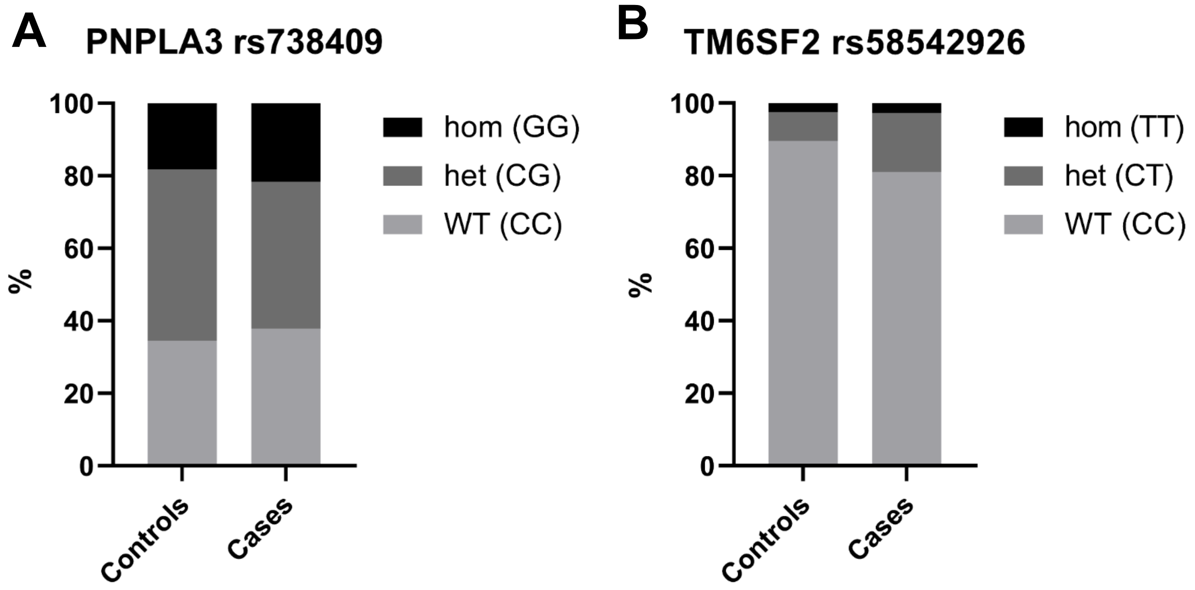


**Fig. S4. PNPLA3 rs738409 and TM6SF2 rs58542926 genotyping in study participants.** Genotype frequencies of **(A)** PNPLA3 rs738409, and **(B)** TM6SF2 rs58542926 in the 203 patients included in this study. WT: wild-type; het: heterozygous; hom: homozygous.

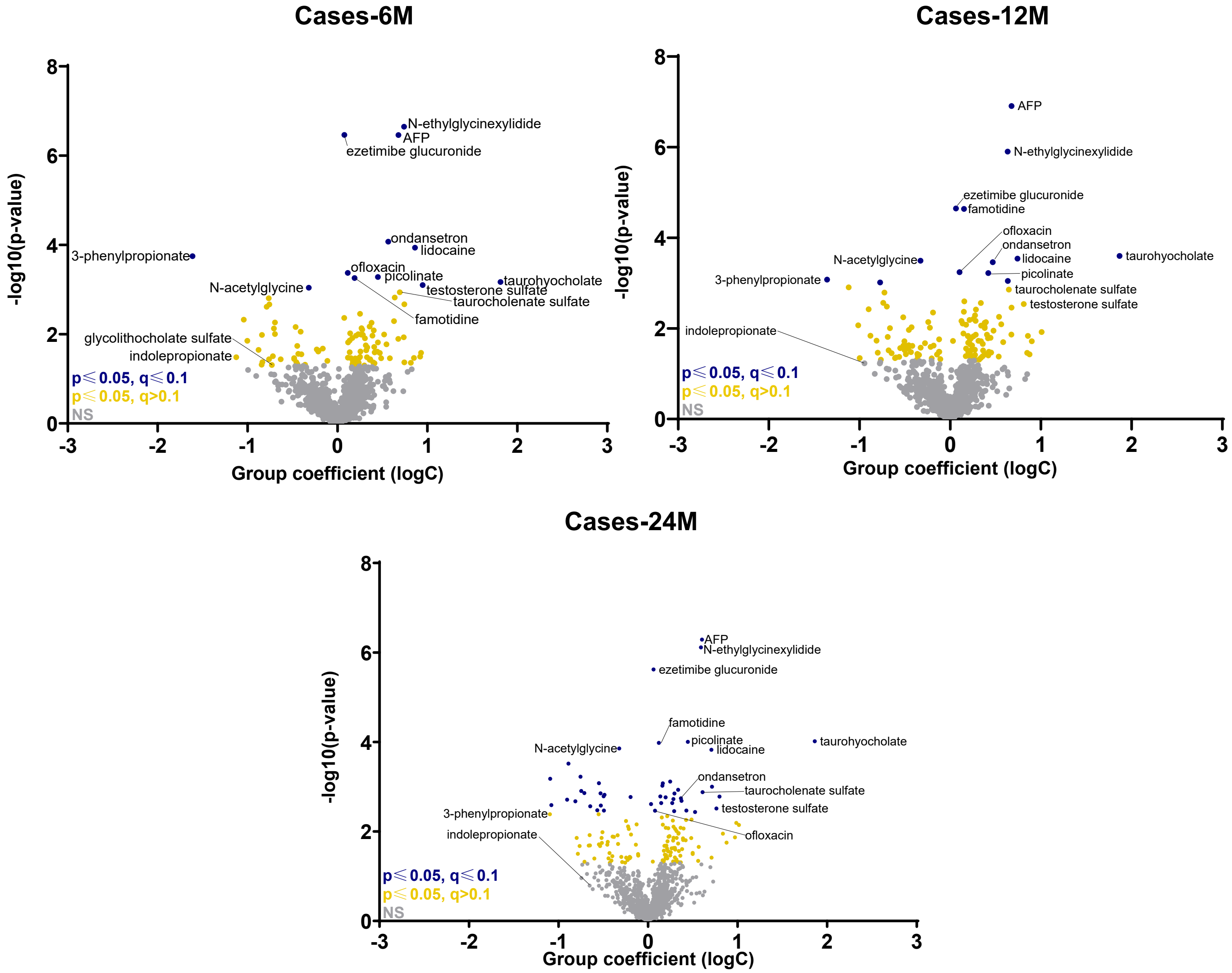


**Fig. S5. Metabolite abundance changes in Cases within 6, 12 or 24 months prior to HCC diagnosis compared to Controls.** Volcano plots for differential metabolites, between Controls and Cases-6M, Cases-12M or Cases-24M. The significance and coefficient of group (Cases within 6, 12, or 24 months versus Controls) as a fixed effect on metabolite abundance was determined by linear mixed-effects modeling. Group coefficients for log-transformed metabolite abundance (x-axis) and minus log10 p-values (y-axis) are shown. Metabolites that remained significant ($p \leq 0.05$, $q \leq 0.1$) after adjustment with the Benjamini–Hochberg method are shown in blue. Genes that did not remain significant ($p \leq 0.05$, $q > 0.1$) after adjustment with the Benjamini-Hochberg method are shown in yellow.

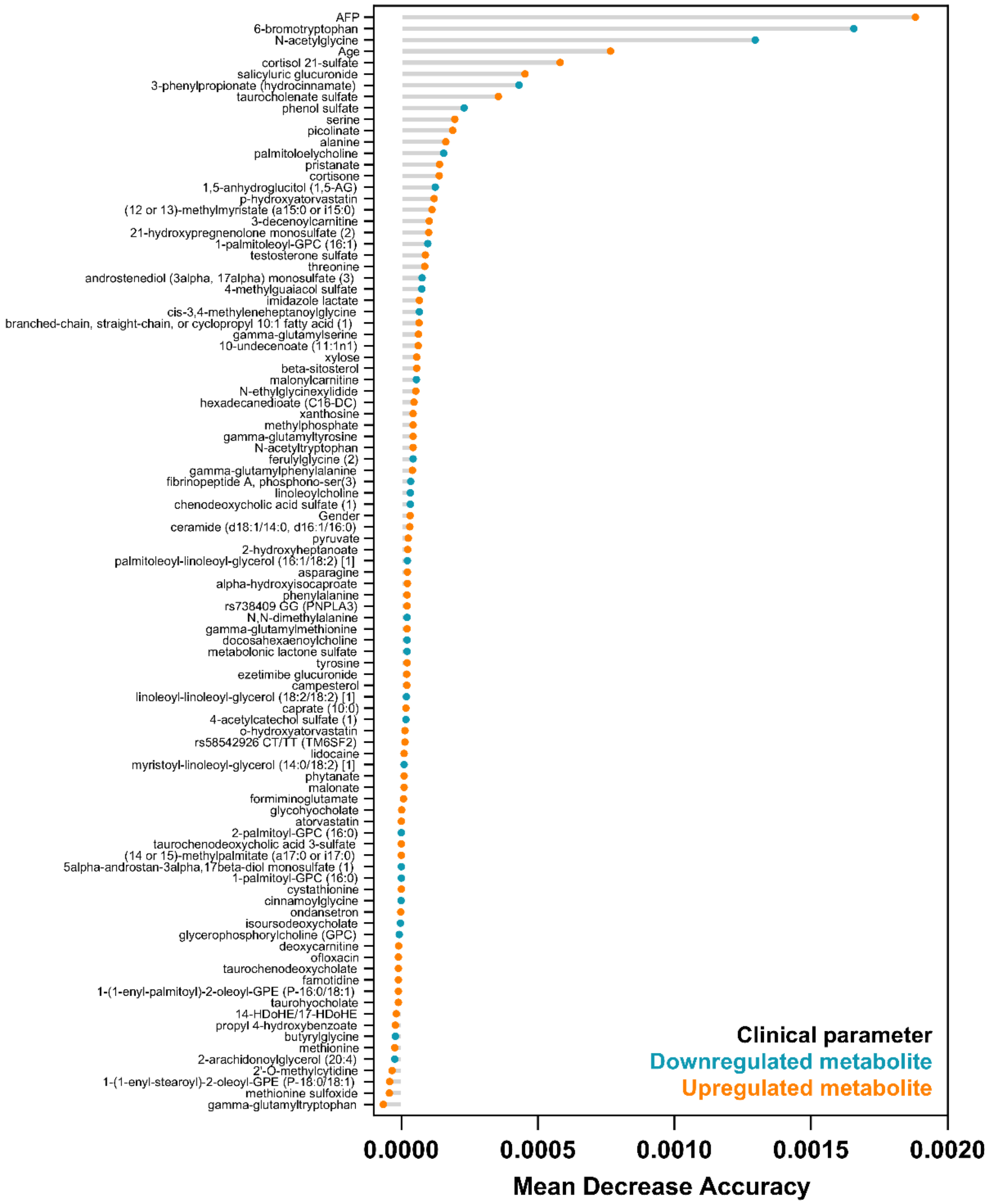


**Fig. S6. Importance of individual clinical parameters and metabolites in predicting HCC diagnosis within 12 months, as determined by conditional inference random forest.** The conditional inference random forest machine learning algorithm was implemented to determine the contribution of each clinical parameter and metabolite to the prediction of HCC diagnosis within 12 months of follow-up. The importance of each variable was determined by the permutation-based mean decrease accuracy, representing the loss in model performance when each variable is excluded. Variables are sorted by descending importance.

## Supplementary tables

**Table S1: Demographic and clinical parameters for 203 cirrhotic patients included in the study.** Data are displayed as n (%) or mean (range) - median. AFP: alpha-fetoprotein; ALP: alkaline phosphatase; ALT: alanine aminotransferase; AST: aspartate aminotransferase; MELD score: model for end stage liver disease; NASH/NAFLD: nonalcoholic steatohepatitis/nonalcoholic fatty liver disease. P-values between the two sites and between Cases and Controls are shown.

| Demographics | ALL (n=203) | Site 1 (n=108) | Site 2 (n=95) | p values | Controls (n=165) | Cases (n=38) | p values |
|---|---|---|---|---|---|---|---|
| **Gender (male)** | 102 (50%) | 64 (59%) | 38 (40%) | 0.231 | 81 (49%) | 21 (55%) | 0.590 |
| **Race** | | | | 0.071 | | | 0.801 |
| *White* | 178 (88%) | 100 (93%) | 78 (82%) | | 146 (88%) | 32 (84%) | |
| *Black* | 17 (8%) | 5 (5%) | 12 (13%) | | 13 (8%) | 4 (11%) | |
| *Other* | 8 (4%) | 3 (3%) | 5 (5%) | | 6 (4%) | 2 (5%) | |
| **Ethnicity** | | | | 0.324 | | | 0.827 |
| *Hispanic* | 41 (20%) | 19 (18%) | 22 (23%) | | 33 (20%) | 8 (21%) | |
| *Non-Hispanic* | 162 (80%) | 89 (82%) | 73 (77%) | | 132 (80%) | 30 (79%) | |
| **Etiology** | | | | 0.01 | | | 0.439 |
| *HCV* | 62 (31%) | 28 (26%) | 34 (36%) | | 49 (30%) | 13 (34%) | |
| *HBV* | 14 (7%) | 6 (6%) | 8 (8%) | | 13 (8%) | 1 (3%) | |
| *NASH/NAFLD* | 77 (38%) | 34 (31%) | 43 (45%) | | 59 (36%) | 18 (47%) | |
| *Alcohol* | 52 (26%) | 38 (35%) | 14 (15%) | | 42 (25%) | 10 (26%) | |
| *Other* | 29 (14%) | 13 (12%) | 16 (17%) | | 26 (16%) | 3 (8%) | |
| **Ascites** | | | | <0.001 | | | 0.633 |
| *Absent* | 80 (39%) | 20 (19%) | 60 (63%) | | 63 (38%) | 17 (45%) | |
| *Controlled medically* | 113 (56%) | 79 (73%) | 34 (36%) | | 93 (56%) | 20 (53%) | |
| *Poorly controlled* | 10 (5%) | 9 (8%) | 1 (1%) | | 9 (5%) | 1 (3%) | |
| **Encephalopathy** | | | | <0.001 | | | 0.173 |
| *Absent* | 95 (47%) | 28 (26%) | 67 (71%) | | 81 (49%) | 14 (37%) | |
| *Controlled medically* | 108 (53%) | 80 (74%) | 28 (29%) | | 84 (51%) | 24 (63%) | |
| **Child Pugh Class** | | | | <0.001 | | | 0.632 |
| *Child Class A* | 81 (40%) | 16 (15%) | 65 (68%) | | 68 (41%) | 13 (34%) | |
| *Child Class B* | 98 (48%) | 71 (66%) | 27 (28%) | | 77 (47%) | 21 (55%) | |
| *Child Class C* | 24 (12%) | 21 (19%) | 3 (3%) | | 20 (12%) | 4 (11%) | |
| **Child Pugh Score** | 7 (5-11) - 7 | 8 (5-11) - 8 | 6 (5-10) - 6 | <0.001 | 7.1 (5-11) -7 | 7.4 (5-11) -8 | 0.133 |
| **Diabetes** | 91 (45%) | 51 (47%) | 40 (42%) | 0.483 | 67 (41%) | 24 (63%) | 0.018 |
| **Age** | 61 (24-83) -62 | 61 (24-78) -63 | 60 (24-83) -61 | 0.753 | 60 (24-83) -61 | 64 (48-79) -66 | 0.010 |
| **Body Mass Index** | 30.8 (18.5-49.5) -29.4 | 30.8 (18.9-45.1) -29.1 | 30.9 (18.5-49.5) -30.2 | 0.835 | 31.1 (18.5-48.6) - 29.9 | 29.6 (20.6-49.5) -28.3 | 0.189 |
| **MELD Score** | 11 (6-20) -11 | 13 (7-20) -13 | 9 (6-20) -8 | <0.001 | 10.9 (6-20) -10 | 11.9 (6-20) -12 | 0.133 |
| **Clinical Labs** | | | | | | | |
| **Protein (g/dL)** | 7.3 (3.7-9.9) -7.4 | 7.2 (3.0-9.9) -7.2 | 7.3 (5.2-8.5) -7.4 | 0.547 | 7.3 (3.7-9.3) -7.4 | 7.2 (5.5-9.9) -7.3 | 0.589 |
| **ALB (g/dL)** | 3.6 (1.2-5.0) -3.6 | 3.3 (1.2-4.8) -3.3 | 3.9 (2.5-5.0) -4.0 | <0.001 | 3.6 (1.2-5) -3.6 | 3.4 (1.8-4.6) -3.4 | 0.040 |
| **Total Bilirubin (mg/dL)** | 1.5 (0.2-7.7) -1.1 | 1.8 (0.2-5.9) -1.4 | 1.3 (0.3-7.7) -1.0 | 0.004 | 1.5 (0.2-5.7) -1.1 | 1.9 (0.3-7.7) -1.5 | 0.035 |

| | | | | | | | |
|---|---|---|---|---|---|---|---|
| **Bilirubin Direct (mg/dL)** | 0.6 (0.1-4.1) -0.4 | 0.6 (0.2-2.8) -0.4 | 0.5 (0.1-4.1) -0.3 | 0.666 | 0.5 (0.1-4.1) -0.4 | 0.7 (0.1-3.7) -0.5 | 0.053 |
| **ALK (U/L)** | 125.2 (11-911) -104 | 137 (54-911) -111 | 112 (11-413) -99 | 0.046 | 122.2 (11-911) -99 | 137.9 (61-273) -133.5 | 0.321 |
| **AST (U/L)** | 43.1 (14-225) -37 | 46 (14-225) -42 | 40 (16-223) -33 | 0.095 | 41.3 (14-223) -36 | 51.1 (19-225) -46 | 0.045 |
| **ALT (U/L)** | 33.3 (8-236) -26 | 34 (8-236) -26 | 33 (10-131) -26 | 0.831 | 32.2 (8-131) -26 | 37.8 (10-236) -28 | 0.199 |
| **AFP (ng/mL)** | 53.2 (0.9-5848) -3.8 | 6.7 (0.9-99.7) -3.7 | 106.4 (1.4-5848) - 4.1 | 0.118 | 4.4 (0.9-43) -3.7 | 271.7 (1.4-5848) -4.2 | 0.001 |
| **Platelets (billion/L)** | 118 (5.5-334) -104 | 114 (5.5-334) -97.5 | 123 (33-330) -113 | 0.272 | 119.7 (5.5-334) -107.5 | 108.9 (44-245) -98 | 0.323 |

**Table S2: Abundance changes in metabolites between controls and cases.** Significance of metabolites abundance between cases and controls was determined by linear mixed model. Super-pathway groups for metabolites are abbreviated as follows: Gr1: Lipids, Gr2: Xenobiotics, Gr3: Amino Acids: Gr4: Cofactors and Vitamins, Gr5: Peptides, Gr6: Nucleotides, Gr7: Carbohydrates, Gr8: Partially Characterized Molecules. Controls (%) and Cases (%) reflect the percentage of detection of each metabolite in controls and cases samples, respectively. AFP: Alpha-fetoprotein, GPE: Glycerophosphorylethanolamine, GPC: Glycerophosphorylcholine, GPA: Glycerophosphatidic acid, CEHC: carboxyethyl-hydroxychroman, C: group coefficient (log-transformed). The metabolites are ordered from highest to lowest group coefficient (C).

| Metabolite | Group | Pathway | Controls (%) | Cases (%) | p | q | C |
|---|---|---|---|---|---|---|---|
| Taurohyocholate | Gr1 | Secondary Bile Acid Metabolism | 74.9 | 87.5 | <0.001 | 0.041 | 1.53 |
| Taurocholate | Gr1 | Primary Bile Acid Metabolism | 99.6 | 100 | 0.005 | 0.152 | 0.92 |
| Metformin | Gr2 | Drug - Metabolic | 12.4 | 26.1 | 0.049 | 0.406 | 0.89 |
| Taurochenodeoxycholate | Gr1 | Primary Bile Acid Metabolism | 100 | 100 | 0.01 | 0.242 | 0.82 |
| 3-hydroxystachydrine | Gr2 | Food Component/Plant | 68.6 | 80.7 | 0.05 | 0.408 | 0.80 |
| Taurochenodeoxycholic acid 3-sulfate | Gr1 | Secondary Bile Acid Metabolism | 98.0 | 95.5 | 0.012 | 0.242 | 0.78 |
| Glycohyocholate | Gr1 | Secondary Bile Acid Metabolism | 98.2 | 98.9 | 0.013 | 0.247 | 0.70 |
| Pristanate | Gr1 | Fatty Acid, Branched | 56.1 | 68.2 | 0.002 | 0.107 | 0.67 |
| Beta-sitosterol | Gr1 | Sterol | 65.9 | 71.6 | 0.005 | 0.149 | 0.52 |
| Testosterone sulfate | Gr1 | Androgenic Steroids | 30.2 | 53.4 | 0.019 | 0.285 | 0.52 |
| Glucuronide of C10H18O2 (8) | Gr8 | Partially Characterized Molecules | 70.4 | 86.4 | 0.04 | 0.379 | 0.48 |
| AFP | | | 100 | 100 | <0.001 | 0.004 | 0.48 |
| Taurocholenate sulfate | Gr1 | Secondary Bile Acid Metabolism | 100 | 100 | 0.005 | 0.152 | 0.45 |
| Lidocaine | Gr2 | Drug - Analgesics, Anesthetics | 7.1 | 14.8 | 0.005 | 0.148 | 0.45 |
| (12 or 13)-methylmyristate (a15:0 or i15:0) | Gr1 | Fatty Acid, Branched | 43.5 | 51.1 | 0.008 | 0.211 | 0.45 |
| 3-decenoylcarnitine | Gr1 | Fatty Acid Metabolism (Acyl Carnitine, Monounsaturated) | 52.0 | 62.5 | 0.006 | 0.152 | 0.43 |
| O-acetylhomoserine | Gr3 | Glycine, Serine and Threonine Metabolism | 77.6 | 73.9 | 0.016 | 0.259 | 0.41 |
| 5,6-dihydrouracil | Gr6 | Pyrimidine Metabolism, Uracil containing | 91.0 | 96.6 | 0.018 | 0.28 | 0.40 |
| 1,2-dilinoleoyl-GPE (18:2/18:2) | Gr1 | Phosphatidylethanolamine (PE) | 87.6 | 89.8 | 0.033 | 0.354 | 0.36 |
| 1,2-dipalmitoyl-GPE (16:0/16:0) | Gr1 | Phosphatidylethanolamine (PE) | 58.0 | 64.8 | <0.001 | 0.041 | 0.36 |
| N-ethylglycinexylidide | Gr2 | Drug - Analgesics, Anesthetics | 5.1 | 12.5 | 0.001 | 0.068 | 0.35 |
| Picolinate | Gr3 | Tryptophan Metabolism | 99.8 | 100 | <0.001 | 0.048 | 0.35 |

| | | | | | | | |
|---|---|---|---|---|---|---|---|
| Hexadecanedioate (C16-DC) | Gr1 | Fatty Acid, Dicarboxylate | 99.2 | 98.9 | 0.015 | 0.253 | 0.32 |
| Phytanate | Gr2 | Food Component/Plant | 99.6 | 100 | 0.018 | 0.28 | 0.31 |
| Cystathionine | Gr3 | Methionine, Cysteine, SAM and Taurine Metabolism | 99.4 | 100 | 0.048 | 0.406 | 0.29 |
| Nisinate (24:6n3) | Gr1 | Long Chain Polyunsaturated Fatty Acid (n3 and n6) | 28.6 | 36.4 | 0.038 | 0.371 | 0.29 |
| Malonate | Gr1 | Fatty Acid Synthesis | 98.8 | 98.9 | 0.014 | 0.253 | 0.28 |
| Gamma-glutamylmethionine | Gr5 | Gamma-glutamyl Amino Acid | 100 | 100 | 0.001 | 0.079 | 0.28 |
| Gamma-glutamyltyrosine | Gr5 | Gamma-glutamyl Amino Acid | 100 | 100 | 0.001 | 0.068 | 0.26 |
| Pipecolate | Gr3 | Lysine Metabolism | 100 | 100 | 0.033 | 0.354 | 0.26 |
| Cortisone | Gr1 | Corticosteroids | 98.8 | 100 | 0.013 | 0.247 | 0.26 |
| Gamma-glutamyltryptophan | Gr5 | Gamma-glutamyl Amino Acid | 99.0 | 100 | 0.001 | 0.063 | 0.25 |
| Alpha-hydroxyisocaproate | Gr3 | Leucine, Isoleucine and Valine Metabolism | 98.6 | 100 | 0.022 | 0.293 | 0.24 |
| Ondansetron | Gr2 | Drug - Gastrointestinal | 3.1 | 8.0 | 0.02 | 0.287 | 0.24 |
| Methionine sulfoxide | Gr3 | Methionine, Cysteine, SAM and Taurine Metabolism | 100 | 100 | 0.003 | 0.11 | 0.24 |
| Methionine | Gr3 | Methionine, Cysteine, SAM and Taurine Metabolism | 100 | 100 | <0.001 | 0.041 | 0.23 |
| (14 or 15)-methylpalmitate (a17:0 or i17:0) | Gr1 | Fatty Acid, Branched | 98.4 | 98.9 | 0.042 | 0.386 | 0.23 |
| Pyruvate | Gr7 | Glycolysis, Gluconeogenesis, and Pyruvate Metabolism | 100 | 100 | 0.012 | 0.242 | 0.22 |
| 3-hydroxyisobutyrate | Gr3 | Leucine, Isoleucine and Valine Metabolism | 98.4 | 100 | 0.025 | 0.31 | 0.22 |
| 1-linoleoyl-GPA (18:2) | Gr1 | Lysophospholipid | 99.8 | 100 | 0.016 | 0.262 | 0.21 |
| Homoarginine | Gr3 | Urea cycle; Arginine and Proline Metabolism | 100 | 100 | 0.019 | 0.286 | 0.20 |
| Sphingosine | Gr1 | Sphingosines | 94.3 | 98.9 | 0.012 | 0.242 | 0.20 |
| 4-hydroxyphenylpyruvate | Gr3 | Tyrosine Metabolism | 100 | 100 | 0.031 | 0.352 | 0.20 |
| Tyrosine | Gr3 | Tyrosine Metabolism | 100 | 100 | 0.003 | 0.11 | 0.20 |
| Methylphosphate | Gr6 | Purine and Pyrimidine Metabolism | 100 | 100 | 0.015 | 0.253 | 0.18 |
| Fructosyllysine | Gr3 | Lysine Metabolism | 100 | 100 | 0.034 | 0.355 | 0.18 |
| Beta-alanine | Gr6 | Pyrimidine Metabolism, Uracil containing | 99.8 | 100 | 0.012 | 0.242 | 0.17 |
| Gamma-glutamylserine | Gr5 | Gamma-glutamyl Amino Acid | 99.6 | 100 | 0.002 | 0.107 | 0.16 |
| 2-aminobutyrate | Gr3 | Glutathione Metabolism | 100 | 100 | 0.015 | 0.253 | 0.16 |
| Alanine | Gr3 | Alanine and Aspartate Metabolism | 100 | 100 | <0.001 | 0.025 | 0.16 |
| 2'-O-methylcytidine | Gr6 | Pyrimidine Metabolism, Cytidine containing | 97.6 | 100 | 0.033 | 0.354 | 0.16 |
| Gamma-glutamylphenylalanine | Gr5 | Gamma-glutamyl Amino Acid | 100 | 100 | 0.033 | 0.354 | 0.15 |
| Serine | Gr3 | Glycine, Serine and Threonine Metabolism | 100 | 100 | <0.001 | 0.051 | 0.14 |
| Threonine | Gr3 | Glycine, Serine and Threonine Metabolism | 100 | 100 | 0.002 | 0.11 | 0.14 |
| Adenine | Gr6 | Purine Metabolism, Adenine containing | 100 | 100 | 0.004 | 0.138 | 0.14 |
| 1-(1-enyl-palmitoyl)-2-oleoyl-GPE (P-16:0/18:1) | Gr1 | Plasmalogen | 99.6 | 98.9 | 0.047 | 0.401 | 0.13 |
| Asparagine | Gr3 | Alanine and Aspartate Metabolism | 100 | 100 | 0.002 | 0.107 | 0.13 |

| | | | | | | | |
|---|---|---|---|---|---|---|---|
| Glucose | Gr7 | Glycolysis, Gluconeogenesis, and Pyruvate Metabolism | 100 | 100 | 0.021 | 0.291 | 0.12 |
| Phenylalanine | Gr3 | Phenylalanine Metabolism | 100 | 100 | 0.002 | 0.107 | 0.12 |
| Gamma-glutamyl-alpha-lysine | Gr5 | Gamma-glutamyl Amino Acid | 100 | 100 | 0.029 | 0.335 | 0.11 |
| Lysine | Gr3 | Lysine Metabolism | 100 | 100 | 0.025 | 0.31 | 0.07 |
| Famotidine | Gr2 | Drug - Gastrointestinal | 1.2 | 5.7 | 0.011 | 0.242 | 0.07 |
| Ezetimibe glucuronide | Gr2 | Drug - Metabolic | 0.0 | 9.1 | <.001 | 0.009 | 0.05 |
| Sildenafil | Gr2 | Drug - Cardiovascular | 0.2 | 2.3 | 0.043 | 0.389 | 0.04 |
| Nateglinide | Gr2 | Drug - Metabolic | 0.2 | 1.1 | 0.008 | 0.211 | 0.02 |
| 1-palmitoyl-GPC (16:0) | Gr1 | Lysophospholipid | 100 | 100 | 0.034 | 0.354 | -0.09 |
| GPC | Gr1 | Phospholipid Metabolism | 100 | 100 | 0.015 | 0.253 | -0.13 |
| 2-palmitoyl-GPC (16:0) | Gr1 | Lysophospholipid | 100 | 100 | 0.036 | 0.361 | -0.15 |
| 1-(1-enyl-palmitoyl)-GPC (P-16:0) | Gr1 | Lysoplasmalogen | 100 | 100 | 0.021 | 0.291 | -0.15 |
| 1-arachidonoyl-GPC (20:4n6) | Gr1 | Lysophospholipid | 100 | 100 | 0.049 | 0.406 | -0.17 |
| 1-palmitoleoyl-GPC (16:1) | Gr1 | Lysophospholipid | 100 | 100 | 0.012 | 0.242 | -0.18 |
| N1-methylinosine | Gr6 | Purine Metabolism, (Hypo)Xanthine/Inosine containing | 99.6 | 96.6 | 0.034 | 0.355 | -0.19 |
| N-acetyl-aspartyl-glutamate | Gr3 | Glutamate Metabolism | 93.9 | 89.8 | 0.025 | 0.31 | -0.19 |
| Phenylalanylglycine | Gr5 | Dipeptide | 51.0 | 25.0 | 0.011 | 0.242 | -0.20 |
| N-acetyltaurine | Gr3 | Methionine, Cysteine, SAM and Taurine Metabolism | 99.0 | 98.9 | 0.044 | 0.392 | -0.21 |
| Gulonate | Gr4 | Ascorbate and Aldarate Metabolism | 96.7 | 94.3 | 0.049 | 0.408 | -0.23 |
| Vanillactate | Gr3 | Tyrosine Metabolism | 99.0 | 96.6 | 0.022 | 0.293 | -0.24 |
| Arachidonoyl ethanolamide | Gr1 | Endocannabinoid | 50.4 | 30.7 | 0.002 | 0.107 | -0.24 |
| 2-hydroxysebacate | Gr1 | Fatty Acid, Dicarboxylate | 97.6 | 97.7 | 0.043 | 0.389 | -0.24 |
| Glycine conjugate of C10H14O2 (1) | Gr8 | Partially Characterized Molecules | 99.8 | 100 | 0.033 | 0.354 | -0.25 |
| 2-palmitoleoyl-GPC (16:1) | Gr1 | Lysophospholipid | 99.4 | 98.9 | 0.01 | 0.235 | -0.25 |
| Isobutyrylglycine | Gr3 | Leucine, Isoleucine and Valine Metabolism | 60.6 | 48.9 | 0.036 | 0.364 | -0.28 |
| Gamma-glutamylglutamate | Gr5 | Gamma-glutamyl Amino Acid | 86.1 | 83.0 | 0.023 | 0.298 | -0.29 |
| 1-arachidonylglycerol (20:4) | Gr1 | Monoacylglycerol | 99.6 | 98.9 | 0.018 | 0.28 | -0.29 |
| Retinal | Gr4 | Vitamin A Metabolism | 88.2 | 90.9 | 0.045 | 0.393 | -0.29 |
| Myristoyl-linoleoyl-glycerol (14:0/18:2) [1] | Gr1 | Diacylglycerol | 23.1 | 18.2 | 0.027 | 0.326 | -0.29 |
| N-acetylglycine | Gr3 | Glycine, Serine and Threonine Metabolism | 100 | 100 | <0.001 | 0.022 | -0.29 |
| N-octanoylglycine | Gr1 | Fatty Acid Metabolism (Acyl Glycine) | 56.1 | 28.4 | 0.011 | 0.242 | -0.30 |
| Butyrylglycine | Gr1 | Fatty Acid Metabolism (also BCAA Metabolism) | 45.1 | 31.8 | 0.028 | 0.331 | -0.30 |
| Delta-CEHC | Gr4 | Tocopherol Metabolism | 92.7 | 84.1 | 0.026 | 0.312 | -0.30 |
| 2-hydroxyphenylacetate | Gr3 | Phenylalanine Metabolism | 90.0 | 83.0 | 0.038 | 0.371 | -0.30 |
| Phenylacetylglutamine | Gr5 | Acetylated Peptides | 100 | 100 | 0.042 | 0.388 | -0.31 |
| Malonylcarnitine | Gr1 | Fatty Acid Synthesis | 68.6 | 60.2 | 0.007 | 0.192 | -0.31 |
| 4-hydroxyhippurate | Gr2 | Benzoate Metabolism | 100 | 100 | 0.033 | 0.354 | -0.32 |

| | | | | | | | |
|---|---|---|---|---|---|---|---|
| N-acetyl-1-methylhistidine | Gr3 | Histidine Metabolism | 98.6 | 96.6 | 0.05 | 0.408 | -0.34 |
| Trimethylamine N-oxide | Gr1 | Phospholipid Metabolism | 100 | 100 | 0.024 | 0.31 | -0.35 |
| Hexanoylglycine | Gr1 | Fatty Acid Metabolism (Acyl Glycine) | 45.7 | 21.6 | 0.005 | 0.148 | -0.35 |
| Acisoga | Gr3 | Polyamine Metabolism | 94.3 | 88.6 | 0.008 | 0.211 | -0.35 |
| 5alpha-androstan-3alpha,17beta-diol 17-glucuronide | Gr1 | Androgenic Steroids | 31.0 | 22.7 | 0.035 | 0.355 | -0.36 |
| N,N-dimethylalanine | Gr3 | Alanine and Aspartate Metabolism | 95.7 | 94.3 | 0.035 | 0.355 | -0.36 |
| Tetrahydrocortisol sulfate (1) | Gr1 | Corticosteroids | 66.7 | 56.8 | 0.045 | 0.393 | -0.37 |
| Propionylglycine | Gr1 | Fatty Acid Metabolism (also BCAA Metabolism) | 55.3 | 52.3 | 0.015 | 0.253 | -0.37 |
| Pimeloylcarnitine/3-methyladipoylcarnitine (C7-DC) | Gr1 | Fatty Acid Metabolism (Acyl Carnitine, Dicarboxylate) | 90.0 | 88.6 | 0.02 | 0.286 | -0.37 |
| Palmitoylcholine | Gr1 | Fatty Acid Metabolism (Acyl Choline) | 99.8 | 100 | 0.003 | 0.131 | -0.38 |
| 3-indoxyl sulfate | Gr3 | Tryptophan Metabolism | 100 | 100 | 0.022 | 0.293 | -0.38 |
| Oleoylcholine | Gr1 | Fatty Acid Metabolism (Acyl Choline) | 99.4 | 95.5 | 0.003 | 0.131 | -0.39 |
| 1-docosahexaenoylglycerol (22:6) | Gr1 | Monoacylglycerol | 63.3 | 48.9 | 0.041 | 0.384 | -0.39 |
| Linoleoylcholine | Gr1 | Fatty Acid Metabolism (Acyl Choline) | 100 | 97.7 | 0.005 | 0.148 | -0.39 |
| Ferulylglycine (2) | Gr2 | Food Component/Plant | 34.9 | 21.6 | 0.021 | 0.293 | -0.39 |
| Phenol sulfate | Gr3 | Tyrosine Metabolism | 100 | 100 | 0.045 | 0.393 | -0.40 |
| Stearoylcholine | Gr1 | Fatty Acid Metabolism (Acyl Choline) | 99.2 | 93.2 | 0.004 | 0.142 | -0.40 |
| 1-methyl-5-imidazolelactate | Gr3 | Histidine Metabolism | 98.2 | 92.0 | 0.021 | 0.293 | -0.42 |
| 5alpha-pregnan-3beta,20alpha-diol monosulfate (2) | Gr1 | Progestin Steroids | 79.2 | 70.5 | 0.027 | 0.326 | -0.42 |
| 3,7-dimethylurate | Gr2 | Xanthine Metabolism | 73.3 | 54.5 | 0.033 | 0.354 | -0.42 |
| 4-acetylcatechol sulfate (2) | Gr2 | Food Component/Plant | 44.7 | 27.3 | 0.031 | 0.352 | -0.44 |
| 4-acetylphenol sulfate | Gr2 | Benzoate Metabolism | 90.4 | 85.2 | 0.014 | 0.253 | -0.45 |
| 3-methoxycatechol sulfate (1) | Gr2 | Benzoate Metabolism | 99.2 | 98.9 | 0.046 | 0.398 | -0.45 |
| Palmitoloelycholine | Gr1 | Fatty Acid Metabolism (Acyl Choline) | 80.0 | 54.5 | 0.002 | 0.108 | -0.46 |
| glycine conjugate of C10H12O2 | Gr8 | Partially Characterized Molecules | 80.8 | 70.5 | 0.004 | 0.138 | -0.46 |
| Epiandrosterone sulfate | Gr1 | Androgenic Steroids | 99.8 | 100 | 0.038 | 0.371 | -0.47 |
| 1-methylurate | Gr2 | Xanthine Metabolism | 77.1 | 72.7 | 0.011 | 0.242 | -0.47 |
| Dihomo-linolenoyl-choline | Gr1 | Fatty Acid Metabolism (Acyl Choline) | 95.1 | 86.4 | 0.004 | 0.138 | -0.47 |
| N6-carboxymethyllysine | Gr7 | Advanced Glycation End-product | 75.7 | 60.2 | 0.045 | 0.393 | -0.47 |
| Arachidonoylcholine | Gr1 | Fatty Acid Metabolism (Acyl Choline) | 99.0 | 94.3 | 0.004 | 0.138 | -0.47 |
| cis-3,4-methyleneheptanoylglycine | Gr1 | Fatty Acid Metabolism (Acyl Glycine) | 94.7 | 93.2 | 0.001 | 0.068 | -0.48 |
| 6-hydroxyindole sulfate | Gr2 | Chemical | 98.8 | 96.6 | 0.012 | 0.242 | -0.48 |

| | | | | | | | |
|---|---|---|---|---|---|---|---|
| Daidzein sulfate (2) | Gr2 | Food Component/Plant | 34.7 | 25.0 | 0.025 | 0.31 | -0.49 |
| Cysteine-glutathione disulfide | Gr3 | Glutathione Metabolism | 88.8 | 84.1 | 0.043 | 0.389 | -0.50 |
| Docosahexaenoylcholine | Gr1 | Fatty Acid Metabolism (Acyl Choline) | 84.3 | 68.2 | 0.012 | 0.242 | -0.50 |
| 7-methylxanthine | Gr2 | Xanthine Metabolism | 88.0 | 86.4 | 0.023 | 0.298 | -0.50 |
| Androstenediol (3beta,17beta) monosulfate (1) | Gr1 | Androgenic Steroids | 90.6 | 87.5 | 0.039 | 0.371 | -0.51 |
| Dimethylguanidino valeric acid | Gr3 | Urea cycle; Arginine and Proline Metabolism | 79.4 | 69.3 | 0.050 | 0.408 | -0.51 |
| Palmitoleoyl-linoleoyl-glycerol (16:1/18:2) [1] | Gr1 | Diacylglycerol | 52.7 | 39.8 | 0.005 | 0.149 | -0.52 |
| Androstenediol (3beta,17beta) disulfate (1) | Gr1 | Androgenic Steroids | 99.6 | 100 | 0.044 | 0.39 | -0.52 |
| N-acetyl-3-methylhistidine | Gr3 | Histidine Metabolism | 73.9 | 67.0 | 0.009 | 0.232 | -0.55 |
| Metabolonic lactone sulfate | Gr8 | Partially Characterized Molecules | 54.7 | 53.4 | 0.031 | 0.352 | -0.55 |
| 5alpha-androstan-3alpha,17beta-diol monosulfate (1) | Gr1 | Androgenic Steroids | 28.4 | 15.9 | 0.003 | 0.131 | -0.56 |
| N-acetylglucosamine conjugate of C24H40O4 bile acid | Gr8 | Partially Characterized Molecules | 65.9 | 48.9 | 0.019 | 0.285 | -0.57 |
| Vanillic acid glycine | Gr2 | Food Component/Plant | 75.7 | 53.4 | 0.014 | 0.253 | -0.60 |
| 4-methylcatechol sulfate | Gr2 | Benzoate Metabolism | 98.8 | 98.9 | 0.040 | 0.379 | -0.61 |
| Isoursodeoxycholate sulfate (1) | Gr1 | Secondary Bile Acid Metabolism | 65.3 | 53.4 | 0.015 | 0.253 | -0.62 |
| 1,5-anhydroglucitol | Gr7 | Glycolysis, Gluconeogenesis, and Pyruvate Metabolism | 99.8 | 100 | 0.002 | 0.107 | -0.66 |
| 3-phenylpropionate (hydrocinnamate) | Gr2 | Benzoate Metabolism | 80.2 | 63.6 | 0.040 | 0.378 | -0.68 |
| Fibrinopeptide A, phosphono-ser(3) | Gr5 | Fibrinogen Cleavage Peptide | 87.1 | 80.7 | <0.001 | 0.041 | -0.68 |
| Chenodeoxycholic acid sulfate (1) | Gr1 | Primary Bile Acid Metabolism | 69.0 | 51.1 | 0.002 | 0.107 | -0.69 |
| 4-methylguaiacol sulfate | Gr2 | Benzoate Metabolism | 73.3 | 61.4 | 0.012 | 0.242 | -0.70 |
| 4-acetylcatechol sulfate (1) | Gr2 | Food Component/Plant | 60.2 | 45.5 | 0.002 | 0.107 | -0.73 |
| 5alpha-androstan-3beta,17beta-diol disulfate | Gr1 | Androgenic Steroids | 86.9 | 84.1 | 0.029 | 0.338 | -0.75 |
| Ursodeoxycholate | Gr1 | Secondary Bile Acid Metabolism | 96.1 | 93.2 | 0.025 | 0.31 | -0.90 |
| 2-arachidonoylglycerol (20:4) | Gr1 | Monoacylglycerol | 61.2 | 38.6 | <0.001 | 0.039 | -1.03 |
| Isoursodeoxycholate | Gr1 | Secondary Bile Acid Metabolism | 94.7 | 93.2 | 0.001 | 0.054 | -1.05 |

**Table S3. Changes in metabolites abundance in cases collected 12 (Cases-12M) or 6 (Cases-6M) months prior to HCC diagnosis compared to controls.** Significance of metabolites abundance changes between controls and cases-12M or cases-6M was determined by linear mixed model. For these metabolites, significance between cases-24M and controls is also shown. Super-pathway groups are abbreviated as follows: Gr1: Lipids, Gr2: Xenobiotics, Gr3: Amino Acids: Gr5: Peptides, Gr6: Nucleotides, Gr7: Carbohydrates, Gr8: Partially Characterized Molecules. AFP: alpha-fetoprotein, C: group coefficient (log-transformed), *: not significant. The metabolites are separated into those with positive C and those with negative C.

| | | | **Cases-24M** | | | **Cases-12M** | | | **Cases-6M** | | |
|---|---|---|---|---|---|---|---|---|---|---|---|
| **Metabolite** | **Group** | **Pathway** | **p** | **q** | **C** | **p** | **q** | **C** | **p** | **q** | **C** |
| AFP | | | <0.001 | <0.001 | 0.60 | <0.001 | <0.001 | 0.68 | <0.001 | <0.001 | 0.68 |
| Asparagine | Gr3 | Alanine and Aspartate Metabolism | 0.001 | 0.074 | 0.16 | 0.004 | 0.213 | 0.15 | 0.014 | 0.381 | 0.14 |
| Alanine | Gr3 | Alanine and Aspartate Metabolism | 0.002 | 0.082 | 0.15 | 0.003 | 0.180 | 0.16 | 0.010 | 0.337 | 0.14 |
| testosterone sulfate | Gr1 | Androgenic Steroids | 0.003 | 0.095 | 0.76 | 0.003 | 0.180 | 0.81 | 0.001 | 0.090 | 0.95 |
| propyl 4-hydroxybenzoate | Gr2 | Benzoate Metabolism | * | * | * | 0.039 | 0.437 | 0.42 | 0.035 | 0.541 | 0.49 |
| deoxycarnitine | Gr1 | Carnitine Metabolism | * | * | * | 0.028 | 0.406 | 0.18 | 0.047 | 0.582 | 0.17 |
| ceramide (d18:1/14:0, d16:1/16:0) | Gr1 | Ceramides | * | * | * | 0.024 | 0.377 | 0.24 | 0.024 | 0.503 | 0.26 |
| Cortisone | Gr1 | Corticosteroids | 0.002 | 0.079 | 0.37 | 0.009 | 0.263 | 0.34 | 0.006 | 0.288 | 0.38 |
| cortisol 21-sulfate | Gr1 | Corticosteroids | 0.008 | 0.165 | 0.20 | 0.015 | 0.321 | 0.20 | 0.003 | 0.229 | 0.25 |
| 14-HDoHE/17-HDoHE | Gr1 | Docosanoid | 0.030 | 0.322 | 0.50 | 0.020 | 0.351 | 0.58 | 0.013 | 0.359 | 0.68 |
| salicyluric glucuronide | Gr2 | Drug - Analgesics, Anesthetics | * | * | * | 0.035 | 0.427 | 0.85 | 0.032 | 0.541 | 0.92 |
| Lidocaine | Gr2 | Drug - Analgesics, Anesthetics | <0.001 | 0.023 | 0.71 | <0.001 | 0.054 | 0.74 | <0.001 | 0.029 | 0.86 |
| N-ethylglycinexylidide | Gr2 | Drug - Analgesics, Anesthetics | <0.001 | <0.001 | 0.59 | <0.001 | 0.001 | 0.63 | <0.001 | <0.001 | 0.74 |
| Ofloxacin | Gr2 | Drug - Antibiotic | 0.003 | 0.097 | 0.08 | 0.001 | 0.075 | 0.10 | <0.001 | 0.075 | 0.12 |
| Ondansetron | Gr2 | Drug - Gastrointestinal | 0.002 | 0.078 | 0.37 | <0.001 | 0.054 | 0.47 | <0.001 | 0.026 | 0.57 |
| Famotidine | Gr2 | Drug - Gastrointestinal | <0.001 | 0.022 | 0.12 | <0.001 | 0.007 | 0.15 | 0.001 | 0.076 | 0.19 |
| Atorvastatin | Gr2 | Drug - Metabolic | 0.035 | 0.346 | 0.36 | 0.016 | 0.331 | 0.45 | 0.025 | 0.503 | 0.44 |
| p-hydroxyatorvastatin | Gr2 | Drug - Metabolic | 0.011 | 0.195 | 0.34 | 0.007 | 0.259 | 0.39 | 0.008 | 0.337 | 0.40 |
| o-hydroxyatorvastatin | Gr2 | Drug - Metabolic | 0.023 | 0.276 | 0.30 | 0.008 | 0.260 | 0.38 | 0.011 | 0.337 | 0.39 |
| ezetimibe glucuronide | Gr2 | Drug - Metabolic | <0.001 | 0.001 | 0.06 | <0.001 | 0.007 | 0.06 | <0.001 | <0.001 | 0.08 |
| 3-decenoylcarnitine | Gr1 | Fatty Acid Metabolism (Acyl Carnitine, Monounsaturated) | 0.004 | 0.100 | 0.53 | 0.001 | 0.092 | 0.64 | 0.002 | 0.130 | 0.64 |
| Malonate | Gr1 | Fatty Acid Synthesis | 0.011 | 0.196 | 0.34 | 0.009 | 0.266 | 0.38 | 0.033 | 0.541 | 0.33 |
| Pristanate | Gr1 | Fatty Acid, Branched | 0.002 | 0.076 | 0.80 | 0.014 | 0.321 | 0.68 | 0.012 | 0.356 | 0.74 |
| (12 or 13)-methylmyristate (a15:0 or i15:0) | Gr1 | Fatty Acid, Branched | 0.014 | 0.208 | 0.48 | 0.008 | 0.263 | 0.56 | 0.005 | 0.288 | 0.63 |

| | | | | | | | | | | |
|---|---|---|---|---|---|---|---|---|---|---|
| (14 or 15)-methylpalmitate (a17:0 or i17:0) | Gr1 | Fatty Acid, Branched | 0.014 | 0.208 | 0.320 | 0.050 | 0.475 | 0.28 | 0.050 | 0.585 | 0.30 |
| hexadecanedioate (C16-DC) | Gr1 | Fatty Acid, Dicarboxylate | 0.006 | 0.132 | 0.42 | 0.014 | 0.321 | 0.41 | 0.019 | 0.462 | 0.41 |
| 2-hydroxyheptanoate | Gr1 | Fatty Acid, Monohydroxy | 0.021 | 0.256 | 0.20 | 0.016 | 0.331 | 0.23 | 0.042 | 0.572 | 0.21 |
| Phytanate | Gr2 | Food Component/Plant | 0.009 | 0.166 | 0.40 | 0.049 | 0.474 | 0.32 | 0.036 | 0.541 | 0.37 |
| gamma-glutamyltyrosine | Gr5 | Gamma-glutamyl Amino Acid | 0.001 | 0.076 | 0.30 | 0.005 | 0.237 | 0.27 | 0.010 | 0.337 | 0.26 |
| gamma-glutamylmethionine | Gr5 | Gamma-glutamyl Amino Acid | 0.004 | 0.097 | 0.29 | 0.015 | 0.321 | 0.26 | 0.010 | 0.337 | 0.28 |
| gamma-glutamyltryptophan | Gr5 | Gamma-glutamyl Amino Acid | 0.002 | 0.082 | 0.27 | 0.006 | 0.256 | 0.26 | 0.007 | 0.332 | 0.27 |
| gamma-glutamylserine | Gr5 | Gamma-glutamyl Amino Acid | 0.002 | 0.076 | 0.20 | 0.008 | 0.263 | 0.18 | 0.034 | 0.541 | 0.15 |
| gamma-glutamylphenylalanine | Gr5 | Gamma-glutamyl Amino Acid | 0.021 | 0.256 | 0.18 | 0.041 | 0.437 | 0.17 | 0.043 | 0.572 | 0.18 |
| Serine | Gr3 | Glycine, Serine and Threonine Metabolism | 0.001 | 0.076 | 0.16 | 0.008 | 0.263 | 0.14 | 0.033 | 0.541 | 0.12 |
| Threonine | Gr3 | Glycine, Serine and Threonine Metabolism | 0.005 | 0.121 | 0.15 | 0.013 | 0.321 | 0.15 | 0.025 | 0.504 | 0.134 |
| Pyruvate | Gr7 | Glycolysis, Gluconeogenesis, and Pyruvate Metabolism | 0.001 | 0.076 | 0.34 | 0.003 | 0.180 | 0.34 | 0.012 | 0.358 | 0.30 |
| imidazole lactate | Gr3 | Histidine Metabolism | * | * | * | 0.029 | 0.417 | 0.24 | 0.034 | 0.541 | 0.24 |
| Formiminoglutamate | Gr3 | Histidine Metabolism | 0.014 | 0.208 | 0.28 | 0.029 | 0.417 | 0.27 | 0.007 | 0.330 | 0.36 |
| alpha-hydroxyisocaproate | Gr3 | Leucine, Isoleucine and Valine Metabolism | 0.010 | 0.181 | 0.32 | 0.019 | 0.342 | 0.31 | 0.023 | 0.502 | 0.32 |
| caprate (10:0) | Gr1 | Medium Chain Fatty Acid | 0.014 | 0.209 | 0.39 | 0.016 | 0.331 | 0.42 | 0.015 | 0.398 | 0.45 |
| 10-undecenoate (11:1n1) | Gr1 | Medium Chain Fatty Acid | 0.009 | 0.166 | 0.30 | 0.047 | 0.470 | 0.25 | 0.018 | 0.441 | 0.31 |
| Cystathionine | Gr3 | Methionine, Cysteine, SAM and Taurine Metabolism | 0.016 | 0.225 | 0.42 | 0.037 | 0.430 | 0.39 | 0.043 | 0.572 | 0.40 |
| Methionine | Gr3 | Methionine, Cysteine, SAM and Taurine Metabolism | 0.001 | 0.074 | 0.25 | 0.006 | 0.258 | 0.21 | 0.011 | 0.337 | 0.21 |
| methionine sulfoxide | Gr3 | Methionine, Cysteine, SAM and Taurine Metabolism | 0.013 | 0.208 | 0.23 | 0.033 | 0.424 | 0.21 | 0.046 | 0.582 | 0.21 |
| branched-chain, straight-chain, or cyclopropyl 10:1 fatty acid (1) | Gr8 | Partially Characterized Molecules | 0.049 | 0.399 | 0.37 | 0.033 | 0.424 | 0.43 | 0.026 | 0.505 | 0.47 |
| Xylose | Gr7 | Pentose Metabolism | * | * | * | 0.034 | 0.427 | 0.45 | 0.017 | 0.429 | 0.55 |
| Phenylalanine | Gr3 | Phenylalanine Metabolism | 0.002 | 0.076 | 0.14 | 0.007 | 0.258 | 0.12 | 0.015 | 0.398 | 0.12 |
| 1-(1-enyl-stearoyl)-2-oleoyl-GPE (P-18:0/18:1) | Gr1 | Plasmalogen | 0.033 | 0.340 | 0.19 | 0.033 | 0.424 | 0.20 | 0.050 | 0.585 | 0.19 |
| 1-(1-enyl-palmitoyl)-2-oleoyl-GPE (P-16:0/18:1) | Gr1 | Plasmalogen | 0.027 | 0.300 | 0.18 | 0.021 | 0.359 | 0.20 | 0.010 | 0.337 | 0.23 |
| 21-hydroxypregnenolone monosulfate (2) | Gr1 | Pregnenolone Steroids | * | * | * | 0.041 | 0.437 | 0.36 | 0.026 | 0.504 | 0.41 |
| taurochenodeoxycholate | Gr1 | Primary Bile Acid Metabolism | 0.007 | 0.153 | 1.02 | 0.012 | 0.304 | 1.01 | 0.026 | 0.505 | 0.93 |
| methylphosphate | Gr6 | Purine and Pyrimidine Metabolism | 0.008 | 0.166 | 0.23 | 0.018 | 0.342 | 0.22 | 0.048 | 0.585 | 0.19 |
| Xanthosine | Gr6 | Purine Metabolism, (Hypo)Xanthine/Inosine containing | * | * | * | 0.006 | 0.243 | 0.54 | 0.010 | 0.337 | 0.52 |
| 2'-O-methylcytidine | Gr6 | Pyrimidine Metabolism, Cytidine containing | 0.017 | 0.232 | 0.22 | 0.023 | 0.375 | 0.22 | 0.050 | 0.585 | 0.20 |
| Taurohyocholate | Gr1 | Secondary Bile Acid Metabolism | <0.001 | 0.022 | 1.86 | <0.001 | 0.054 | 1.87 | 0.001 | 0.084 | 1.81 |
| taurochenodeoxycholic acid 3-sulfate | Gr1 | Secondary Bile Acid Metabolism | 0.006 | 0.145 | 0.99 | 0.019 | 0.342 | 0.90 | 0.034 | 0.541 | 0.85 |
| Glycohyocholate | Gr1 | Secondary Bile Acid Metabolism | 0.011 | 0.196 | 0.84 | 0.015 | 0.321 | 0.86 | 0.043 | 0.572 | 0.74 |

| | | | | | | | | | | |
|---|---|---|---|---|---|---|---|---|---|---|
| taurocholenate sulfate | Gr1 | Secondary Bile Acid Metabolism | 0.001 | 0.076 | 0.61 | 0.001 | 0.115 | 0.65 | 0.001 | 0.110 | 0.69 |
| beta-sitosterol | Gr1 | Sterol | 0.001 | 0.076 | 0.71 | 0.003 | 0.196 | 0.68 | 0.002 | 0.157 | 0.74 |
| Campesterol | Gr1 | Sterol | 0.022 | 0.265 | 0.57 | 0.042 | 0.442 | 0.53 | 0.035 | 0.541 | 0.58 |
| N-acetyltryptophan | Gr3 | Tryptophan Metabolism | * | * | * | 0.040 | 0.437 | 0.22 | 0.044 | 0.572 | 0.24 |
| Picolinate | Gr3 | Tryptophan Metabolism | <0.001 | 0.022 | 0.44 | 0.001 | 0.075 | 0.42 | 0.001 | 0.076 | 0.45 |
| Tyrosine | Gr3 | Tyrosine Metabolism | 0.005 | 0.115 | 0.21 | 0.015 | 0.321 | 0.20 | 0.041 | 0.572 | 0.17 |
| | | | | | | | | | | | |
| N,N-dimethylalanine | Gr3 | Alanine and Aspartate Metabolism | 0.045 | 0.386 | -0.41 | 0.019 | 0.342 | -0.51 | 0.034 | 0.541 | -0.48 |
| 5alpha-androstan-3alpha,17beta-diol monosulfate (1) | Gr1 | Androgenic Steroids | 0.001 | 0.076 | -0.71 | 0.003 | 0.195 | -0.70 | 0.005 | 0.288 | -0.70 |
| androstenediol (3alpha, 17alpha) monosulfate (3) | Gr1 | Androgenic Steroids | 0.014 | 0.208 | -0.80 | 0.019 | 0.342 | -0.81 | 0.036 | 0.541 | -0.76 |
| 4-methylguaiacol sulfate | Gr2 | Benzoate Metabolism | 0.021 | 0.256 | -0.76 | 0.014 | 0.321 | -0.88 | 0.023 | 0.501 | -0.88 |
| 3-phenylpropionate (hydrocinnamate) | Gr2 | Benzoate Metabolism | 0.004 | 0.107 | -1.10 | 0.001 | 0.092 | -1.36 | <0.001 | 0.037 | -1.61 |
| myristoyl-linoleoyl-glycerol (14:0/18:2) [1] | Gr1 | Diacylglycerol | 0.013 | 0.208 | -0.38 | 0.009 | 0.266 | -0.43 | 0.007 | 0.330 | -0.47 |
| linoleoyl-linoleoyl-glycerol (18:2/18:2) [1] | Gr1 | Diacylglycerol | 0.020 | 0.253 | -0.46 | 0.028 | 0.410 | -0.46 | 0.043 | 0.572 | -0.46 |
| palmitoleoyl-linoleoyl-glycerol (16:1/18:2) [1] | Gr1 | Diacylglycerol | 0.003 | 0.087 | -0.65 | 0.002 | 0.126 | -0.73 | 0.002 | 0.130 | -0.76 |
| palmitoloelycholine | Gr1 | Fatty Acid Metabolism (Acyl Choline) | 0.003 | 0.087 | -0.53 | 0.006 | 0.243 | -0.52 | 0.028 | 0.521 | -0.44 |
| Linoleoylcholine | Gr1 | Fatty Acid Metabolism (Acyl Choline) | 0.001 | 0.074 | -0.55 | 0.040 | 0.437 | -0.36 | 0.048 | 0.585 | -0.36 |
| docosahexaenoylcholine | Gr1 | Fatty Acid Metabolism (Acyl Choline) | 0.001 | 0.076 | -0.75 | 0.003 | 0.180 | -0.74 | 0.002 | 0.168 | -0.79 |
| cis-3,4-methyleneheptanoylglycine | Gr1 | Fatty Acid Metabolism (Acyl Glycine) | 0.003 | 0.097 | -0.49 | 0.011 | 0.287 | -0.46 | 0.018 | 0.448 | -0.45 |
| Butyrylglycine | Gr1 | Fatty Acid Metabolism (also BCAA Metabolism) | 0.013 | 0.208 | -0.40 | 0.036 | 0.430 | -0.36 | 0.028 | 0.523 | -0.40 |
| Malonylcarnitine | Gr1 | Fatty Acid Synthesis | 0.012 | 0.205 | -0.34 | 0.026 | 0.397 | -0.33 | 0.009 | 0.337 | -0.41 |
| fibrinopeptide A, phosphono-ser(3) | Gr5 | Fibrinogen Cleavage Peptide | 0.001 | 0.074 | -0.75 | 0.001 | 0.092 | -0.77 | 0.002 | 0.157 | -0.76 |
| ferulylglycine (2) | Gr2 | Food Component/Plant | 0.010 | 0.189 | -0.51 | 0.025 | 0.377 | -0.48 | 0.036 | 0.541 | -0.48 |
| 4-acetylcatechol sulfate (1) | Gr2 | Food Component/Plant | 0.020 | 0.256 | -0.65 | 0.035 | 0.428 | -0.65 | 0.031 | 0.541 | -0.72 |
| Cinnamoylglycine | Gr2 | Food Component/Plant | * | * | * | 0.049 | 0.474 | -0.77 | 0.043 | 0.572 | -0.84 |
| N-acetylglycine | Gr3 | Glycine, Serine and Threonine Metabolism | <0.001 | 0.023 | -0.32 | <0.001 | 0.054 | -0.33 | 0.001 | 0.095 | -0.32 |
| 1,5-anhydroglucitol (1,5-AG) | Gr7 | Glycolysis, Gluconeogenesis, and Pyruvate Metabolism | <0.001 | 0.042 | -0.89 | 0.010 | 0.266 | -0.67 | 0.010 | 0.337 | -0.70 |
| 1-palmitoyl-GPC (16:0) | Gr1 | Lysophospholipid | 0.007 | 0.153 | -0.13 | 0.017 | 0.342 | -0.12 | 0.040 | 0.572 | -0.11 |
| 2-palmitoyl-GPC (16:0) | Gr1 | Lysophospholipid | 0.009 | 0.166 | -0.22 | 0.010 | 0.266 | -0.23 | 0.023 | 0.503 | -0.21 |
| 1-palmitoleoyl-GPC (16:1) | Gr1 | Lysophospholipid | 0.008 | 0.162 | -0.22 | 0.007 | 0.259 | -0.24 | 0.024 | 0.503 | -0.22 |
| 2-arachidonoylglycerol (20:4) | Gr1 | Monoacylglycerol | 0.001 | 0.074 | -1.09 | 0.001 | 0.110 | -1.12 | 0.005 | 0.281 | -1.04 |
| metabolonic lactone sulfate | Gr8 | Partially Characterized Molecules | 0.002 | 0.078 | -0.91 | 0.004 | 0.198 | -0.90 | 0.010 | 0.337 | -0.84 |
| glycerophosphorylcholine (GPC) | Gr1 | Phospholipid Metabolism | 0.002 | 0.076 | -0.20 | 0.004 | 0.213 | -0.19 | 0.021 | 0.483 | -0.16 |
| chenodeoxycholic acid sulfate (1) | Gr1 | Primary Bile Acid Metabolism | 0.002 | 0.079 | -0.81 | 0.015 | 0.325 | -0.69 | 0.037 | 0.541 | -0.63 |
| Isoursodeoxycholate | Gr1 | Secondary Bile Acid Metabolism | 0.003 | 0.086 | -1.08 | 0.009 | 0.263 | -1.02 | 0.014 | 0.381 | -1.00 |
| 6-bromotryptophan | Gr3 | Tryptophan Metabolism | 0.006 | 0.135 | -0.25 | 0.026 | 0.388 | -0.22 | 0.022 | 0.496 | -0.23 |
| phenol sulfate | Gr3 | Tyrosine Metabolism | 0.019 | 0.253 | -0.55 | 0.027 | 0.404 | -0.55 | 0.007 | 0.332 | -0.71 |

**Table S4. Metabolites significantly affected by PNPLA3 SNP, TM6SF2 SNP or Gender.** Metabolite significance was determined with a linear-mixed model. Super-pathway groups are abbreviated as follows: Gr1: Lipids, Gr2: Xenobiotics, Gr3: Amino Acids: Gr5: Peptides, Gr6: Nucleotides, Gr7: Carbohydrates, Gr8: Partially Characterized Molecules. C: coefficient for the clinical variable (log-transformed). The metabolites are sectioned into being significantly affected by PNPLA3, TM6SF2, or Gender. The metabolites are further separated into those with positive C and those with negative C.

| Metabolite Name | Group | Pathway | p | Q | C |
|---|---|---|---|---|---|
| **PNPLA3** | | | | | |
| taurohyocholate | Gr1 | Secondary Bile Acid Metabolism | 0.009 | 0.117 | 1.14 |
| taurochenodeoxycholic acid 3-sulfate | Gr1 | Secondary Bile Acid Metabolism | 0.007 | 0.117 | 0.89 |
| glycohyocholate | Gr1 | Secondary Bile Acid Metabolism | 0.005 | 0.117 | 0.83 |
| taurochenodeoxycholate | Gr1 | Primary Bile Acid Metabolism | 0.020 | 0.154 | 0.82 |
| taurocholate | Gr1 | Primary Bile Acid Metabolism | 0.034 | 0.191 | 0.76 |
| alpha-hydroxyisocaproate | Gr3 | Leucine, Isoleucine and Valine Metabolism | 0.019 | 0.154 | 0.25 |
| gamma-glutamylmethionine | Gr5 | Gamma-glutamyl Amino Acid | 0.010 | 0.117 | 0.23 |
| 2'-O-methylcytidine | Gr6 | Pyrimidine Metabolism, Cytidine containing | 0.021 | 0.154 | 0.23 |
| ondansetron | Gr2 | Drug - Gastrointestinal | 0.014 | 0.151 | 0.22 |
| methylphosphate | Gr6 | Purine and Pyrimidine Metabolism | 0.010 | 0.117 | 0.21 |
| 1-linoleoyl-GPA (18:2)* | Gr1 | Lysophospholipid | 0.039 | 0.200 | 0.19 |
| methionine | Gr3 | Methionine, Cysteine, SAM and Taurine Metabolism | 0.007 | 0.117 | 0.18 |
| Tyrosine | Gr3 | Tyrosine Metabolism | 0.030 | 0.188 | 0.15 |
| gamma-glutamylserine | Gr5 | Gamma-glutamyl Amino Acid | 0.006 | 0.117 | 0.13 |
| asparagine | Gr3 | Alanine and Aspartate Metabolism | 0.005 | 0.117 | 0.12 |
| Serine | Gr3 | Glycine, Serine and Threonine Metabolism | 0.022 | 0.156 | 0.10 |
| phenylalanine | Gr3 | Phenylalanine Metabolism | 0.023 | 0.156 | 0.09 |
| 1-arachidonoyl-GPC (20:4n6) | Gr1 | Lysophospholipid | 0.006 | 0.117 | -0.24 |
| 1-arachidonylglycerol (20:4) | Gr1 | Monoacylglycerol | 0.028 | 0.180 | -0.27 |
| dihomo-linolenoyl-choline | Gr1 | Fatty Acid Metabolism (Acyl Choline) | 0.034 | 0.191 | -0.38 |
| arachidonoylcholine | Gr1 | Fatty Acid Metabolism (Acyl Choline) | 0.015 | 0.151 | -0.42 |
| fibrinopeptide A, phosphono-ser(3) | Gr5 | Fibrinogen Cleavage Peptide | 0.017 | 0.154 | -0.48 |
| 1-docosahexaenoylglycerol (22:6) | Gr1 | Monoacylglycerol | 0.007 | 0.117 | -0.51 |
| 1-methylurate | Gr2 | Xanthine Metabolism | 0.004 | 0.117 | -0.52 |
| metabolonic lactone sulfate | Gr8 | Partially Characterized Molecules | 0.002 | 0.117 | -0.87 |
| **TM6SF2** | | | | | |
| 5,6-dihydrouracil | Gr6 | Pyrimidine Metabolism, Uracil containing | 0.043 | 0.421 | 0.29 |
| alpha-hydroxyisocaproate | Gr3 | Leucine, Isoleucine and Valine Metabolism | 0.018 | 0.421 | 0.21 |
| gamma-glutamylmethionine | Gr5 | Gamma-glutamyl Amino Acid | 0.041 | 0.421 | 0.16 |
| methionine | Gr3 | Methionine, Cysteine, SAM and Taurine Metabolism | 0.023 | 0.421 | 0.13 |
| 1-(1-enyl-palmitoyl)-2-oleoyl-GPE (P-16:0/18:1) | Gr1 | Plasmalogen | 0.034 | 0.421 | 0.12 |
| asparagine | Gr3 | Alanine and Aspartate Metabolism | 0.042 | 0.421 | 0.08 |
| nateglinide | Gr2 | Drug - Metabolic | 0.044 | 0.421 | 0.01 |
| N-acetyltaurine | Gr3 | Methionine, Cysteine, SAM and Taurine Metabolism | 0.023 | 0.421 | -0.21 |
| gamma-glutamylglutamate | Gr5 | Gamma-glutamyl Amino Acid | 0.004 | 0.303 | -0.29 |

| | | | | | |
|---|---|---|---|---|---|
| Acisoga | Gr3 | Polyamine Metabolism | 0.016 | 0.421 | -0.29 |
| 5alpha-androstan-3alpha,17beta-diol monosulfate (1) | Gr1 | Androgenic Steroids | 0.033 | 0.421 | -0.38 |
| tetrahydrocortisol sulfate (1) | Gr1 | Corticosteroids | 0.01 | 0.421 | -0.42 |
| androstenediol (3beta,17beta) disulfate (1) | Gr1 | Androgenic Steroids | 0.003 | 0.303 | -0.70 |
| 5alpha-androstan-3beta,17beta-diol disulfate | Gr1 | Androgenic Steroids | 0.024 | 0.421 | -0.71 |
| **Gender** | | | | | |
| 5alpha-androstan-3beta,17beta-diol disulfate | Gr1 | Androgenic Steroids | <0.001 | <0.001 | 2.48 |
| testosterone sulfate | Gr1 | Androgenic Steroids | <0.001 | <0.001 | 1.38 |
| 5alpha-androstan-3alpha,17beta-diol 17-glucuronide | Gr1 | Androgenic Steroids | <0.001 | <0.001 | 1.01 |
| epiandrosterone sulfate | Gr1 | Androgenic Steroids | <0.001 | <0.001 | 1.00 |
| androstenediol (3beta,17beta) disulfate (1) | Gr1 | Androgenic Steroids | <0.001 | <0.001 | 0.96 |
| androstenediol (3beta,17beta) monosulfate (1) | Gr1 | Androgenic Steroids | <0.001 | <0.001 | 0.91 |
| 5alpha-androstan-3alpha,17beta-diol monosulfate (1) | Gr1 | Androgenic Steroids | <0.001 | <0.001 | 0.60 |
| metabolonic lactone sulfate | Gr8 | Partially Characterized Molecules | 0.027 | 0.137 | 0.43 |
| 5alpha-pregnan-3beta,20alpha-diol monosulfate (2) | Gr1 | Progestin Steroids | 0.005 | 0.046 | 0.39 |
| 3-decenoylcarnitine | Gr1 | Fatty Acid Metabolism (Acyl Carnitine, Monounsaturated) | 0.002 | 0.024 | 0.36 |
| 7-methylxanthine | Gr2 | Xanthine Metabolism | 0.023 | 0.123 | 0.36 |
| tetrahydrocortisol sulfate (1) | Gr1 | Corticosteroids | 0.008 | 0.058 | 0.36 |
| 3,7-dimethylurate | Gr2 | Xanthine Metabolism | 0.035 | 0.162 | 0.30 |
| phytanate | Gr2 | Food Component/Plant | 0.007 | 0.057 | 0.26 |
| cortisone | Gr1 | Corticosteroids | 0.003 | 0.036 | 0.23 |
| alpha-hydroxyisocaproate | Gr3 | Leucine, Isoleucine and Valine Metabolism | 0.003 | 0.036 | 0.22 |
| N-acetyl-aspartyl-glutamate (NAAG) | Gr3 | Glutamate Metabolism | <0.001 | 0.005 | 0.22 |
| picolinate | Gr3 | Tryptophan Metabolism | 0.010 | 0.065 | 0.19 |
| homoarginine | Gr3 | Urea cycle; Arginine and Proline Metabolism | 0.010 | 0.065 | 0.17 |
| 2-aminobutyrate | Gr3 | Glutathione Metabolism | 0.019 | 0.107 | 0.12 |
| beta-alanine | Gr6 | Pyrimidine Metabolism, Uracil containing | 0.017 | 0.099 | 0.10 |
| Alanine | Gr3 | Alanine and Aspartate Metabolism | 0.035 | 0.162 | 0.06 |
| 1-palmitoleoyl-GPC (16:1) | Gr1 | Lysophospholipid | 0.014 | 0.085 | -0.13 |
| N-acetylglycine | Gr3 | Glycine, Serine and Threonine Metabolism | 0.005 | 0.046 | -0.15 |
| 1-linoleoyl-GPA (18:2) | Gr1 | Lysophospholipid | 0.008 | 0.058 | -0.17 |
| vanillactate | Gr3 | Tyrosine Metabolism | 0.017 | 0.099 | -0.18 |
| 2-hydroxysebacate | Gr1 | Fatty Acid, Dicarboxylate | 0.027 | 0.137 | -0.19 |
| cis-3,4-methyleneheptanoylglycine | Gr1 | Fatty Acid Metabolism (Acyl Glycine) | 0.064 | 0.242 | -0.20 |
| propionylglycine | Gr1 | Fatty Acid Metabolism (also BCAA Metabolism) | 0.049 | 0.209 | -0.21 |
| 2-palmitoleoyl-GPC (16:1) | Gr1 | Lysophospholipid | 0.002 | 0.024 | -0.22 |
| 1-arachidonylglycerol (20:4) | Gr1 | Monoacylglycerol | <0.001 | 0.005 | -0.32 |
| isoursodeoxycholate | Gr1 | Secondary Bile Acid Metabolism | 0.045 | 0.198 | -0.45 |
| 1-docosahexaenoylglycerol (22:6) | Gr1 | Monoacylglycerol | <0.001 | 0.005 | -0.50 |
| N-acetylglucosamine conjugate of C24H40O4 bile acid | Gr8 | Partially Characterized Molecules | 0.005 | 0.046 | -0.50 |
| isoursodeoxycholate sulfate (1) | Gr1 | Secondary Bile Acid Metabolism | 0.005 | 0.046 | -0.52 |
| ursodeoxycholate | Gr1 | Secondary Bile Acid Metabolism | 0.034 | 0.162 | -0.63 |

**Table S5. Abundance changes in metabolites between Controls with LIRAD-3 lesions (Controls-LR3) and Cases with LIRAD-3 lesions (Cases-LR3).** Metabolites among the 150 HCC-associated

metabolites identified in **Table S2**, which had significant differential abundance between Cases-LR3 and Controls-LR3, as determined by linear mixed-effects model analysis, are listed. Super-pathway groups for metabolites are abbreviated as follows: Gr1: Lipids, Gr2: Xenobiotics, Gr3: Amino Acids, Gr5: Peptides, Gr6: Nucleotides, Gr7: Carbohydrates. AFP: Alpha-fetoprotein, GPE: Glycerophosphorylethanolamine, GPC: Glycerophosphorylcholine, C: group coefficient (log-transformed). Metabolites are ordered from highest to lowest C.

| Metabolite | Group | Pathway | p | q | C |
|---|---|---|---|---|---|
| 1,2-dilinoleoyl-GPE (18:2/18:2) | Gr1 | Phosphatidylethanolamine (PE) | 0.020 | 0.231 | 0.71 |
| Malonate | Gr1 | Fatty Acid Synthesis | 0.007 | 0.186 | 0.71 |
| AFP | | | 0.043 | 0.268 | 0.51 |
| Pipecolate | Gr3 | Lysine Metabolism | 0.017 | 0.212 | 0.48 |
| Picolinate | Gr3 | Tryptophan Metabolism | 0.013 | 0.204 | 0.43 |
| Pyruvate | Gr7 | Glycolysis, Gluconeogenesis, and Pyruvate Metabolism | 0.011 | 0.188 | 0.42 |
| gamma-glutamyltyrosine | Gr5 | Gamma-glutamyl Amino Acid | 0.033 | 0.239 | 0.30 |
| beta-alanine | Gr6 | Pyrimidine Metabolism, Uracil containing | 0.011 | 0.188 | 0.26 |
| methionine sulfoxide | Gr3 | Methionine, Cysteine, SAM and Taurine Metabolism | 0.015 | 0.204 | 0.24 |
| gamma-glutamyltryptophan | Gr5 | Gamma-glutamyl Amino Acid | 0.029 | 0.237 | 0.23 |
| 1-palmitoyl-GPC (16:0) | Gr1 | Lysophospholipid | 0.006 | 0.186 | -0.20 |
| N-acetylglycine | Gr3 | Glycine, Serine and Threonine Metabolism | 0.027 | 0.237 | -0.24 |
| GPC | Gr1 | Phospholipid Metabolism | 0.011 | 0.188 | -0.28 |
| phenylalanylglycine | Gr5 | Dipeptide | 0.044 | 0.268 | -0.30 |
| arachidonoyl ethanolamide | Gr1 | Endocannabinoid | 0.028 | 0.238 | -0.34 |
| 1-arachidonoyl-GPC (20:4n6) | Gr1 | Lysophospholipid | 0.005 | 0.186 | -0.41 |
| N-octanoylglycine | Gr1 | Fatty Acid Metabolism (Acyl Glycine) | 0.028 | 0.238 | -0.50 |
| 1-(1-enyl-palmitoyl)-GPC (P-16:0) | Gr1 | Lysoplasmalogen | <0.001 | 0.024 | -0.50 |
| palmitoylcholine | Gr1 | Fatty Acid Metabolism (Acyl Choline) | 0.031 | 0.237 | -0.54 |
| docosahexaenoylcholine | Gr1 | Fatty Acid Metabolism (Acyl Choline) | 0.037 | 0.255 | -0.80 |
| arachidonoylcholine | Gr1 | Fatty Acid Metabolism (Acyl Choline) | 0.006 | 0.186 | -0.86 |
| dihomo-linolenoyl-choline | Gr1 | Fatty Acid Metabolism (Acyl Choline) | 0.001 | 0.078 | -0.86 |
| phenol sulfate | Gr3 | Tyrosine Metabolism | 0.021 | 0.231 | -0.98 |

## Supplementary results

### Effect of treatment on identified metabolites changes

The inclusion in this study of samples collected after HCC diagnosis and treatment for some of the patients who developed HCC during surveillance (Cases-T), allowed us to determine for which of the metabolites identified as significantly increased or decreased in Cases vs Controls, a reverse phenotype was observed after treatment. AFP was not significantly decreased in Cases-T compared to Cases. Among the 64 metabolites significantly increased in Cases vs Controls, 33 showed significant abundance decrease after treatment in Cases-T vs Cases (**Supplementary Results Table; Supplementary Results Figure panel A**). Significance remained after adjusting for FDR for 32 of these 33 metabolites (**Supplementary Results Figure panel A**). These were largely metabolites associated with Amino Acids metabolism, Bile Acid metabolism and Purine & Pyrimidine metabolism, supporting a major role of these pathways in liver progression from cirrhosis to HCC. The largest decreases were observed for tauro-conjugated bile acids: taurohyocholate ($C=-2.80$, $p<0.001$, $q<0.001$), taurocholate ($C=-2.77$, $p<0.001$, $q<0.001$), taurochenodeoxycholate ($C=-2.22$, $p<0.001$, $q<0.001$), taurochenodeoxycholic acid 3-sulfate ($C=-2.06$, $p<0.001$, $q<0.001$) and taurocholenate sulfate ($C=-1.15$, $p<0.001$, $q<0.001$). Among the 86 metabolites significantly decreased in Cases vs Controls, 44 showed significant abundance increase after treatment in Cases-T vs Cases, with 36 of them remaining significant after adjusting for FDR (**Supplementary Results Table; Supplementary Results Figure panel A**). The largest increase was observed for isoursodeoxycholate ($C=2.09$, $p<0.001$, $q<0.001$). Interestingly, positive interactions with time to last visit in Cases and inverse negative interactions with time in Cases-T, were observed in some of the metabolites from **Supplementary Results Table**. These included homoarginine and the tauro-conjugated bile acids taurocholate, taurochenodeoxycholate and taurohyocholate (**Supplementary Results Figure panel B**).

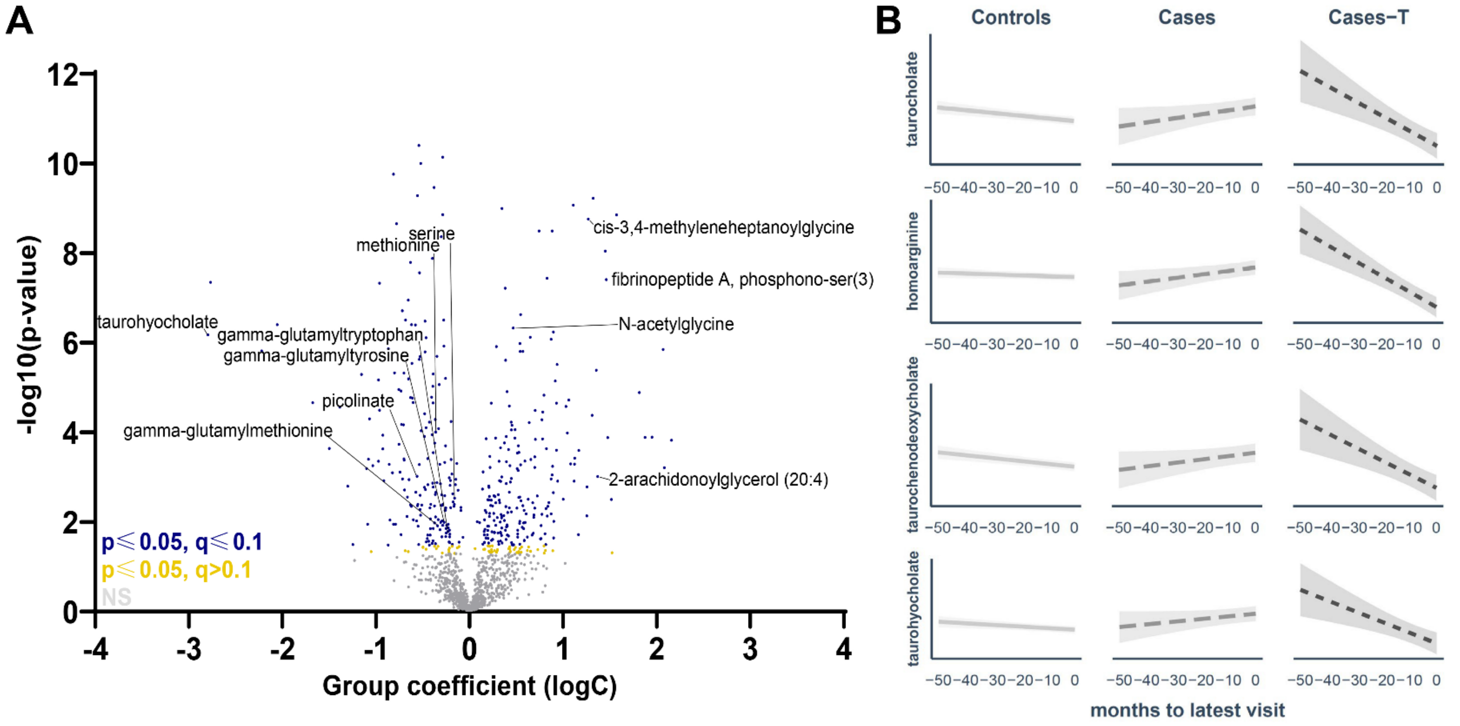


**Supplementary Results Figure. Metabolite abundance changes in Cases following HCC treatment. (A)** Volcano plot for differential metabolites, between Cases (samples collected pre-HCC) and Cases-T (samples collected after HCC treatment). The group coefficient (Cases-T vs Cases) for log-transformed metabolite levels (x-axis) and minus log10 p-values (y-axis) are shown. Metabolites that remained significant (p≤0.05, q≤0.1) after adjustment with the Benjamini–Hochberg method are shown in blue. Metabolites that were not significant (p≤0.05, q>0.1) after FDR adjustment are shown in yellow. **(B)** Interaction plots showing metabolite levels over time by group, with regression line and 95% confidence intervals. Metabolite level were plotted against months to latest visit for selected metabolites; taurocholate, within Controls, Cases and Cases-T.

**Supplementary Results Table. Abundance changes in metabolites from Table S2 after HCC treatment.** Significance of metabolites abundance changes between cases (samples collected prior to HCC diagnosis) and cases-T (samples collected after HCC treatment) was determined by linear mixed model. Super-pathway groups for metabolites are abbreviated as follows: Gr1: Lipids, Gr2: Xenobiotics, Gr3: Amino Acids: Gr4: Cofactors and Vitamins, Gr5: Peptides, Gr6: Nucleotides, Gr7: Carbohydrates,

Gr8: Partially Characterized Molecules. Cases (%) and Cases-T (%) reflect the percentage of detection of each metabolite in cases and cases-T samples, respectively. GPA: Glycerophosphatidic acid, CEHC: carboxyethyl-hydroxychroman, logC: group coefficient (log values). The metabolites are ordered from highest to lowest logC.

| Metabolite | Group | Pathway | Cases (%) | Cases-T (%) | p | q | logC |
|---|---|---|---|---|---|---|---|
| isoursodeoxycholate | Gr1 | Secondary Bile Acid Metabolism | 93.2 | 96.8 | <0.001 | <0.001 | 2.068 |
| daidzein sulfate (2) | Gr2 | Food Component/Plant | 25.0 | 45.2 | <0.001 | <0.001 | 1.814 |
| fibrinopeptide A, phosphono-ser(3) | Gr5 | Fibrinogen Cleavage Peptide | 80.7 | 90.3 | <0.001 | <0.001 | 1.461 |
| 2-arachidonoylglycerol (20:4) | Gr1 | Monoacylglycerol | 38.6 | 83.9 | 0.001 | 0.007 | 1.370 |
| glycine conjugate of C10H12O2 | Gr8 | Partially Characterized Molecules | 70.5 | 93.5 | <0.001 | <0.001 | 1.322 |
| cysteine-glutathione disulfide | Gr3 | Glutathione Metabolism | 84.1 | 100 | <0.001 | 0.001 | 1.310 |
| cis-3,4-methyleneheptanoylglycine | Gr1 | Fatty Acid Metabolism (Acyl Glycine) | 93.2 | 100 | <0.001 | <0.001 | 1.268 |
| N6-carboxymethyllysine | Gr7 | Advanced Glycation End-product | 60.2 | 90.3 | 0.002 | 0.010 | 1.256 |
| ursodeoxycholate | Gr1 | Secondary Bile Acid Metabolism | 93.2 | 90.3 | 0.007 | 0.032 | 1.254 |
| trimethylamine N-oxide | Gr1 | Phospholipid Metabolism | 100 | 100 | <0.001 | 0.003 | 1.125 |
| 6-hydroxyindole sulfate | Gr2 | Chemical | 96.6 | 100 | 0.001 | 0.004 | 1.121 |
| isoursodeoxycholate sulfate (1) | Gr1 | Secondary Bile Acid Metabolism | 53.4 | 77.4 | 0.001 | 0.008 | 1.117 |
| metabolonic lactone sulfate | Gr8 | Partially Characterized Molecules | 53.4 | 58.1 | 0.001 | 0.004 | 1.080 |
| dimethylguanidino valeric acid | Gr3 | Urea cycle; Arginine and Proline Metabolism | 69.3 | 77.4 | 0.001 | 0.008 | 0.942 |
| androstenediol (3beta,17beta) disulfate (1) | Gr1 | Androgenic Steroids | 100 | 100 | <0.001 | <0.001 | 0.917 |
| 7-methylxanthine | Gr2 | Xanthine Metabolism | 86.4 | 83.9 | 0.002 | 0.013 | 0.897 |
| 3-indoxyl sulfate | Gr3 | Tryptophan Metabolism | 100.0 | 100 | 0.001 | 0.006 | 0.883 |
| 4-acetylphenol sulfate | Gr2 | Benzoate Metabolism | 85.2 | 93.5 | 0.007 | 0.030 | 0.871 |
| hexanoylglycine | Gr1 | Fatty Acid Metabolism (Acyl Glycine) | 21.6 | 64.5 | <0.001 | <0.001 | 0.871 |
| androstenediol (3beta,17beta) monosulfate (1) | Gr1 | Androgenic Steroids | 87.5 | 80.6 | 0.004 | 0.022 | 0.836 |
| 4-methylcatechol sulfate | Gr2 | Benzoate Metabolism | 98.9 | 100 | 0.050 | 0.133 | 0.798 |
| retinal | Gr4 | Vitamin A Metabolism | 90.9 | 100 | <0.001 | <0.001 | 0.796 |
| isobutyrylglycine | Gr3 | Leucine, Isoleucine and Valine Metabolism | 48.9 | 64.5 | <0.001 | <0.001 | 0.783 |
| acisoga | Gr3 | Polyamine Metabolism | 88.6 | 100 | <0.001 | 0.001 | 0.781 |
| 1,5-anhydroglucitol (1,5-AG) | Gr7 | Glycolysis, Gluconeogenesis, and Pyruvate Metabolism | 100 | 100 | <0.001 | 0.001 | 0.703 |
| pimeloylcarnitine/3-methyladipoylcarnitine (C7-DC) | Gr1 | Fatty Acid Metabolism (Acyl Carnitine, Dicarboxylate) | 88.6 | 100 | 0.001 | 0.005 | 0.696 |
| glycine conjugate of C10H14O2 (1) | Gr8 | Partially Characterized Molecules | 100 | 100 | <0.001 | 0.000 | 0.649 |

| | | | | | | | |
|---|---|---|---|---|---|---|---|
| chenodeoxycholic acid sulfate (1) | Gr1 | Primary Bile Acid Metabolism | 51.1 | 77.4 | 0.036 | 0.107 | 0.632 |
| epiandrosterone sulfate | Gr1 | Androgenic Steroids | 100 | 100 | 0.007 | 0.030 | 0.605 |
| docosahexaenoylcholine | Gr1 | Fatty Acid Metabolism (Acyl Choline) | 68.2 | 90.3 | 0.026 | 0.084 | 0.585 |
| N-acetylglucosamine conjugate of C24H40O4 bile acid | Gr8 | Partially Characterized Molecules | 48.9 | 83.9 | 0.036 | 0.107 | 0.556 |
| arachidonoylcholine | Gr1 | Fatty Acid Metabolism (Acyl Choline) | 94.3 | 100 | 0.047 | 0.129 | 0.553 |
| 2-hydroxyphenylacetate | Gr3 | Phenylalanine Metabolism | 83.0 | 100 | 0.003 | 0.018 | 0.550 |
| gulonate | Gr4 | Ascorbate and Aldarate Metabolism | 94.3 | 100 | <0.001 | <0.001 | 0.546 |
| 4-hydroxyhippurate | Gr2 | Benzoate Metabolism | 100 | 100 | 0.010 | 0.040 | 0.536 |
| delta-CEHC | Gr4 | Tocopherol Metabolism | 84.1 | 90.3 | 0.003 | 0.016 | 0.536 |
| 5alpha-androstan-3beta,17beta-diol disulfate | Gr1 | Androgenic Steroids | 84.1 | 80.6 | 0.050 | 0.134 | 0.528 |
| 3,7-dimethylurate | Gr2 | Xanthine Metabolism | 54.5 | 71.0 | 0.046 | 0.126 | 0.526 |
| N-acetyl-aspartyl-glutamate | Gr3 | Glutamate Metabolism | 89.8 | 93.5 | <0.001 | 0.001 | 0.486 |
| N-acetylglycine | Gr3 | Glycine, Serine and Threonine Metabolism | 100 | 100 | <0.001 | <0.001 | 0.467 |
| 1-arachidonoyl-GPC (20:4n6) | Gr1 | Lysophospholipid | 100 | 100 | 0.001 | 0.007 | 0.447 |
| arachidonoyl ethanolamide | Gr1 | Endocannabinoid | 30.7 | 87.1 | <0.001 | 0.001 | 0.447 |
| N-acetyltaurine | Gr3 | Methionine, Cysteine, SAM and Taurine Metabolism | 98.9 | 100 | 0.034 | 0.104 | 0.243 |
| vanillactate | Gr3 | Tyrosine Metabolism | 96.6 | 93.5 | 0.047 | 0.128 | 0.214 |
| lysine | Gr3 | Lysine Metabolism | 100 | 100 | <0.001 | 0.004 | -0.136 |
| serine | Gr3 | Glycine, Serine and Threonine Metabolism | 100 | 100 | 0.003 | 0.016 | -0.160 |
| phenylalanine | Gr3 | Phenylalanine Metabolism | 100 | 100 | 0.002 | 0.014 | -0.179 |
| asparagine | Gr3 | Alanine and Aspartate Metabolism | 100 | 100 | 0.002 | 0.010 | -0.208 |
| threonine | Gr3 | Glycine, Serine and Threonine Metabolism | 100 | 100 | 0.001 | 0.007 | -0.217 |
| methylphosphate | Gr6 | Purine and Pyrimidine Metabolism | 100 | 100 | 0.011 | 0.044 | -0.227 |
| gamma-glutamyltryptophan | Gr5 | Gamma-glutamyl Amino Acid | 100 | 100 | 0.016 | 0.059 | -0.233 |
| 1-(1-enyl-palmitoyl)-2-oleoyl-GPE (P-16:0/18:1) | Gr1 | Plasmalogen | 98.9 | 100 | 0.012 | 0.046 | -0.246 |
| adenine | Gr6 | Purine Metabolism, Adenine containing | 100 | 100 | <0.001 | 0.002 | -0.257 |
| gamma-glutamyltyrosine | Gr5 | Gamma-glutamyl Amino Acid | 100 | 100 | 0.010 | 0.041 | -0.272 |
| methionine sulfoxide | Gr3 | Methionine, Cysteine, SAM and Taurine Metabolism | 100 | 100 | 0.010 | 0.039 | -0.294 |
| 4-hydroxyphenylpyruvate | Gr3 | Tyrosine Metabolism | 100 | 100 | 0.021 | 0.073 | -0.296 |
| gamma-glutamylmethionine | Gr5 | Gamma-glutamyl Amino Acid | 100 | 100 | 0.012 | 0.047 | -0.341 |
| methionine | Gr3 | Methionine, Cysteine, SAM and Taurine Metabolism | 100 | 100 | <0.001 | 0.001 | -0.361 |
| phytanate | Gr2 | Food Component/Plant | 100 | 100 | 0.035 | 0.105 | -0.375 |
| testosterone sulfate | Gr1 | Androgenic Steroids | 53.4 | 29.0 | 0.032 | 0.099 | -0.430 |
| sphingosine | Gr1 | Sphingosines | 98.9 | 96.8 | <0.001 | 0.001 | -0.430 |
| alpha-hydroxyisocaproate | Gr3 | Leucine, Isoleucine and Valine Metabolism | 100 | 100 | <0.001 | 0.003 | -0.446 |

| | | | | | | | |
|---|---|---|---|---|---|---|---|
| 1-linoleoyl-GPA (18:2) | Gr1 | Lysophospholipid | 100 | 100 | 0.002 | 0.011 | -0.471 |
| tyrosine | Gr3 | Tyrosine Metabolism | 100 | 100 | <0.001 | <0.001 | -0.475 |
| picolinate | Gr3 | Tryptophan Metabolism | 100 | 100 | 0.001 | 0.007 | -0.562 |
| 2'-O-methylcytidine | Gr6 | Pyrimidine Metabolism, Cytidine containing | 100 | 90.3 | <0.001 | <0.001 | -0.656 |
| 5,6-dihydrouracil | Gr6 | Pyrimidine Metabolism, Uracil containing | 96.6 | 83.9 | 0.008 | 0.033 | -0.668 |
| hexadecanedioate (C16-DC) | Gr1 | Fatty Acid, Dicarboxylate | 98.9 | 100 | <0.001 | 0.003 | -0.702 |
| pristanate | Gr1 | Fatty Acid, Branched | 68.2 | 77.4 | 0.019 | 0.067 | -0.732 |
| homoarginine | Gr3 | Urea cycle; Arginine and Proline Metabolism | 100 | 100 | <0.001 | <0.001 | -0.814 |
| beta-sitosterol | Gr1 | Sterol | 71.6 | 83.9 | <0.001 | <0.001 | -0.868 |
| taurocholenate sulfate | Gr1 | Secondary Bile Acid Metabolism | 100 | 100 | <0.001 | <0.001 | -1.154 |
| glycohyocholate | Gr1 | Secondary Bile Acid Metabolism | 98.9 | 93.5 | <0.001 | <0.001 | -1.676 |
| taurochenodeoxycholic acid 3-sulfate | Gr1 | Secondary Bile Acid Metabolism | 95.5 | 96.8 | <0.001 | <0.001 | -2.056 |
| taurochenodeoxycholate | Gr1 | Primary Bile Acid Metabolism | 100 | 100 | <0.001 | <0.001 | -2.222 |
| taurocholate | Gr1 | Primary Bile Acid Metabolism | 100 | 96.8 | <0.001 | <0.001 | -2.767 |
| taurohyocholate | Gr1 | Secondary Bile Acid Metabolism | 87.5 | 61.3 | <0.001 | <0.001 | -2.796 |
